# Sulforaphane, A Mustard Oil Bomb Product, Reprograms Herbivore Gene Expression and Alters Chromatin Structure Across Generations

**DOI:** 10.1007/s10886-026-01771-x

**Published:** 2026-10-29

**Authors:** Thomas M. Arnold, Dana J. Somers, David B. Kushner, Marisa Arreola, Whitney Finney, Ashley Groff, Annika Haagensen, Mel Morales, Charlie Kim, Emi Ayala-Sekiguchi, Mason Arnold

**Affiliations:** Dickinson College, Department of Biology, Carlisle, PA 17013 USA

**Keywords:** Sulforaphane, HDAC inhibitor, Epigenetic defense, *Spodoptera exigua*, *Trichoplusia ni*, Intergenerational effects

## Abstract

Sulforaphane (SFN), an isothiocyanate produced by the mustard oil bomb defense system of cruciferous plants, is a natural inhibitor of class I and II histone deacetylases (HDACs). We previously showed that dietary SFN inhibits HDAC activity, delays larval development, and alters the expression of more than 1,600 genes in the agricultural pests *Spodoptera exigua* and *Trichoplusia ni*. Here we investigated the mechanisms underlying these responses and whether their effects extend across generations. RNA-seq analyses revealed that SFN rapidly disrupted the normal developmental progression of gene expression in both somatic and germline tissues. ATAC-seq analyses indicated that these transcriptional changes in *S. exigua* were accompanied by widespread alterations in chromatin accessibility. In this species, we detected the suppression of genes involved in energy metabolism, consistent with the observed developmental delays. We observed that many SFN-induced changes in chromatin accessibility and gene expression persisted in the offspring of exposed insects despite the offspring never consuming SFN themselves. These findings demonstrate that SFN acts through chromatin-associated mechanisms to alter transcriptional programs during development, with intergenerational effects. We propose that SFN, and perhaps other HDAC inhibitors produced by plants in nature, may act as an “epigenetic weapon” with the potential to influence herbivore gene expression across generations.

**Supplementary Information:**

The online version contains supplementary material available at https://doi.org/10.1007/s10886-026-01771-x.

## Introduction

**Fig. 1:**
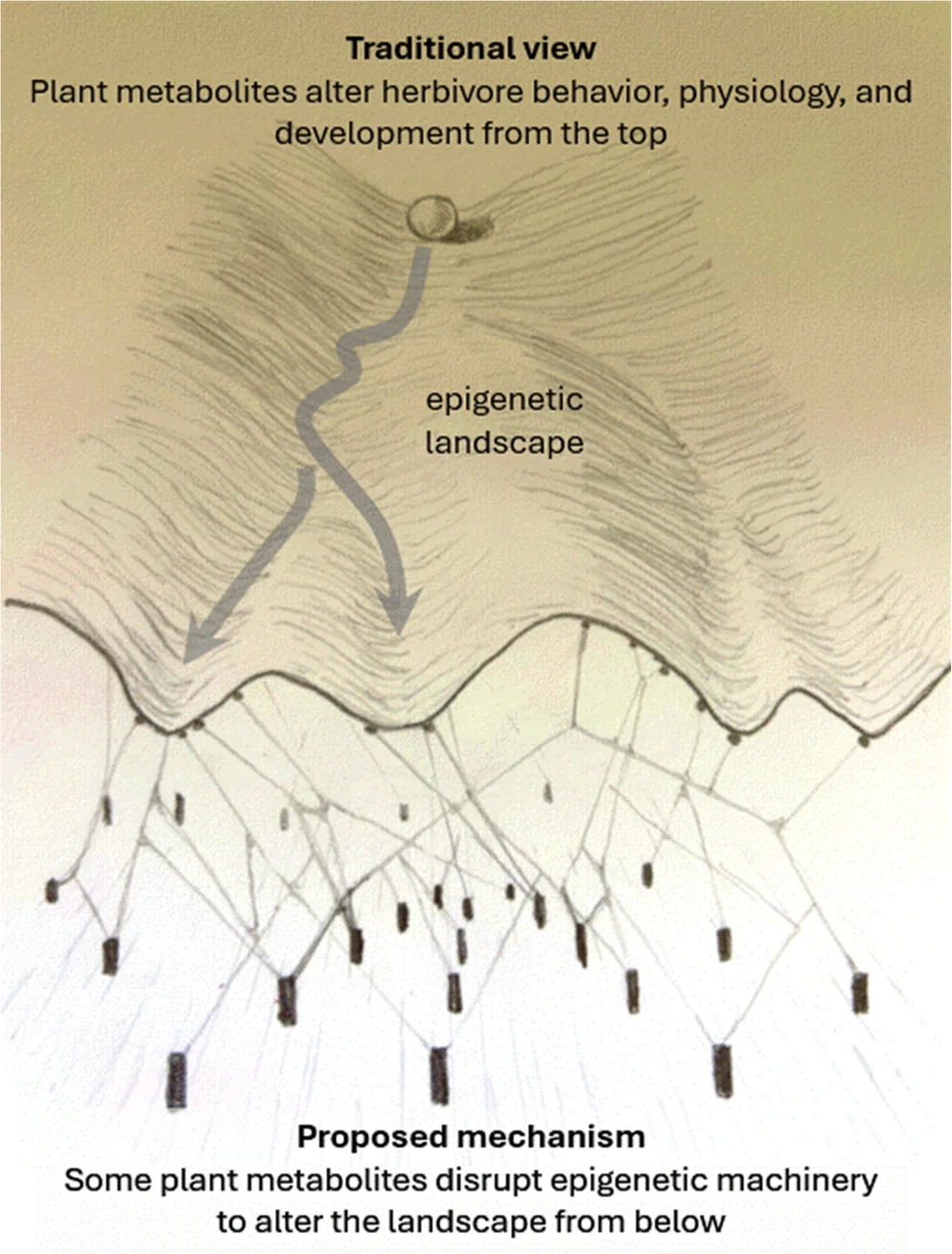
A reimagining of Waddington’s well-known epigenetic landscape diagram from *The Strategy of the Genes* (1957). The development of cells or organisms follows one or more channels in the landscape, unless their trajectory is altered by perturbations or interactions along the way. Channels are shaped by genes (pegs) connected to the landscape by epigenetic systems (guy ropes) including those controlling the accessibility of chromatin and the expression of genes. We propose that sulforaphane from the mustard oil bomb reactions of cruciferous plants can target these epigenetic systems from underneath to reshape the landscape’s contours, thus altering an herbivore’s developmental trajectory

The term “epigenetics” was coined in 1942 by Conrad Waddington to describe the developmental processes linking the genotype and phenotype (Waddington 1942, 2012; Noble 2015). In his now-famous illustration, he imagined development as a ball rolling along an epigenetic landscape following branching channels toward alternative fates (Waddington 1957). For more than half a century, chemical ecologists have primarily investigated how plant metabolites alter herbivore development, behavior, physiology, and ultimately fitness—that is, how plant chemistry influences trajectories across this epigenetic landscape. However, comparatively little attention has been given to the possibility that plant metabolites directly target the molecular systems shaping these channels. Waddington called these systems “tendencies” and imagined them as guy ropes connecting genes to the underside of the epigenetic landscape (Fig. 1). Today, these guy ropes are understood to include gene regulatory networks, chromatin structure, epigenetic modifications, transcription factors, and other mechanisms that determine patterns of gene expression (Deichmann 2016). Here we suggest that some plant secondary metabolites disrupt these epigenetic elements which shape the epigenetic landscape itself. We provide evidence that sulforaphane, a product of the mustard oil bomb defenses of cruciferous plants, inhibits histone deacetylase enzymes, alters chromatin accessibility, disrupts the expression of more than one thousand genes, and slows herbivore development. These results support the hypothesis that some plant secondary metabolites act not by altering trajectories across Waddington’s epigenetic landscape, but by targeting the molecular machinery that shapes the landscape itself.

The mustard oil bomb of cruciferous plants is a well-studied chemical defense against herbivory. It is triggered by tissue damage, which allows myrosinase enzymes (ß-thioglucoside glucohydrolases) to mix with glucosinolates to generate toxic nitriles, isothiocyanates, thiocyanates, oxazolidine-2-thiones, and epithionitriles (Fahey et al. 2001; Vaughn 2005; Halkier and Gershenzon 2006; Wittstock and Burow 2010; Angelino and Jeffery 2014). This defense system can deter grazing, especially by generalist herbivores (Mithen et al. 1995; Barth and Jander 2006; Burow et al. 2006; Beekwilder et al. 2008; Textor and Gershenzon 2009; Müller et al. 2010; Jeschke et al. 2016, 2017; Zalucki et al. 2017; Robin et al. 2017; Liu et al. 2021; Badenes-Pérez and Cartea 2021). In these reactions the alphiphatic glucosinolate glucoraphanin (4-(methylsulfinyl) butyl glucosinolate) can be converted into sulforaphane (SFN) (Dinkova-Kostova and Kostov 2012; Vanduchova et al. 2019; Santín-Márquez et al. 2019). Amongst the mustard oil bomb end-products, SFN has unique properties as an inhibitor of Class I and II histone deacetylase (HDAC) enzymes in animals (Myzak et al. 2006; Ho et al. 2009; Bassett and Barnett 2014) and these properties have been well-studied in a biomedical context (Dinkova-Kostova and Kostov 2012; Houghton et al. 2013; Vanduchova et al. 2019; Huang et al. 2019; Pu et al. 2019; Santín-Márquez et al. 2019; Sun et al. 2020; Bhattacharjee and Dashwood 2020). By contrast, the ecological effects of SFN in plant–herbivore interactions remain poorly understood, especially its potential role as a natural HDAC inhibitor acting on insect epigenetic regulation.

Previously, we explored the role of SFN in cruciferous plants and their insect pests. We demonstrated that natural concentrations of dietary SFN inhibit HDAC enzymes in the beet armyworm (*Spodoptera exigua*) but not in the cabbage looper (*Trichoplusia ni*) (Somers et al. 2023). The consequences for *S. exigua* were remarkable: the consumption of SFN altered the expression of over 1600 genes in fat body tissue and slowed larval development by 50%. In comparison, the effects of SFN on *T. ni* were noticeably weaker, perhaps because this species possesses a highly active glutathione-based detoxification system capable of mitigating the mustard oil bomb defenses (Wadleigh and Yu 1988; Winde and Wittstock 2011; Schramm et al. 2012).

Here we conducted a new series of experiments to explore how SFN impacted the epigenetic systems of these grazing herbivores and their offspring. Specifically, we tested the potential for dietary SFN to alter chromatin accessibility, disrupt gene expression, and delay development across two generations. By documenting effects of SFN on feeding caterpillars we sought to assess the ability of this metabolite to disrupt epigenetic systems and developmental trajectories, as imagined by Waddington. We also tested for intergenerational effects by examining gene expression and chromatin accessibility in offspring, which did not themselves consume SFN.

If SFN triggers metastable modifications of chromatin that are, in turn, transmitted to offspring this could be considered an intergenerational effect (Perez and Lehner 2019; Fitz-James and Cavalli 2022; Bird 2024). This would not be unprecedented as intergenerational effects have been observed, to various degrees, in a variety of insect and plant species (Gegner et al. 2019; Tando and Matsui 2023; Bird 2024; Spanou et al. 2025). Here we evaluated whether the effects of this plant defense compound persist across generations. Such persistent effects would have the potential to shape plant–herbivore interactions beyond the directly exposed generation.

## Methods

### Experimental Design

First instar larvae were obtained from Benzon Research (Carlisle, Pennsylvania, USA) and raised individually in 5 ml wells on an artificial diet (AD) appropriate for each species with antibiotics at 25 °C and 50% humidity. Larvae fed *ad lib* on diets containing SFN or trichostatin A (TSA), a more powerful pharmaceutical HDAC inhibitor which served as a positive response control, as described previously (Somers et al. 2023). HDAC inhibitors (Millipore Sigma, Burlington, Massachusetts, USA) were solubilized in ethanol which was evaporated for 8 h at 20 °C before first instar larvae were added to wells. The concentrations of SFN were similar to that ingested during natural feeding on cruciferous plant foliage (Somers et al. 2023). Specifically, we explored the effects of 70 mM SFN, applied as 30–40 µl aliquots to each well containing AD. This dose is comparable to levels reported in leaves and lower than levels reported in buds, flowers, sprouts, or seeds. They are also comparable to those used in medical studies. The dose of TSA, a stronger pharmaceutical HDAC inhibitor, was 330 µM. In multigeneration experiments we grew first generation larvae of both species simultaneously, under identical conditions, allowed subsets of larvae to pupate, emerge as moths, and mate within treatment groups. Second-generation larvae were then reared on AD without HDAC inhibitors. To detect potential intergenerational effects, we compared second generation offspring from parents exposed to SFN to those from parents who were not exposed to this substance. In all experiments, we digitally monitored the development of larvae. Larval sizes and developmental milestones were compared using Student’s t-tests or ANOVAs with Holm-Sidak posthoc tests. Non-parametric alternatives, including Mann-Whitney U tests and ANOVAs on ranks with Dunn’s pairwise comparisons, were used when datasets were not normally distributed. At various time intervals fat body and/or ovary tissues were collected and preserved in RNAlater (Invitrogen, San Diego, California, USA; ThermoFisher Scientific, Waltham, Massachusetts, USA) using 1 ml per 100 mg tissue, and stored at -80 °C, as described previously (Somers et al. 2023).

### RNA Extraction, Library Construction, and Sequencing

Total RNA was extracted from the tissue as described in detail previously (Somers et al. 2023). RNA concentration was determined using a DS-11 spectrophotometer (DeNovix, Wilmington, Delaware, USA). Total RNA quality was assessed using RNA Nano Chips and a 2100 BioAnalyzer (Agilent, Santa Clara, California, USA) and Qubit RNA High Sensitivity Assay (ThermoFisher, Waltham, Massachusetts, USA), followed by High Sensitivity RNA ScreenTape Analysis (Agilent, Santa Clara, California, USA).

Library construction and sequencing was performed by Admera Health Biopharma Services (South Plainfield, New Jersey, USA), as described in the Supplemental Methods.

### RNA-seq Processing and Analysis

Reads were quality checked and pre-processed, as described previously (Somers et al. 2023). *T. ni* reads were aligned to the *T. ni* Cornell-1 isolate genome and *S. exigua* reads were aligned to the *S. exigua* PGI_SPEXI_v6 genome assembly (GCA_902829305.4) (Chen et al. 2019; Simon et al. 2021). Reads were aligned using HISAT2 (v2.2.1) with the default parameters, and alignments were quality checked using QualiMap (v2.2.1) (Okonechnikov et al. 2016). featureCounts implemented in the Rsubread package (v2.12.2) was used to calculate read counts for each gene using the appropriate species-specific annotations (Liao et al. 2019).

Low count genes (mean counts-per-million [CPM] across all samples > 10) were filtered and read counts were normalized using the trimmed mean of M-value normalization method. Differential expression analysis was performed with DESeq2 (v1.50.2) (Love et al. 2014) by modeling gene expression counts as a smooth function of time with spline-based regression with an interaction term to capture treatment-specific effects. Significance was assessed via log-likelihood ratio test with the negative binomial, and taking the Benjamini and Hochberg false discovery rate (FDR) < 0.05 as significant.

KEGG and GO annotation terms were assigned to each genome using eggNOG (v2.1.7) (Cantalapiedra et al. 2021). Functional enrichment analysis of gene lists was performed with topGO (v2.60.1) (Alexa and Rahnenfuhrer 2012) employing the weight01 algorithm with Fisher’s exact test to account for the hierarchical structure of GO, and taking as background the set of genes in each species that were retained after filtering for low count genes. To reduce redundancy among semantically related GO terms and improve visualization clarity, enriched GO categories were manually curated into representative biological themes based on statistical significance, fold enrichment, recurrence among related terms, and biological interpretability. Representative GO terms were selected for visualization, while redundant or weakly informative categories were omitted. Complete GO enrichment results are provided in the Data Supplement.

### ATAC-seq Nuclei Extraction, Library Construction, and Sequencing

ATAC-seq was performed by Admera Health Biopharma Services (South Plainfield, New Jersey, USA) as previously described (Corces et al. 2017). Briefly, frozen tissue was thawed in cold homogenization buffer on ice for 5 min. Tissue dissociation was performed in Nuclei Extraction Buffer (Miltenyi Biotec, Bergisch Gladbach, Germany) with the gentleMACS Tissue Dissociator (Miltenyi Biotec, Bergisch Gladbach, Germany) following the manufacturer’s protocol. Nuclei number was assessed by Cellometer Auto 2000 (Nexcelom Bioscience, Massachusetts, USA). 50 K to 100 K cells were mixed with 1 mL cold ATAC resuspension buffer for 5 min on ice and centrifuged at 500 RCF in a pre-chilled centrifuge for 10 min. Supernatant was removed and nuclei were resuspended in 50 uL tagmentation reaction buffer by pipetting up and down gently. The reactions were incubated at 37 °C for 30 min in a thermomixer with shaking at 1,000 RPM and cleaned using the MinElute Reaction Cleanup Kit (Qiagen, Hilden, Germany) following the manufacturer’s protocol. Tagmented DNA was amplified with barcode primers. Library quality and quantity were assessed with Qubit 2.0 DNA HS Assay (ThermoFisher, Massachusetts, USA), Tapestation High Sensitivity D1000 Assay (Agilent Technologies, California, USA), and QuantStudio ^®^ 5 System (Applied Biosystems, California, USA). Equimolar pooling of libraries was performed based on QC values and sequenced on an Illumina^®^ NovaSeq X Plus 10B (Illumina, California, USA) with a read length configuration of 150-bp for 50 M paired-end reads (25 M in each direction) per sample.

### ATAC-seq Processing and Analysis

ATAC-seq reads were processed using the ENCODE ATAC-seq pipeline (v2.2.3) with default parameters (Kim 2023; Hitz et al. 2023). Briefly, sequencing adapters were removed, and reads were aligned to the *S. exigua* reference genome (GCA_902829305.4) using Bowtie2 (v2.4.5). PCR duplicates and low-quality alignments were removed, mitochondrial reads were filtered, and properly paired reads were retained for downstream analyses. Peaks were identified using MACS2 (v2.2.4), and quality-control metrics, including fragment length distributions, transcription start site (TSS) enrichment, fraction of reads in peaks (FRiP), and library complexity, were generated for each sample. Consensus peak sets were used for all subsequent differential accessibility analyses.

Differential chromatin accessibility was assessed using the DiffBind package (v3.20.0) in R (Stark and Brown 2012; Ross-Innes et al. 2012). Consensus peak sets generated by the ENCODE pipeline were quantified across all samples, and differential accessibility was evaluated separately for each generation using DESeq2 (v1.50.2) (Love et al. 2014). Peaks with a false discovery rate (FDR) < 0.10 were considered significantly differentially accessible.

Significant and consensus peaks were annotated relative to gene features using the ChIPseeker (v1.42.0) (Wang et al. 2022) package with a custom *S. exigua* TxDb annotation. Promoter regions were defined as ± 3 kb surrounding annotated transcription start sites. Peaks were classified as promoter, intronic, exonic, downstream, untranslated region, or distal intergenic based on their genomic location.

### Data Availability

For the RNA-seq analyses, all raw sequence reads were deposited in the NIH Sequence Read Archive (SRA) under BioProject numbers PRJNA1497926 (*S. exigua*) and PRJNA1498032 (*T. ni*). For ATAC-seq analyses, all raw sequence reads were deposited in the NIH Sequence Read Archive (SRA) under BioProject number PRJNA1498575.

## Results

### Experiment 1. The Timing of Larval Responses to SFN

**Fig. 2:**
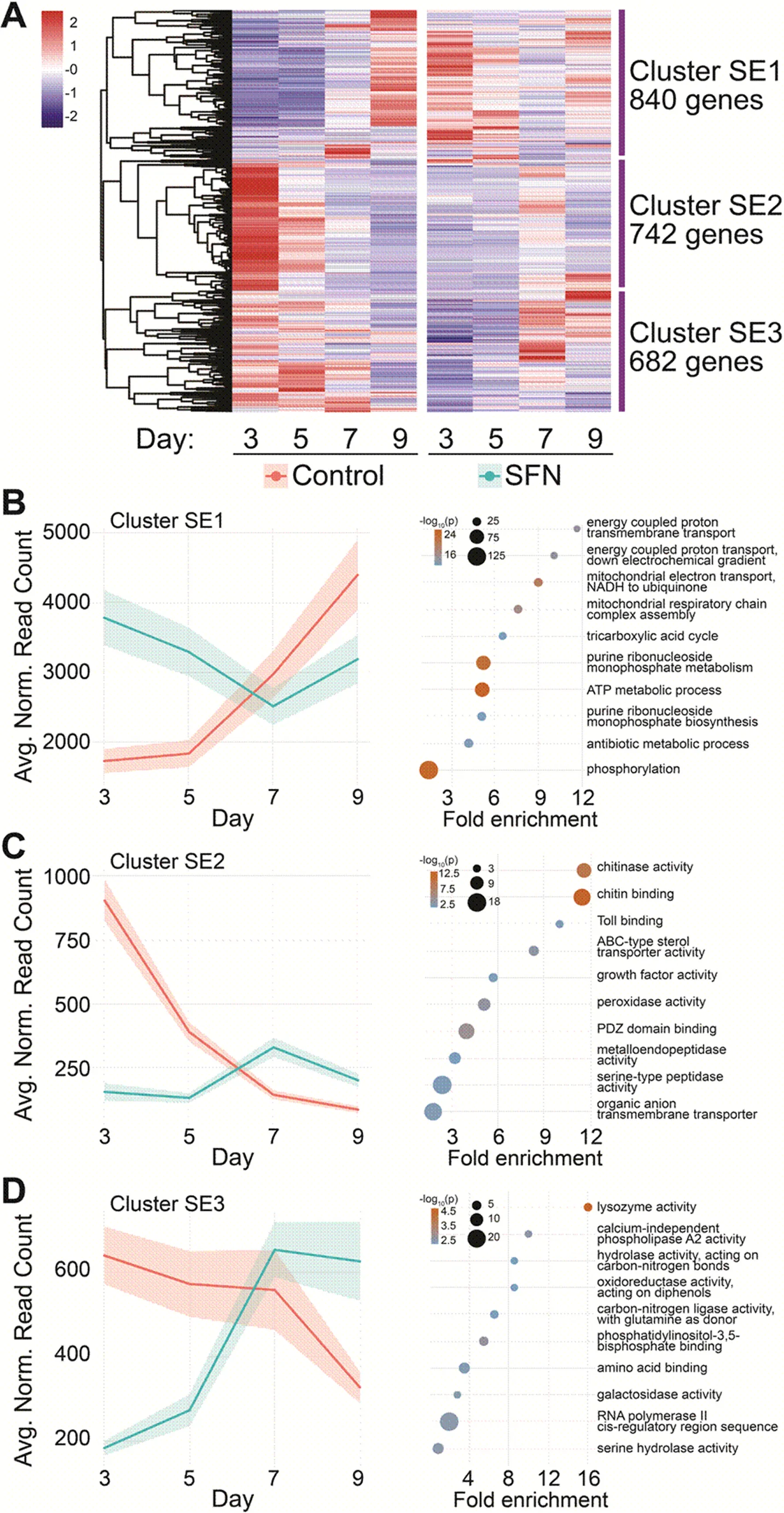
SFN disrupts gene expression in fat body tissue throughout larval development in *S. exigua*. **A** Hierarchical clustering of 2,264 genes with SFN-sensitive expression differences over time. Values are row-scaled such that red indicates higher expression and blue indicates lower expression relative to each gene’s mean expression across all samples. Clustering was performed using the Pearson correlation and complete linkage. **B-D** Average expression profiles and Gene Ontology (GO) enrichment for genes within each cluster. Shaded regions represent ±1 standard error of the mean expression value across all genes for each condition within a cluster. The top ten most enriched GO terms are ordered based on enrichment, where point size indicates the number of genes associated with each term, and color represents statistical significance. Complete enrichment results are available in Supplemental Tables S1-S6

**Fig. 3:**
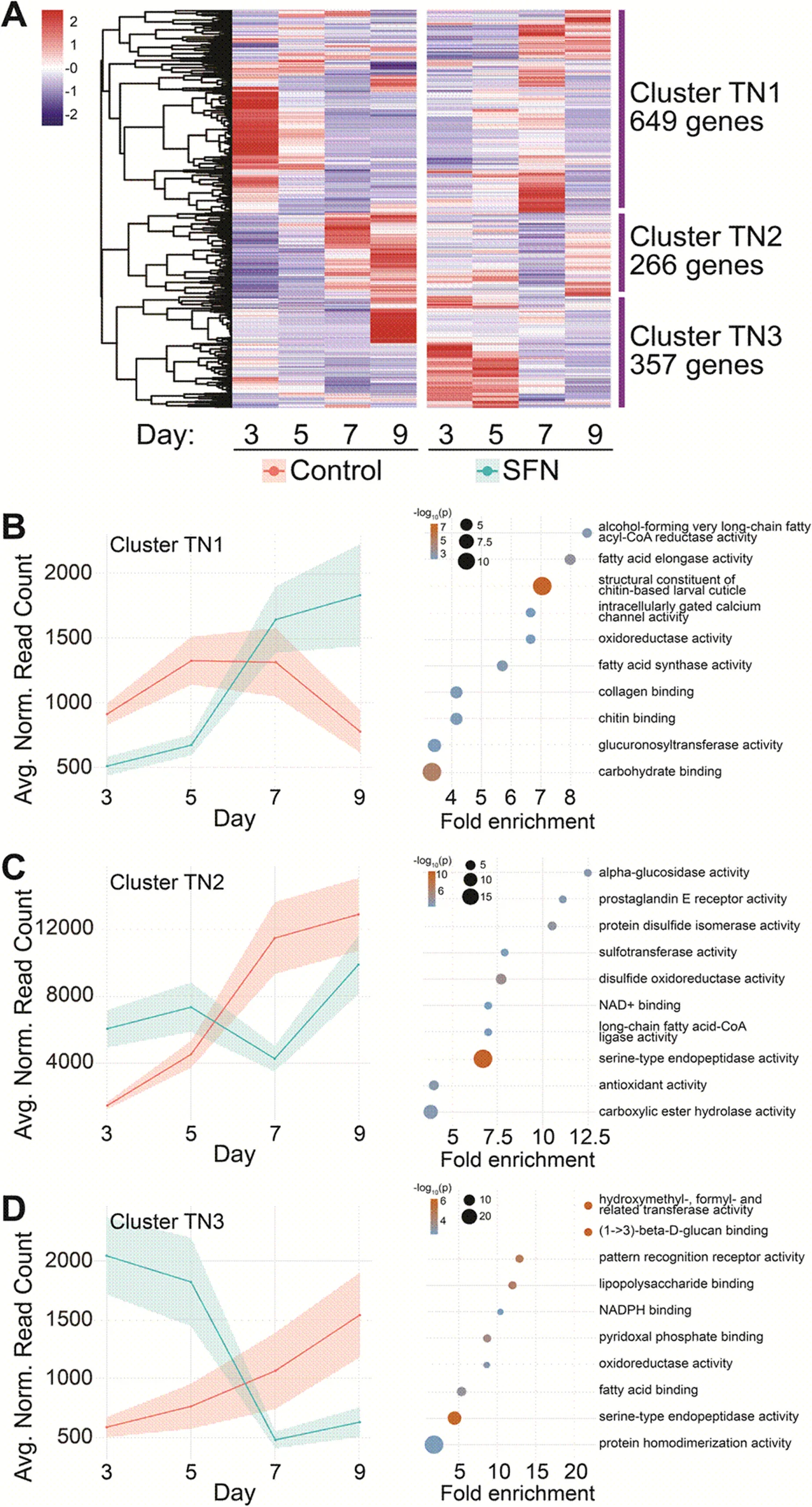
SFN disrupts gene expression in fat body tissue throughout larval development in *T. ni*. **A** Hierarchical clustering of 1,272 genes with SFN-sensitive expression differences over time. Values are row-scaled such that red indicates higher expression and blue indicates lower expression relative to each gene’s mean expression across all samples. Clustering was performed using the Pearson correlation and complete linkage. **B-D** Average expression profiles and Gene Ontology (GO) enrichment for genes within each cluster. Shaded regions represent ±1 standard error of the mean expression value across all genes for each condition within a cluster. The top ten most enriched GO terms are ordered based on enrichment, where point size indicates the number of genes associated with each term, and color represents statistical significance. Complete enrichment results are available in Supplemental Tables S7-S12

We measured differential gene expression in fat body tissues of *S. exigua* and *T. ni* 3, 5, 7, and 9 days after larvae began consuming SFN (Supplemental Fig. S1). We focused our analyses by considering only genes expressed during larval development, filtering those with low-counts. Next, we identified clusters of genes that responded similarly to SFN during the larval period via hierarchical clustering, in both *S. exigua* (Fig. 2) and *T. ni* (Fig. 3).

In *S. exigua*, we sorted 2,264 genes with SFN-sensitive expression differences into three clusters. In cluster *S. exigua* 1 (SE1) we identified 840 genes which typically increase in expression over the course of larval development but here were disrupted by SFN. This cluster contains an unusual number of genes associated with energy metabolism, including oxidative phosphorylation, electron transport, ATP synthesis, glycolysis, and the TCA cycle as well as genes associated with translation and peptide biosynthesis pathways (Fig. 2B). This suggests that SFN suppresses genes associated with energy production and transformation in larvae feeding on this diet, at least at many of the time points we examined. Cluster SE2 included 742 genes normally expressed in early larval development (e.g., at days 3 and 5) that were suppressed by SFN during this phase (Fig. 2C). This cluster includes genes associated with chitin metabolism and cuticle development as well as epithelial morphogenesis and tissue development. This suggests that SFN negatively impacts structural remodeling and developmental processes common in larvae of this stage of development in *S. exigua*. Cluster SE3 included genes that normally decline in expression during larval development but, in these cases, responded to SFN with abnormally increased expression (Fig. 2D). This cluster includes genes related to signaling and developmental regulation, indicating that SFN may induce or prolong regulatory and signaling programs that are normally downregulated during this developmental window. Complete enrichment results are available in Supplemental Tables S1-S6.

In *T. ni* we identified 1,272 genes impacted by SFN which exhibited statistically significant differences at one or more points during larval development (Fig. 3A). These were also sorted into three clusters, based on their response patterns. For this species, cluster *T. ni* 1 (TN1) included 649 genes which responded to SFN by intensifying expression later in larval development, just days before pupation. This cluster was enriched for genes associated with structural and developmental processes such as cuticle formation and tissue differentiation (Fig. 3B). In clusters 2 and 3 we identified two subsets of genes that normally increase in expression through the course of larval development but were disrupted, in different ways, by exposure to SFN. In cluster TN2 a total of 266 genes which normally increase in expression early in development (e.g., between days 3 and 5) showed disrupted expression in response to SFN exposure. This cluster included genes associated with lipid metabolism and redox-related processes, suggesting a role in metabolism that is disrupted by SFN treatment (Fig. 3C). In cluster TN3 we identified 357 genes which typically increase in expression later in development (e.g., at days 7 and 9) but showed reduced expression in SFN-treated larvae. This cluster was enriched for a broad range of enzymatic and biosynthetic activities, indicating that SFN may suppress general metabolic and biosynthetic functions during development (Fig. 3D). Complete enrichment results are available in Supplemental Tables S7-S12.

Together, these results indicate that SFN works quickly to alter the normal patterns of larval gene expression within 3–9 days following exposure *via* the diet. SFN affected over 1000 genes, including those related to metabolic activity, structural remodeling, and regulatory signaling pathways. In most cases, these changes in gene expression started to “bounce back” by day 9, suggesting that certain gene expression patterns are reset, at least partially, before the next developmental stage (e.g., pupation).

### Experiment 2. SFN Impacts on Germline Tissues

**Fig. 4:**
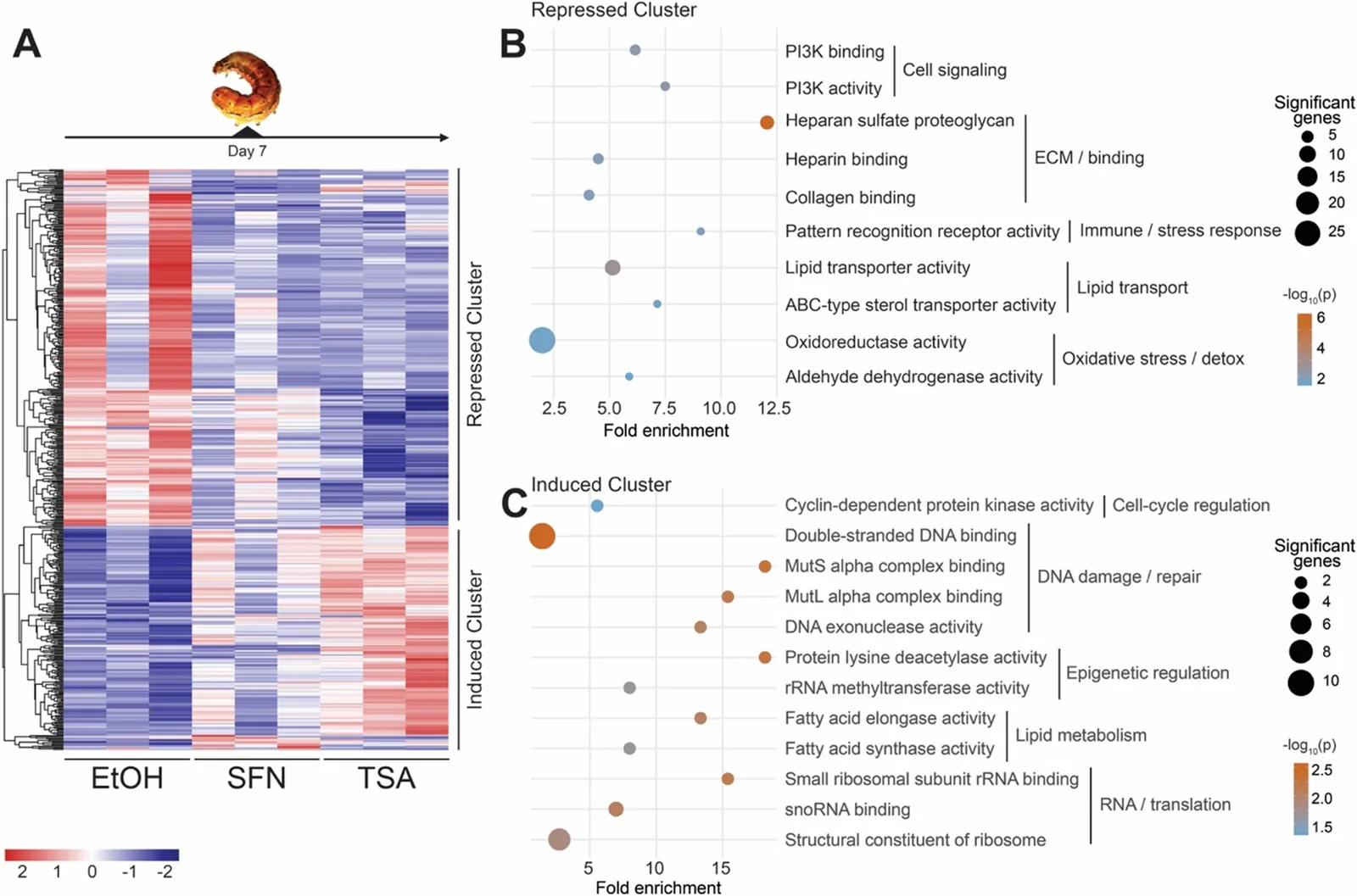
SFN disrupts gene expression in ovaries in *S. exigua* after 7 days. **A** Hierarchical clustering of 546 genes significantly differentially expressed in response to SFN or TSA. Data are row-scaled and variance-stabilizing transformation (VST) -normalized where red indicates higher expression and blue indicates lower expression relative to each gene’s mean expression across all samples. Clustering was performed using the Pearson correlation and complete linkage. **B & C** Molecular Function (MF) Gene Ontology (GO) enrichment for 335 repressed genes (**B**) and 211 induced genes (**C**). Enriched GO categories were manually curated into representative biological themes based on statistical significance, fold enrichment, recurrence among related terms, and biological interpretability. Point size indicates the number of genes associated with each term, and color represents statistical significance. Complete enrichment results are available in Supplemental Tables S13-S16

**Fig. 5:**
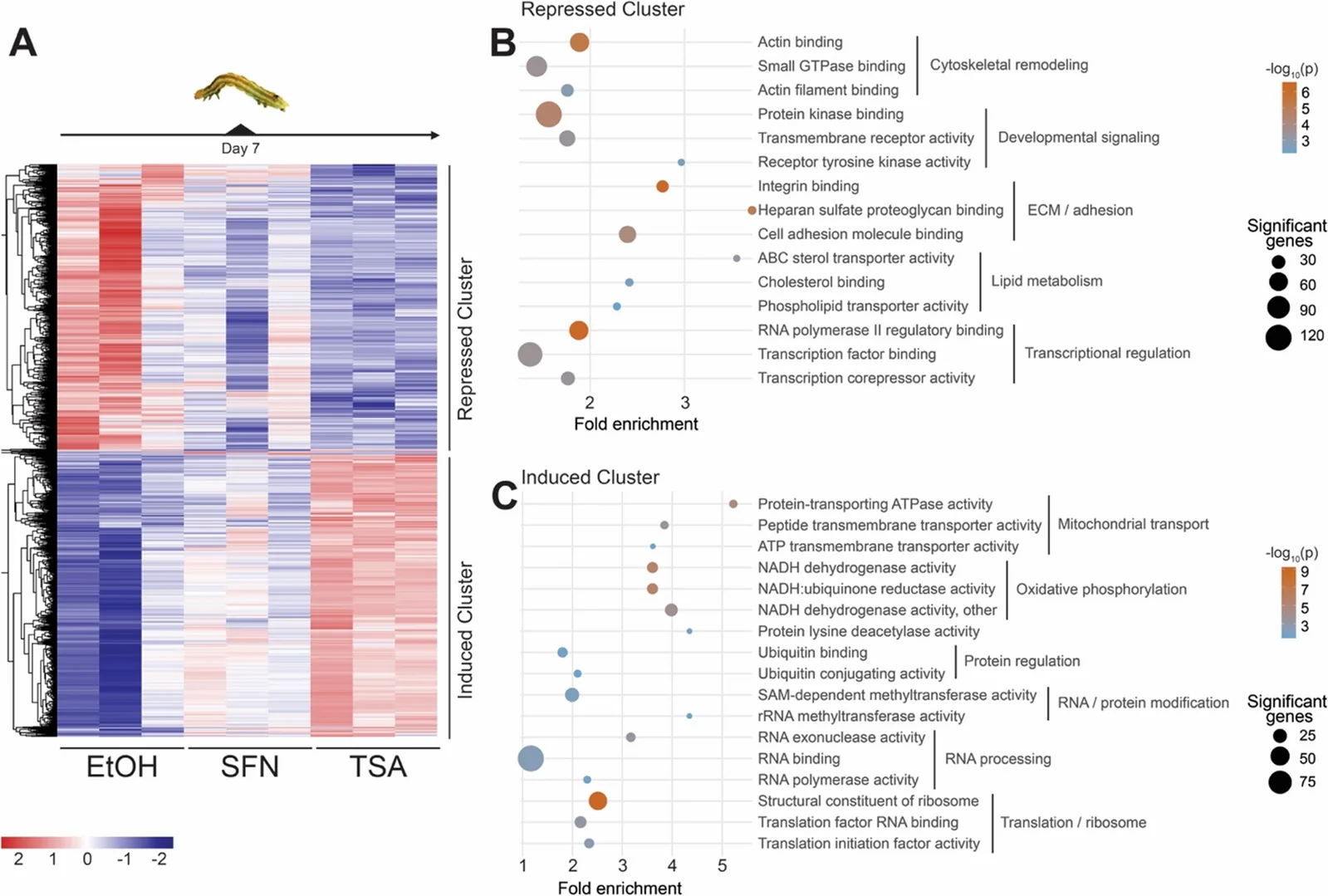
SFN disrupts gene expression in ovaries in *T. ni* after 7 days. **A** Hierarchical clustering of 3,597 genes significantly differentially expressed in response to SFN or TSA. Data are row-scaled and variance-stabilizing transformation (VST) -normalized where red indicates higher expression and blue indicates lower expression relative to each gene’s mean expression across all samples. Clustering was performed using the Pearson correlation and complete linkage. **B & C** Molecular Function (MF) Gene Ontology (GO) enrichment for 1789 repressed genes **(B)** and 1808 induced genes **(C)**. Enriched GO categories were manually curated into representative biological themes based on statistical significance, fold enrichment, recurrence among related terms, and biological interpretability. Point size indicates the number of genes associated with each term, and color represents statistical significance. Complete enrichment results are available in Supplemental Tables S17-S20

SFN also disrupted gene expression in the ovaries of female *S. exigua* (Fig. 4, Fig. S2) and *T. ni* (Fig. 5, Fig. S2). As before, we focused on genes actively expressed during larval development, excluding those with low counts. In the ovaries of larvae consuming SFN or TSA we detected altered expression for 546 genes in *S. exigua* and 3,597 genes in *T. ni*. While exploring these data we found that (a) SFN and TSA have similar effects on ovary tissues, as was noted previously for fat body tissues (see Fig. 5 from Somers et al. 2023) and (b) the difference in the number of differentially expressed genes between the species is due mostly to a difference in statistical power that resulted from the variation between replicates (Supplemental Fig. S2). However, there were a few species-specific differences worth noting. For example, while both species responded to SFN with the upregulation of genes associated with cell cycle regulation, DNA repair, RNA processing, and translation, only *T. ni* exhibited upregulation of genes associated with mitochondrial transport and oxidative phosphorylation (Figs. 4B and C and 5B and C, and Supplemental Tables S13-20). This result is interesting since it is the opposite of the response we observe in *S. exigua* fat body tissues where genes associated with these energy-production pathways are consistently downregulated by SFN. This may be related to this species’ enhanced ability to detoxify products of the mustard oil bomb reactions (Textor and Gershenzon 2009; Winde and Wittstock 2011; Schramm et al. 2012). These species-specific differences are interesting but do not obscure the key point: that SFN consumed in the diet impacted gene expression in germline tissues, in both species.

### Experiment 3. Impacts of SFN Across Generations

**Fig. 6:**
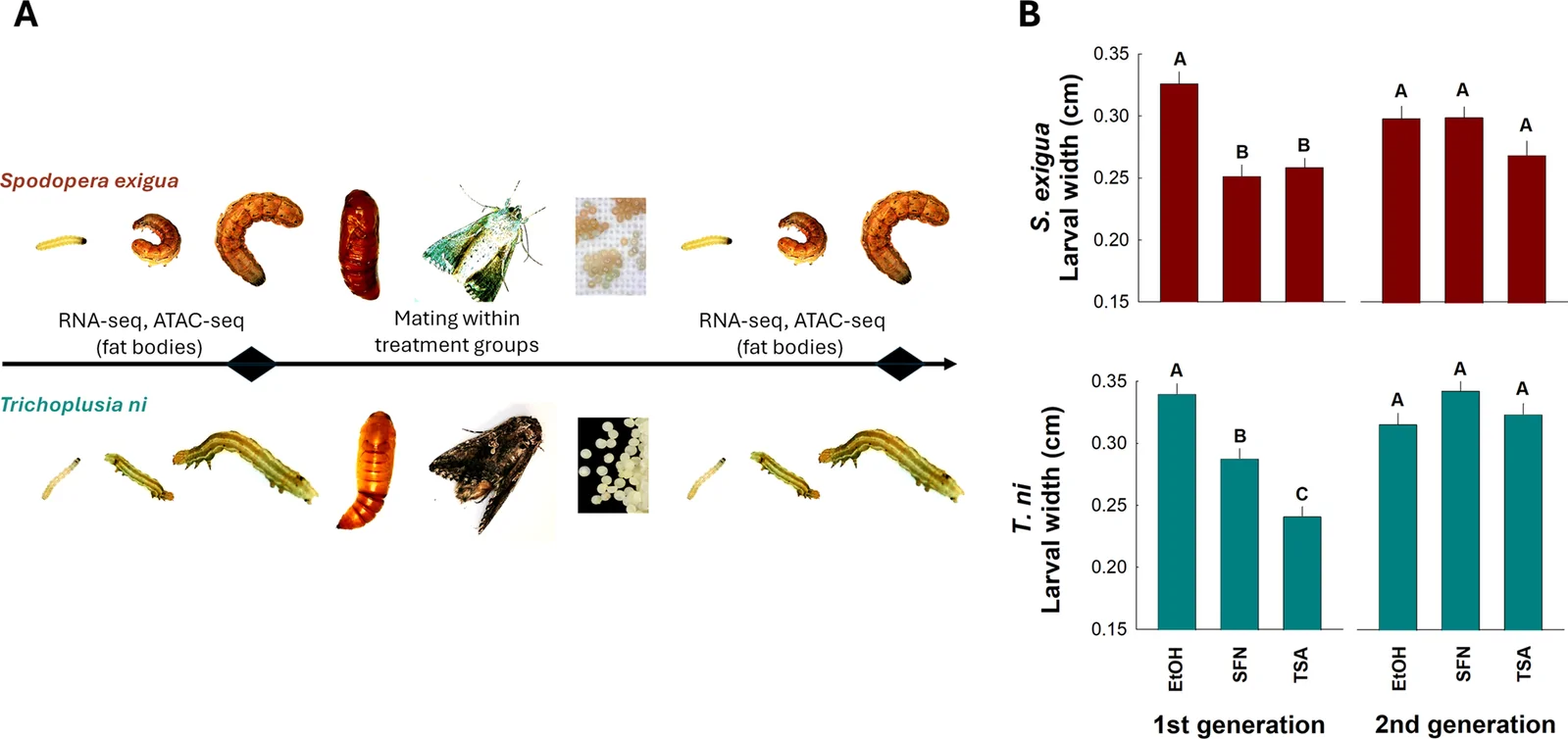
Design of experiment 3. **A** For both *S. exigua* and *T. ni*, first instar larvae in individual wells consumed sulforaphane (SFN) or the pharmaceutical HDAC inhibitor trichostatin A (TSA) in a standard artificial diet (AD). Larvae in negative control groups consumed AD treated only with the carrier solvent (ethanol). EtOH was evaporated from AD prior to feeding. Fat body and ovary tissues were isolated from a subset of larvae for gene expression analyses by RNA-seq and ATAC-seq on Day 7. Remaining larvae were allowed to develop normally and mate within treatment groups to generate second generation larvae, from which tissues were collected on Day 7. **B** Size of first-generation larvae which consumed SFN or TSA and of second-generation larvae which were never exposed to these HDAC inhibitors. Developmental delays were evident in the parental generation (generation 1), which consumed SFN or TSA, but not in their offspring (generation 2)

We tested for intergenerational effects in *S. exigua*, the species that was most responsive to SFN in our previous experiments (Fig. 6).

**Fig. 7:**
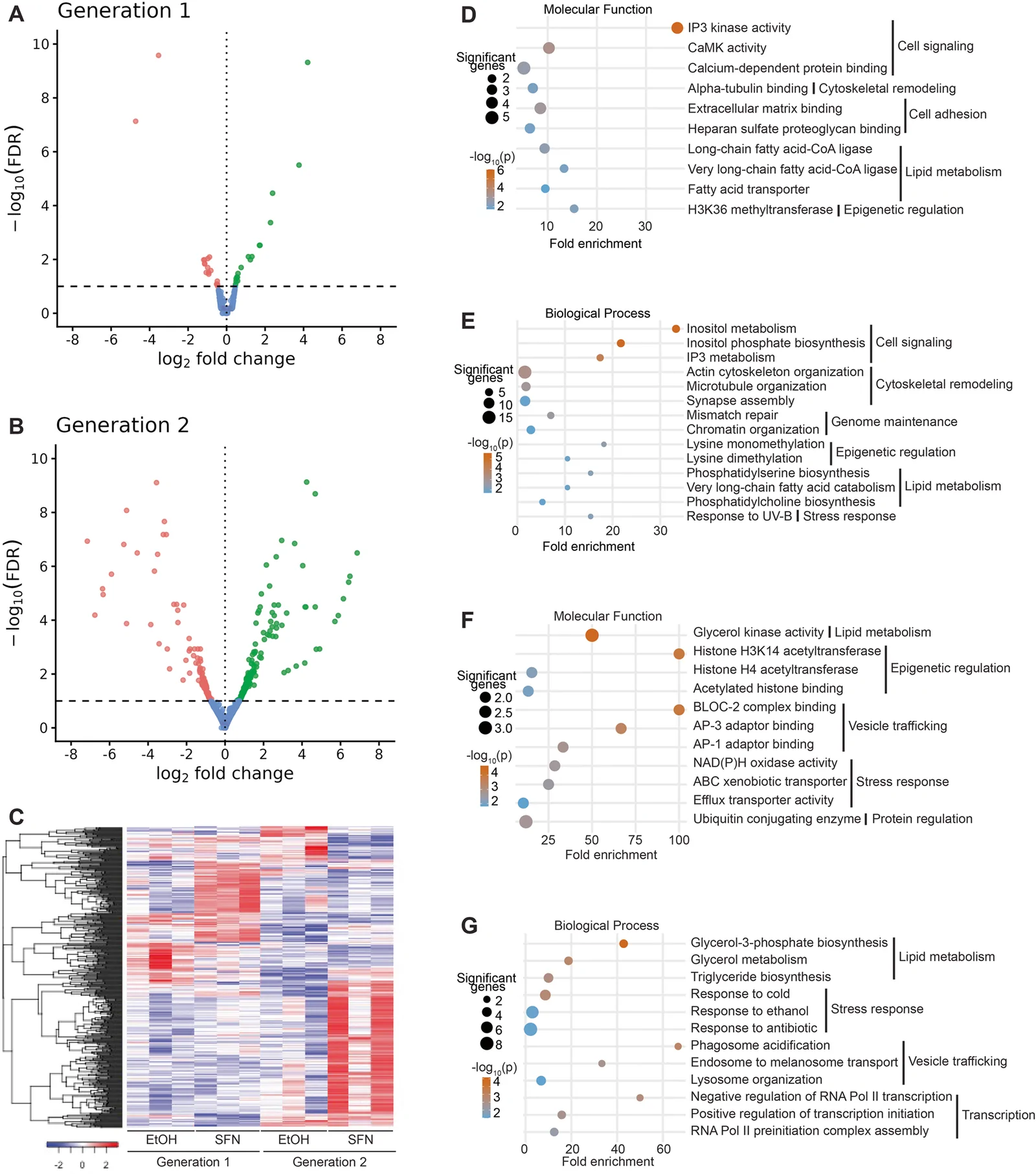
Changes in chromatin accessibility in larvae consuming SFN (Generation 1) and offspring that were never exposed to this substance (Generation 2). **A & B** Volcano plots with points indicating differential accessible regions (DARs) resulting from parental exposure to dietary SFN. The vertical axis indicates the statistical significance of the observed difference. Regions of significantly increased (green) and decreased (orange) chromatin accessibility are plotted above the horizontal dotted line, which represents the cutoff for statistical significance. Regions of chromatin unaffected by paternal SFN exposure (blue) are plotted below this line. The magnitude and direction of the change in chromatin accessibility is indicated on the horizontal axis. ATAC-seq analyses identified 26 DARs (13 with increased accessibility vs. 13 with decreased accessibility) in larvae consuming SFN (**A**) and 271 DARs (181 increased accessibility vs. 91 with decreased accessibility) in offspring which did not consume this substance (**B**). **C** Heatmap showing differential accessibility in regions of key genes in both generations triggered by parental exposure to SFN, compared to the EtOH control group. Gene ontology enrichment data for offspring, including molecular functions and biological processes, are illustrated for regions of increased chromatin accessibility (**D **& **E**) and decreased chromatin accessibility (**F **& **G**). Complete enrichment results are available in Supplementary Tables S22-S25

First, we explored the impacts of SFN on chromatin accessibility in larvae which consumed SFN, and their offspring which did not. ATAC-seq analyses of fat body tissues collected on Day 7 yielded a per-sample average of 66,340,713 uniquely mapped paired-end reads, which corresponds to 73.6–78.2% uniquely mapped reads and FRiP scores ranging from 0.37 to 0.48 (average = 0.42). The dataset had high sequencing quality and strong alignment rates (Supplemental Table 21) and the consensus peak set generated 50,107 total peaks. Libraries exhibited good nucleosomal fragment periodicity, high transcription start site enrichment, and strong replicate concordance (Supplemental Fig. S3). The results indicated that parental exposure to SFN altered chromatin accessibility in both generations (Fig. 7). In first-generation larvae which fed upon SFN we identified 26 differential accessible regions (DARs) where this natural HDAC inhibitor altered chromatin accessibility. Half of these DARs exhibited increased openness, while half were found to be less accessible. In offspring, ATAC-seq analyses revealed a stronger response. In the offspring of *S. exigua* which fed upon an SFN-rich diet we identified 271 DARs – 181 that were more open and 91 that were more closed, compared to offspring from parents in the control group. These data demonstrate that SFN triggers changes in chromatin accessibility in both parents consuming this substance and their offspring which did not. We identified the putative molecular functions and biological processes of genes located in these DARs (Fig. 7D-G, Supplemental Tables 22-25). There were some interesting differences in the SFN-induced chromatin accessibility changes (Supplemental Fig. S4). For example, in parents, the consumption of SFN resulted in regions of altered chromatin accessibility mostly at distal regulatory elements. Their offspring, in contrast, exhibited ten times the number of differentially expressed regions which occurred mostly near genes, in promoters and introns. In short, the accessibility of chromatin was impacted in parents that consumed SFN and offspring which did not, but not in exactly the same ways.

**Fig. 8:**
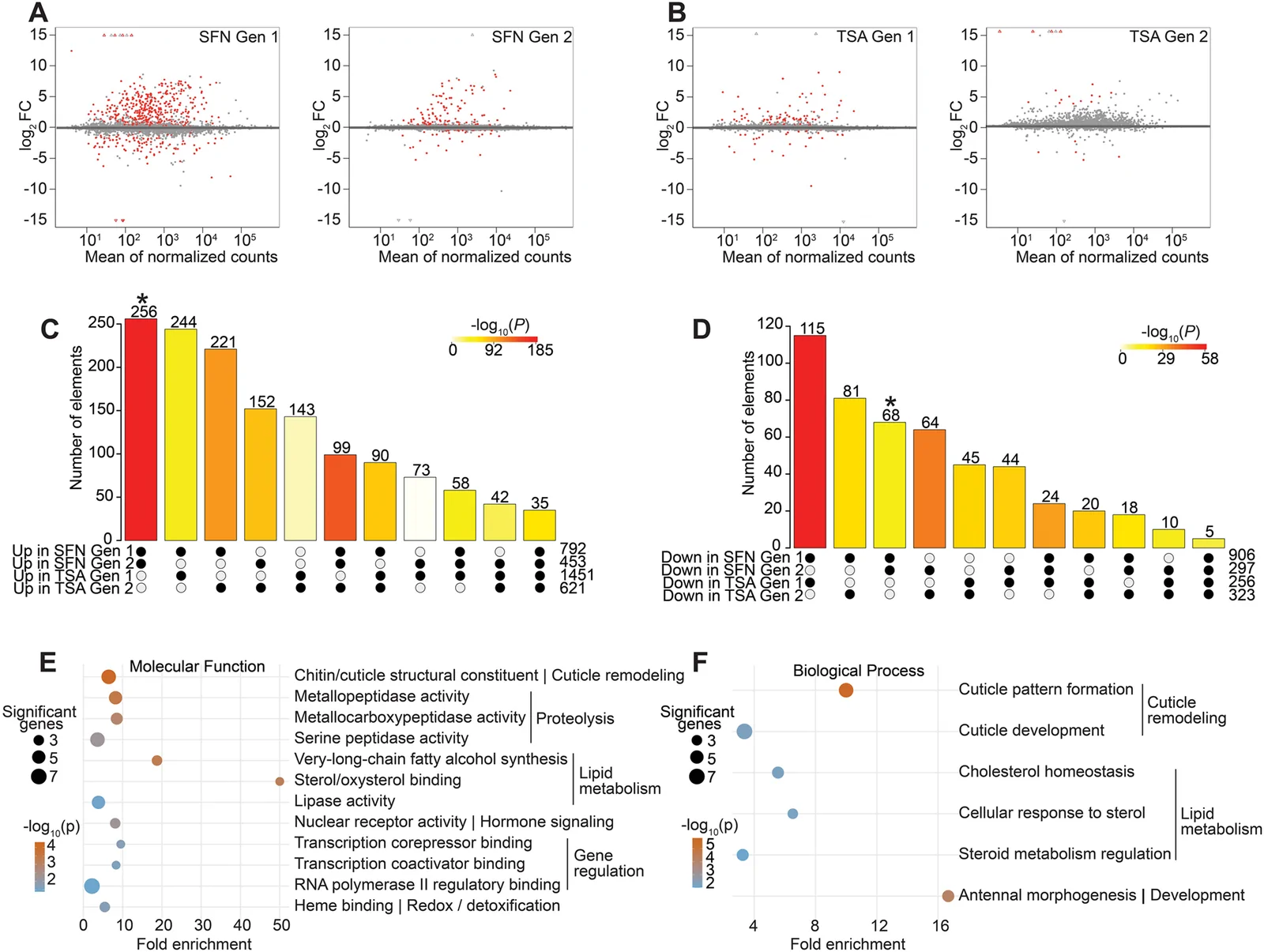
Results of experiment 3 highlighting the intergenerational responses of *S. exigua* to parental SFN exposure. **A & B** MA plots (mean expression versus log₂ fold-change) showing differential expression following treatment with SFN (**A**) or TSA (**B**) over two generations. Significantly differentially expressed genes are indicated as red dots (*p* < 0.05), while non-significant genes are shown in gray. After SFN treatment, 256 genes whose expression patterns are maintained in the second generation were identified as potential epialleles. **C & D.** Intersection plots of the overlap between genes significantly differentially upregulated (**C**) or downregulated (**D**) in response to SFN or TSA treatment across two generations. The total number of differentially expressed genes within each set (*p* < 0.1) is indicated on the bottom right; vertical bars represent the number of genes shared among each intersection indicated by filled black circles. The significance of the overlap was calculated with a multivariate hypergeometric model and is indicated by the color of the vertical bars. Bars representing potential epialleles are indicated with an asterisk. **E & F** Molecular Function (**E**) and Biological Process (**F**) Gene Ontology (GO) enrichment for 256 potential induced epialleles. **G & H** Molecular Function (**G**) and Biological Process (**H**) Gene Ontology (GO) enrichment for 68 potential repressed epialleles. Enriched GO categories were manually curated into representative biological themes based on statistical significance, fold enrichment, recurrence among related terms, and biological interpretability. Point size indicates the number of genes associated with each term, and color represents statistical significance. Complete GO enrichment results are provided in Supplementary Tables S26-S29

We also observed persistent differences in gene expression across the generations in this species (Fig. 8, Supplemental Fig. S5). Specifically, RNA-seq analyses indicated that the expression of 324 genes remained altered in offspring of SFN exposed parents (Fig. 8C, D). This represents approximately 20% of the genes originally affected by SFN in parents. Gene ontology analyses indicated that these genes which remained induced in next generation larvae were associated with processes such as cuticle remodeling, lipid metabolism, and neural development (Fig. 8E, F). Those that remained repressed in offspring were associated with sulfur metabolism, redox reactions, and stress signaling (Fig. 8G, H). The full list of molecular function and biological process enrichment assignments for the putative epialleles are available in Supplemental Tables 26-29. We noted that in *S. exigua* the intergenerational effect of SFN was even greater than that of the pharmaceutical HDAC inhibitor TSA, i.e., more genes “remembered” the impacts of SFN than TSA (Fig. 8C, D).

We did not expect to observe an intergenerational effect in *T. ni* given that the response of this species to SFN was either weak or non-existent in previous experiments. However, we did test for persistent changes in gene expression in *T. ni* offspring in two separate experiments, to be thorough. As expected, there was no evidence for an intergenerational effect in this species, in either experiment (Supplemental Figs. S6 and S7).

We were interested in the location of the SFN-responsive genes. We anticipated that they would occur in regions of chromatin that were affected by this substance. However, several factors make it difficult to test this assumption by directly comparing the RNA-seq and ATAC-seq datasets. First, the RNA-seq and ATAC-seq analyses were not conducted on the same individuals; instead, they were conducted on different subsets of specimens randomly sampled from each group of larvae from the same experiment. Second, DARs identified by ATAC-seq contained combinations of promoter regions, exons, and introns which may be associated with multiple genes. Some of these genes were associated with DARs exhibiting increased accessibility in one region and decreased accessibility in another. Finally, most DARs contained multiple genes (mean 4.46 peaks / gene). However, we did evaluate this in another, though less powerful way. We compared the molecular functions and biological processes assigned to genes in these two datasets, e.g., by comparing the putative functions of genes identified as differentially-expressed by RNA-seq and those occurring in DARs as identified by ATAC-seq. There was indeed significant concordance, which can be observed by comparing the gene function assignments in Figs. 7 and 8, for example.

## Discussion

We found that the consumption of SFN alters chromatin accessibility, disrupts the expression of 1800 genes, and delays development in the herbivore *S. exigua*. In this species, SFN suppressed genes related to critical energy production pathways, including pyruvate metabolism, oxidative phosphorylation, the TCA cycle, glycolysis, and gluconeogenesis, which helps to explain how this substance slows larval development by up to 50%. These impacts are likely due to the SFN’s activity as a natural inhibitor of HDAC enzymes produced in the mustard oil bomb reactions; however, SFN has multiple molecular targets, including additional chromatin-modifying enzymes and pathways, which may also contribute to this response (Vanduchova et al. 2019; Santín-Márquez et al. 2019; Coutinho et al. 2023).

SFN also impacted germline tissues; specifically, it altered the expression of hundreds of genes in ovaries, in both species. These SFN-induced changes were associated with broad changes in chromatin accessibility. Finally, we demonstrated that changes in both chromatin accessibility and gene expression can persist in offspring which were never exposed to SFN. This suggests that cruciferous plants can use SFN to disrupt the epigenetic systems of herbivores, i.e., to reshape Waddington’s epigenetic landscape from underneath. These data also confirm the presence of an intergenerational effect, which may or may not be categorized as transgenerational epigenetic inheritance, raising more questions about how exposure to this product of the mustard oil bomb could impact future generations of herbivores.

### Experiment 1. SFN Delays Normal Waves of Gene Expression During Development

Dietary SFN had dramatic effects on gene expression on both species, but especially in *S. exigua*, as we documented previously (Somers et al. 2023). In these experiments and those reported previously we observed that ~ 1800 genes, or about 10% of the transcriptome of these insects, were altered by the consumption of SFN. These numbers and proportions of effected genes are remarkably similar to those observed in previous studies, which are predominately studies of cancer cells in mammalian tissues being treated by SFN or similar HDAC inhibitors (Tortorella et al. 2015; Li and Sun 2019). We also observed both up- and down-regulation of genes following exposure to SFN, which is a common effect of HDAC inhibitors such as SFN (Beaver et al. 2014; Chueh et al. 2015; Li et al. 2016).

By conducting RNA-seq analyses at two-day intervals, rather than at a single endpoint, we were able to detect disruptions in the usual patterns of gene expression occurring during normal larval development following the consumption of SFN. We observed delays in the expression of key developmental genes in *S. exigua* and, to a lesser extent, in *T. ni*. Genes associated with metabolic activity and biosynthesis, which typically increase during larval development, were suppressed by SFN treatment. In particular, we noted a dramatic downregulation of genes associated with energy production pathways in *S. exigua*, as we did previously (Somers et al. 2023). This likely explains the dramatic reduction of growth we observe in this species. In addition, we observed that genes required for cuticle formation and tissue remodeling were suppressed in SFN-treated larvae, suggesting altered development. We observed the upregulation of genes involved in signaling and developmental regulation. We also examined the expression of genes associated with key detoxification processes, including those known to assist in the detoxification of SFN. In general, *T. ni* exhibited markedly higher constitutive expression of key genes coding for UDP-glucoronosyl and UDP-glucosyl transferases, cytochrome P450s, ABC transporters, superoxide dismutase, and glutathione S-transferases, even in the absence of SFN exposure. Both species exhibited SFN-induced expression of genes associated with detoxification processes. *S. exigua* responded by inducing the expression of glutathione transferases, glutathione synthetases, ABC transporters, and P450 enzymes and *T. ni* responded mostly with the induced expression of glutathione transferase and P450 genes. However, these induced changes were modest in comparison to the constitutive differences we observed between these species.

Together, these results indicate that SFN works quickly – within days – to alter the expression of approximately 10% of the genes typically expressed in the larval stage. Interestingly, while this was consistently associated with slower development it did not cause mortality in these laboratory experiments. In our experience larval mortality only occurred with much higher levels of SFN, well above those occurring in nature. This is consistent with the observations of others who report that the mustard oil bomb defenses are usually associated with the reduced performance of herbivores, but do not directly cause mortality (Textor and Gershenzon 2009; Winde and Wittstock 2011).

### Experiment 2. SFN Alters Gene Expression in Germline Tissues

Previously, we focused on gene expression changes in fat body tissues. However, when considering the impacts of SFN on future generations it is useful to probe for effects on germline tissues (Perez and Lehner 2019; Fitz-James and Cavalli 2022). Thus, we expanded the scope of our work to examine gene expression changes in ovaries isolated from female larvae. We observed dramatic gene expression changes in these germline tissues as well, in both species (Figs. 3 and 4). Some of these gene expression patterns were familiar. For example, just as in fat body tissues, ovaries exhibited upregulation of genes associated with DNA and RNA processing and the synthesis of ribosomes and proteins. However, there were a few interesting differences. For instance, we observed a surprising *increase* in genes associated with OXPHOS and mitochondria transport in the ovaries of *T. ni*, following the consumption of SFN. This is a sharp contrast to the strong downregulation of genes associated with energy production pathways we observed in *S. exigua* fat body tissues. This may help *T. ni* to mitigate the effects of dietary SFN. In any case, despite these curious species-specific differences, these RNA-seq analyses confirm that dietary SFN can alter gene expression in germline tissues. We are unaware of previous studies showing that this compound impacts germline tissues in animals, though cancer stem cells are known to be affected (Coutinho et al. 2023).

### Experiment 3. Intergenerational Effects of SFN

In these multigenerational experiments we again observed that dietary SFN alters expression of over 1000 genes in larvae of both species, as reported previously (Somers et al. 2023). Here we confirmed, for the first time, that offspring also exhibited some of the same SFN-induced gene expression patterns as did their parents, even though they never consumed this substance (Fig. 7). For example, in *S. exigua* we found 324 genes (256 upregulated and 68 downregulated) for which expression was altered both in parents which consumed SFN and in offspring which did not. In other words, approximately 20% of the genes impacted by the consumption of SFN in parents remained altered, in the same direction, in their offspring. We tentatively suggest that these genes are SFN-sensitive epialleles, sometimes called “remembering” genes, that are more prone to epigenetic programming across generations.

Because first generation *T. ni* larvae usually exhibited weaker responses to SFN we did not expect to observe intergenerational effects in the second-generation larvae. Nonetheless, we tested this in a series of multigenerational experiments (Supplemental Figs. S6, S7). The results of RNA-seq analyses for two replicate experiments confirmed the absence of significant SFN-induced effects in *T. ni* offspring. From these experiments, and others, it is clear that both first- and second-generation larvae of this species are less affected by ecologically-relevant levels of SFN. It is possible that much of the SFN ingested by *T. ni* was detoxified before reaching the tissues we examined. This species is known to possess a robust glutathione-based detoxification system, capable of conjugating electrophilic isothiocyanates and metabolizing them via the mercapturic acid pathway or excreting them, to reduce the effectiveness of the mustard oil bomb defenses of cruciferous plants (Textor and Gershenzon 2009; Winde and Wittstock 2011; Schramm et al. 2012).

In *S. exigua* the consumption of SFN alters the accessibility of chromatin. ATAC-seq analyses identified 26 differentially accessible regions (DARs) directly affected by SFN. This included 13 regions of chromatin that became more accessible and 13 regions that became less accessible (Fig. 6). Changes in chromatin accessibility were also evident in offspring, triggered by parental exposure to SFN. In next generation larvae which never consumed SFN, ATAC-seq analyses highlighted 271 DARs (181 regions of increased accessibility, and 91 regions of reduced accessibility). We did not conduct ATAC-seq analyses of chromatin accessibility in *T. ni*, since we had not observed persistent changes in gene expression across generations in this species.

One of the most striking and consistent effects of SFN on first generation *S. exigua* larvae, observed here and previously, is the downregulation of genes associated with energy metabolism pathways such as pyruvate metabolism, oxidative phosphorylation, the TCA cycle, glycolysis, and gluconeogenesis. Interestingly, we did *not* observe this in offspring (Fig. 7E, F). As a result, it seems logical that we did not detect reduced growth, delays in development, or changes in morphology in offspring from affected *S. exigua* parents. In short, these particular effects of SFN on development seem to be confined to the parents who consumed the substance.

Together, these data demonstrate that SFN alters both chromatin accessibility and gene expression in first generation *S. exigua* larvae which consumed SFN and in their offspring, which did not. This raises several additional questions, addressed below.

### Weighing the Evidence for an Intergenerational Effect

In these experiments it became clear that the consumption of SFN, a known HDAC inhibitor, by *S. exigua* can alter both the accessibility of chromatin and the expression of genes in offspring that did not consume this secondary metabolite. This demonstrates a general intergenerational effect. The data also provide some evidence related to potential for transgenerational epigenetic inheritance, which is worth discussing.

Transgenerational epigenetic inheritance (TEI) is a specific type of intergeneration effect (Bossdorf et al. 2008; Richards et al. 2010; Duncan et al. 2014; Auge et al. 2017; Hu and Barrett 2017; Perez and Lehner 2019; Ramos-Cruz et al. 2021; Fitz-James and Cavalli 2022; Fallet et al. 2023; Bird 2024). However, there is a high bar for such a classification, as summarized by Khatib et al. (2024): (1) an induced phenotypic or transcriptional response, (2) association with epigenetic modifications, (3) persistence of these modifications or their effects in the next generation, (4) recurrence of the phenotype or gene expression changes in offspring, (5) involvement of germline cells in transmission, and (6) exclusion of alternative mechanisms such as direct exposure, maternal provisioning, or microbiome transfer. It is worth noting that, to date, very few published studies have satisfied these criteria in any system (Khatib et al. 2024). For example, in a meta-analysis of 80 studies Khatib et al. found that none met all six of the criteria, underscoring the difficulty of conclusively demonstrating this specific type of intergenerational effect.

Here we observed direct effects of SFN on first generation larvae, the involvement of epigenetic systems, the persistence of epigenetic modifications, recurrence of gene expression changes in offspring, and the involvement of germline cells. However, this evidence is incomplete. We did not directly measure changes in histone acetylation. We did not test chromatin accessibility or gene expression in germ cells (i.e., eggs or sperm cells), only in germline tissues (e.g., ovaries). In addition, our experiments were not designed to eliminate alternative mechanisms of intergenerational transfer, such as maternal provisioning differences, or to test for the presence of trace amounts of SFN that could have been carried in or on gametes or deposited egg masses. Finally, we did not test for changes in the gut microbial biomes of these insects. The most common approach to eliminating these alternative explanations would be to follow epigenetic changes through at least three generations. This was not feasible in our experiments but could be tested in the future. As a result, we do not claim to have demonstrated TEI – which would require additional experiments to elucidate the epigenetic mechanisms. Rather, the present data demonstrate that SFN can have a general intergenerational effect which involves epigenetic systems and results in changes in gene expression across generations.

### Ecological Implications

Dietary SFN had clear effects on *S. exigua*. The consumption of SFN at natural concentrations inhibits HDAC enzymes, alters the expression of ~ 1500–1800 genes in multiple tissues, and slows development by 2–7 days (Somers et al. 2023). We can elicit more striking effects in *S. exigua*, and overwhelm the apparent resistance of *T. ni*, using supernatural concentrations of SFN or by using more powerful pharmaceutical HDAC inhibitors. Interestingly, in a decade of examining the response of lepidopterans to ecologically-relevant concentrations of SFN we have never observed increased mortality nor morphological or behavioral changes. In our no-choice feeding experiments, the most obvious effect of SFN is slower development. In nature, this can be ecologically important. For example, a modest delay in larval development by 2–4 days per generation would likely reduce the number of generations of grazing larvae in a growing season, e.g., from five to four in the mid-Atlantic region of the United States. In addition, slower developing grazers may suffer increased mortality from predators and parasitoids during an extended *“window of vulnerability*,*”* as suggested by the “slow growth-high mortality hypothesis.” (Benrey and Denno 1997; Williams 1999; Uesugi 2015; Holmes et al. 2023). Coincidently, this hypothesis originated 30 years ago from a study of caterpillars feeding on cruciferous host plants, including those producing SFN. Both of these potential effects of SFN consumption – the reduction in the number of generations per season and decreases in survival – require testing in the field. It is possible that in a natural setting herbivores might avoid these effects by choosing to feed on other plants nearby. We might expect this type of defense to be most impactful for herbivores where there are multiple generations per season, where offspring remain associated with the same host, and where parental exposure predicts offspring exposure (Ramos-Cruz et al. 2021; Tando and Matsui 2023).

We have also considered that SFN may disrupt the phenotypic plasticity of herbivores – that is, not to alter their survival, morphology, or behavior under ideal conditions but to limit their ability to respond to change. For instance, epigenetic systems (including those disrupted by HDAC inhibitors) can influence phenotypic plasticity in response to heat and cold stress (Mogilicherla and Roy 2023; Mukherjee and Dobrindt 2024; Zetzsche and Fallet 2024). In our experiments, for instance, we have observed that SFN and related HDAC inhibitors reduce the expression of heat shock proteins and suppress the activity of immune system enzymes in *S. exigua.* Thus, future experiments combining exposure to SFN with combinations of biotic and abiotic stressors could be valuable.

### Conclusion

SFN, a product of the mustard oil bomb reactions in cruciferous plants, can disrupt the epigenetic systems of *S. exigua* when consumed at natural concentrations. It can inhibit HDAC enzyme activity, change chromatin accessibility, alter gene expression, and delay larval development. Some of the SFN-induced gene expression changes persist in second generation larvae that did not consume SFN. We observed that certain genes, which we consider to be putative epialleles, may be more prone than others to “remembering” SFN exposure across generations. Together, these data demonstrate that SFN can have intergenerational effects. Our results satisfy some, but not all, of the criteria for transgenerational epigenetic inheritance. However, regardless of the mechanism, it is evident that SFN from the mustard oil bomb reactions can sabotage the epigenetic control systems of grazing insects across generations. Rather than simply altering trajectories across Waddington’s epigenetic landscape, SFN appears capable of disrupting the molecular machinery that shapes the landscape itself, functioning as an epigenetic weapon against grazing insects.

We note that plants produce numerous secondary metabolites with known epigenetic activity, including many HDAC inhibitors that affect both insects and mammals. In addition to the cruciferous plants, these include garlic, turmeric, mustard seeds, grapes, tea, ginger, basil, and various fruits (Seidel et al. 2012; Bassett and Barnett 2014; Losson et al. 2016; Singh et al. 2018; Evans and Ferguson 2018). Our results suggest that HDAC inhibitors from these plants should be investigated not only as toxins or feeding deterrents, but also as potential epigenetic disruptors.

## Supplementary Information

Supplementary Material 1.

## Data Availability

For the RNA-seq analyses, all raw sequence reads were deposited in the NIH Sequence Read Archive (SRA) under BioProject numbers PRJNA1497926 (*S. exigua*) and PRJNA1498032 (*T. ni*). For ATAC-seq analyses, all raw sequence reads were deposited in the NIH Sequence Read Archive (SRA) under BioProject number PRJNA1498575.
