# Supplementary material for: Sulforaphane, A Mustard Oil Bomb Product, Reprograms Herbivore Gene Expression and Alters Chromatin Structure Across Generations
Source: J Chem Ecol. 2026 Oct 29;52(6):100. doi: 10.1007/s10886-026-01771-x (PMC13723448; doi:10.1007/s10886-026-01771-x)
Supplement: Supplementary file 1 [file 10886_2026_1771_MOESM1_ESM.pdf]

SUPPLEMENTAL TEXT, FIGURES, AND TABLES FOR:

SULFORAPHANE, A MUSTARD OIL BOMB PRODUCT, REPROGRAMS HERBIVORE GENE EXPRESSION BY ALTERING CHROMATIN STRUCTURE ACROSS GENERATIONS

THOMAS M. ARNOLD[1]*

Dickinson College, Department of Biology, Carlisle PA 17013 (USA) arnoldt@dickinson.edu

DANA J. SOMERS[1]

Dickinson College, Department of Biology, Carlisle PA 17013 (USA) somersd@dickinson.edu

DAVID B. KUSHNER

Dickinson College, Department of Biology, Carlisle PA 17013 (USA) kushnerd@dickinson.edu

MARISA ARREOLA

Dickinson College, Department of Biology, Carlisle PA 17013 (USA) arreolamedicine@gmail.com

WHITNEY FINNEY

Dickinson College, Department of Biology, Carlisle PA 17013 (USA) finney.whitney3272@gmail.com

ASHLEY GROFF

Dickinson College, Department of Biology, Carlisle PA 17013 (USA) ashgroff6@gmail.com

ANNIKA HAAGENSEN

Dickinson College, Department of Biology, Carlisle PA 17013 (USA) ahaagensen1@mgh.harvard.edu

MEL MORALES

Dickinson College, Department of Biology, Carlisle PA 17013 (USA) moraleme@dickinson.edu

CHARLIE KIM

Dickinson College, Department of Biology, Carlisle PA 17013 (USA) kbs000201@gmail.com

EMI AYALA-SEKIGUCHI

Dickinson College, Department of Biology, Carlisle PA 17013 (USA) ayalasee@dickinson.edu

MASON ARNOLD

Dickinson College, Department of Biology, Carlisle PA 17013 (USA) masonarnold240@gmail.com

[1] these authors contributed equally to this work.

Table of Contents

Supplemental Methods………………………………………………………………………. 2

Supplemental References……………………………………………………………….… 4

Supplemental Figures…………………………………………………...……………..…… 5

Supplemental Tables S1 – S6, related to Figure 2……………...……………………….……… 14

Supplemental Tables S7 – S12, related to Figure 3……………...…………………….….….. 20

Supplemental Tables S13 – S16, related to Figure 4……………...…………………….……. 26

Supplemental Tables S17 – S20, related to Figure 5……………...…………………….……. 30

Supplemental Tables S21 – S25, related to Figure 7……………...…………………….……. 34

Supplemental Tables S26 – S29, related to Figure 8……………...…………………….……. 39

Supplemental Methods

RNA extraction, library construction, and sequencing. Samples were centrifuged at 10,000 x g for 20 seconds and RNAlater was removed from pelleted tissue. Total RNA was extracted from the tissue twice using Buffer A (50 mM sodium acetate pH 5.2, 10 mM EDTA, 1 % SDS) saturated phenol heated to 65 °C, followed by phenol/chloroform (1:1) extraction. Extracts were ethanol precipitated, resuspended in ddH_2O and ethanol precipitated again. Final pellets were resuspended in ddH_2O to a concentration of 500-1500 ng/ml. RNA concentration was determined using a DS-11 spectrophotometer (DeNovix, Wilmington, Delaware, USA). Total RNA quality was assessed using RNA Nano Chips on a 2100 BioAnalyzer (Agilent, Santa Clara, California, USA) and Qubit RNA High Sensitivity Assay (ThermoFisher, Waltham, Massachusetts, USA), followed by High Sensitivity RNA ScreenTape Analysis (Agilent, Santa Clara, California, USA).

Library construction and sequencing was performed by Admera Health Biopharma Services (South Plainfield, New Jersey, USA). Poly(A) selection was performed using the NEBNext Poly(A) mRNA Magnetic Isolation Module (New England Biolabs, Ipswich, Massachusetts, USA) following the manufacturer's protocols. RNA-seq libraries were generated using the NEBNext Ultra II RNA Library Prep Kit for Illumina (New England Biolabs, Ipswich, Massachusetts, USA) following manufacturer's protocols. Final library quantity was assessed by Qubit 2.0 (ThermoFisher, Waltham, Massachusetts, USA), and quality was assessed by TapeStation D1000 ScreenTape (Agilent, Santa Clara, California, USA). Libraries were standardized to equal molar ratios and then pooled. Paired-end 150-bp reads were generated using the NovaSeq X Plus 10B platform (Illumina, San Diego, California, USA).
All raw sequence reads were deposited in the NIH Sequence Read Archive (SRA) under BioProject numbers PRJNA1497926 (*S. exigua*) and PRJNA1498032 (*T. ni*).

RNA-seq processing and analysis. Reads for all RNA-seq experiments were quality checked with FastQC (v0.11.9) (Andrews) and were processed with Trimmomatic (v0.39) (Bolger et al 2014) using the following parameters: LEADING:5, TRAILING:5, MAXINFO:36:0.2, MINLEN:50. *T. ni* reads were aligned to the *T. ni* Cornell-1 isolate genome (obtained from the *T. ni* Genome Database on 2022-05-25) and *S. exigua* reads were aligned to the *S. exigua* PGI_SPEXI_v6 genome assembly (GCA_902829305.4 obtained from the

NCBI Genome Database on 2022-11-15) (Chen et al. 2019; Simon et al. 2021). Reads were aligned using HISAT2 (v2.2.1) with the default parameters, and alignments were quality checked using QualiMap (v2.3) (Okonechnikov et al. 2016). featureCounts implemented in the Rsubread package (v2.12.2) was used to calculate read counts for each gene using the appropriate species-specific annotations (Liao et al. 2019).

ATAC-seq nuclei extraction, library construction, and sequencing. ATAC-seq was performed by Admera Health Biopharma Services (South Plainfield, New Jersey, USA) as previously described (Corces et al. 2017). Briefly, frozen tissue was thawed in cold homogenization buffer on ice for 5 mins. Tissue dissociation was performed in Nuclei Extraction Buffer (Miltenyi Biotec, Bergisch Gladbach, Germany) with the gentleMACS Tissue Dissociator (Miltenyi Biotec, Bergisch Gladbach, Germany) following the manufacturer's protocol. Nuclei number was assessed by Cellometer Auto 2000 (Nexcelom Bioscience, Massachusetts, USA). 50K to 100K cells were mixed with 1 mL cold ATAC resuspension buffer for 5 mins on ice and centrifuged at 500 RCF in a pre-chilled centrifuge for 10 min. Supernatant was removed and nuclei were resuspended in 50 uL tagmentation reaction buffer by pipetting up and down gently. The reactions were incubated at 37 °C for 30 min in a thermomixer with shaking at 1,000 RPM and cleaned using the MinElute Reaction Cleanup Kit (Qiagen, Hilden, Germany) following the manufacturer's protocol. Tagmented DNA was amplified with barcode primers. Library quality and quantity were assessed with Qubit 2.0 DNA HS Assay (ThermoFisher, Massachusetts, USA), Tapestation High Sensitivity D1000 Assay (Agilent Technologies, California, USA), and QuantStudio ® 5 System (Applied Biosystems, California, USA). Equimolar pooling of libraries was performed based on QC values and sequenced on an Illumina® NovaSeq X Plus 10B (Illumina, California, USA) with a read length configuration of 150-bp for 50 M paired-end reads (25 M in each direction) per sample. All raw sequence reads were deposited in the NIH Sequence Read Archive (SRA) under BioProject number PRJNA1498575.

Supplemental References

Andrews S. FastQC: a quality control tool for high throughput sequence data. Version 0.11.9 [software]. Available from: https://www.bioinformatics.babraham.ac.uk/projects/fastqc.

Bolger AM, Lohse M, Usadel B. (2014) Trimmomatic: a flexible trimmer for Illumina sequence data. Bioinformatics. 30: 2114–2120. https://doi.org/10.1093/bioinformatics/btu170

Chen W, Yang X, Tetreau G, et al (2019) A high-quality chromosome-level genome assembly of a generalist herbivore, *Trichoplusia ni*. Mol Ecol Resour 19:485–496. https://doi.org/10.1111/1755-0998.12966

Corces MR, Trevino AE, Hamilton EG, et al (2017) An improved ATAC-seq protocol reduces background and enables interrogation of frozen tissues. Nat Methods 14:959–962. https://doi.org/10.1038/nmeth.4396

Liao Y, Smyth GK, Shi W (2019) The R package Rsubread is easier, faster, cheaper and better for alignment and quantification of RNA sequencing reads. Nucleic Acids Res 47:e47. https://doi.org/10.1093/nar/gkz114

Okonechnikov K, Conesa A, García-Alcalde F (2016) Qualimap 2: advanced multi-sample quality control for high-throughput sequencing data. Bioinformatics 32:292–294. https://doi.org/10.1093/bioinformatics/btv566

Simon S, Breeschoten T, Jansen HJ, et al (2021) Genome and transcriptome analysis of the beet armyworm *Spodoptera exigua* reveals targets for pest control. G3 (Bethesda) 11:jkab311. https://doi.org/10.1093/g3journal/jkab311

Supplemental Figures and Tables

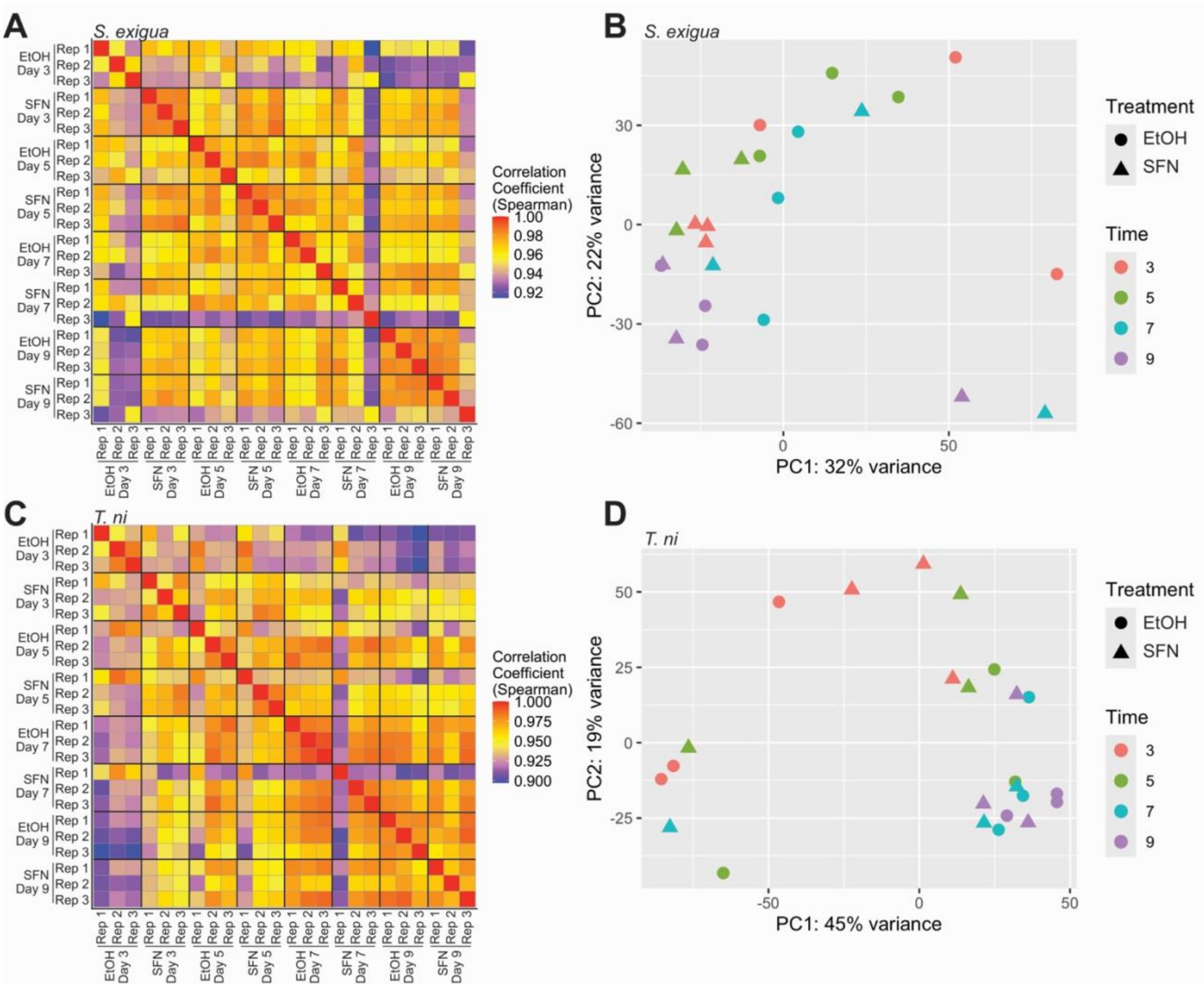

Figure S1, related to Figures 2 and 3. Sample-to-sample relationships among RNA-seq libraries for larval developmental time course experiments. **A, C.** Heatmaps show the pairwise Spearman correlation coefficients calculated from rlog-transformed gene expression values for all RNA-seq samples in *S. exigua* **(A)** and *T. ni* **(C)**. Warmer colors indicate higher correlation between samples. **B, D.** Principal component analysis (PCA) of rlog-transformed gene expression values shows the proportion of total variance explained by each principal component for *S. exigua* **(B)** and *T. ni* **(D)**. Each point represents an individual biological replicate, with colors indicating day of larval development and symbols indicating treatment group. Samples cluster primarily according to the day of larval development.

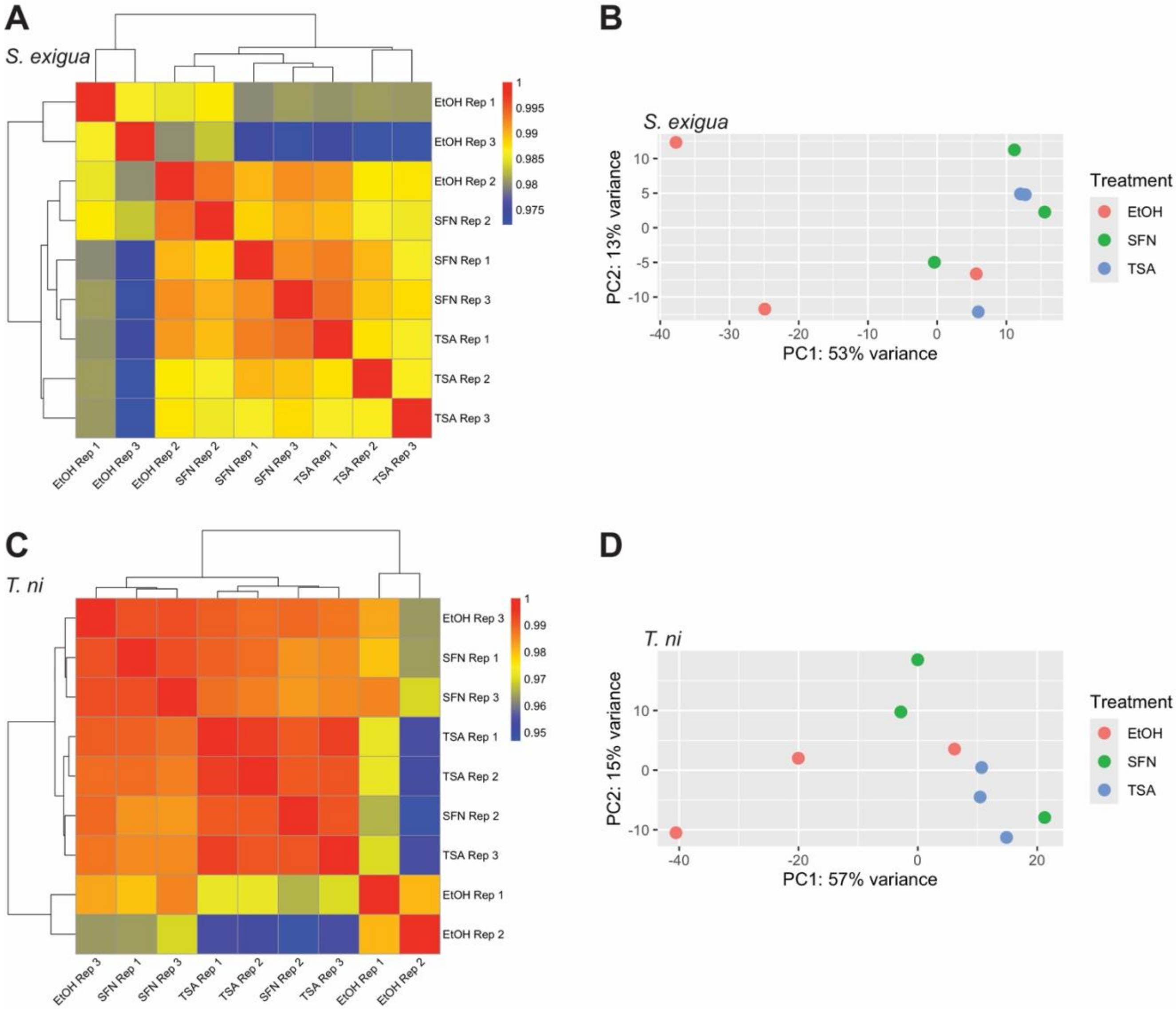

Figure S2, related to Figures 4 and 5. Sample-to-sample relationships among RNA-seq libraries for ovary tissues. **A, C.** Heatmaps show the pairwise Spearman correlation coefficients calculated from rlog-transformed gene expression values for all RNA-seq samples in *S. exigua* **(A)** and *T. ni* **(C)**. Warmer colors indicate higher correlation between samples and dendrograms show hierarchical clustering of samples based on correlation distance. **B, D.** Principal component analysis (PCA) of rlog-transformed gene expression values shows the proportion of total variance explained by each principal component for *S. exigua* **(B)** and *T. ni* **(D)**. Each point represents an individual biological replicate, with colors indicating treatment group. Samples cluster primarily according to treatment.

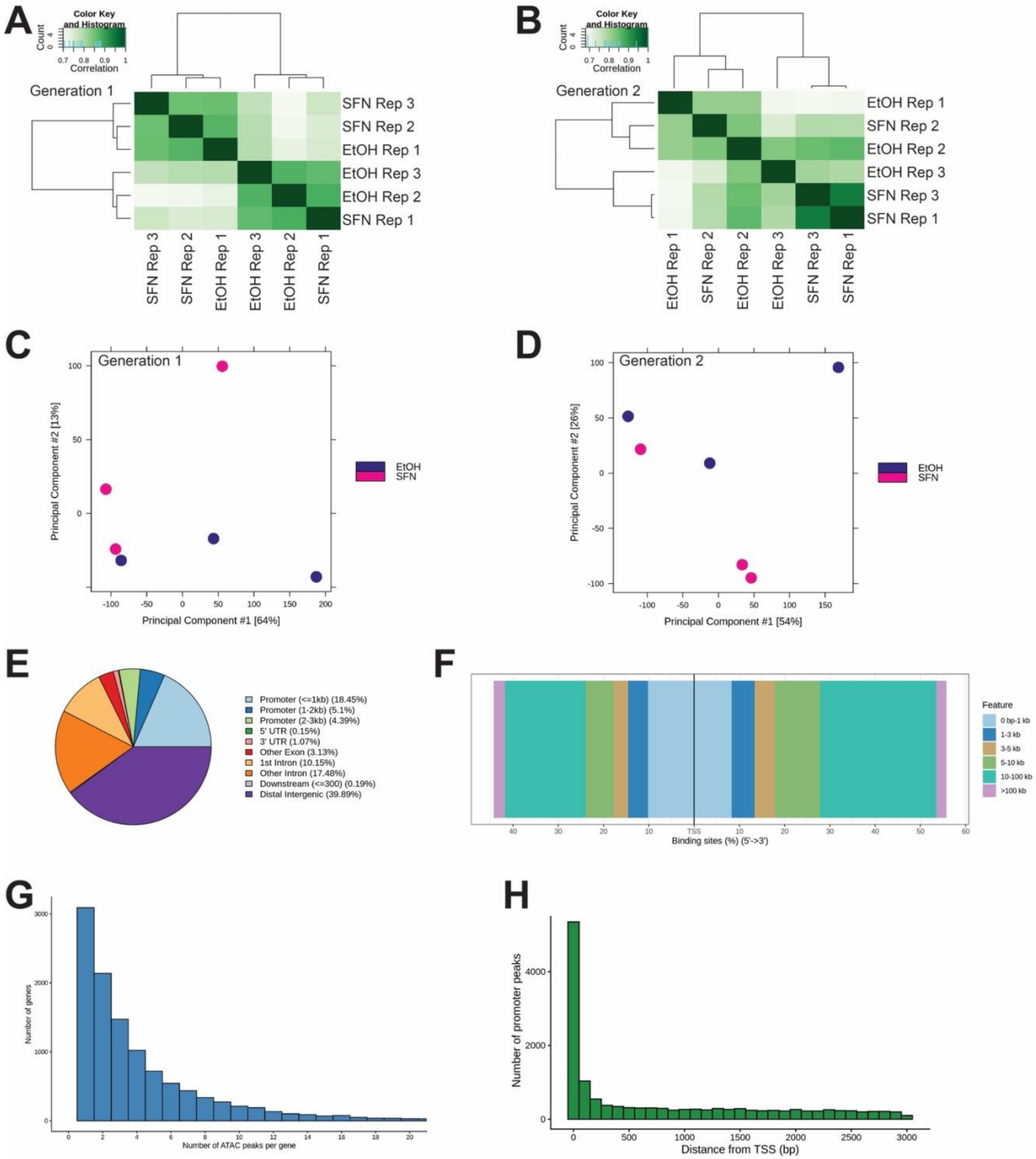

Figure S3, related to Figure 7. Quality assessment and genomic distribution of ATAC-seq peaks from *S. exigua* larvae. **A & B.** Sample-to-sample correlation heatmaps for Generation 1 **(A)** and Generation 2 **(B)**, calculated from normalized read counts across the consensus ATAC-seq peak set. Cell colors represent Pearson correlation coefficients and dendrograms show hierarchical clustering of samples based on correlation distance. **C & D.** Principal component analysis of normalized ATAC-seq read counts for Generation 1 **(C)** and Generation 2 **(D)**. Each point represents one biological replicate and is colored according to treatment. **E.** Genomic annotation of all consensus ATAC-seq peaks using ChIPseeker. **F.** Distribution of consensus peaks relative to annotated transcription start

site (TSS). **G.** Distribution of ATAC-seq peaks per gene. The plot is displayed through 20 peaks per gene for clarity; a small number of genes with more than 20 assigned peaks are not shown. **H.** Distribution of the absolute distance between promoter-associated ATAC-seq peaks and the nearest annotated TSS. Promoter-associated peaks were defined as those located within 3 kb of a TSS.

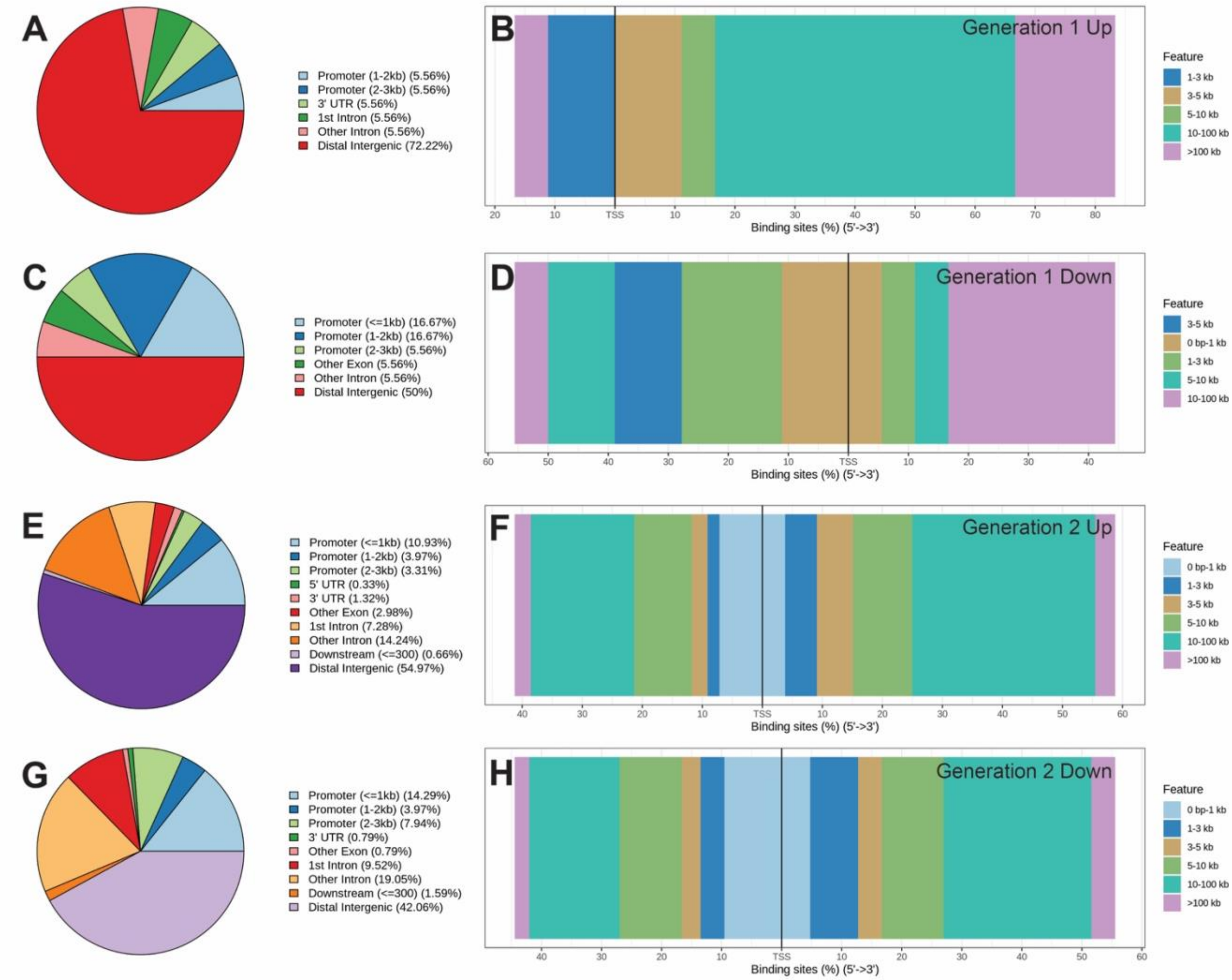

Figure S4, related to Figure 7. Differences in ATAC-seq peak distribution and location between *S. exigua* generations. **A, C, E, G.** Genomic annotation of all consensus ATAC-seq peaks for Generation 1 peaks with increased accessibility **(A)** or decreased accessibility **(C)** and Generation 2 peaks with increased accessibility **(E)** or decreased accessibility **(G)**. **B, D, F, H.** Distribution of consensus peaks relative to annotated transcription start site (TSS) for Generation 1 peaks with increased accessibility **(B)** or decreased accessibility **(D)** and Generation 2 peaks with increased accessibility **(F)** or decreased accessibility **(H)**.

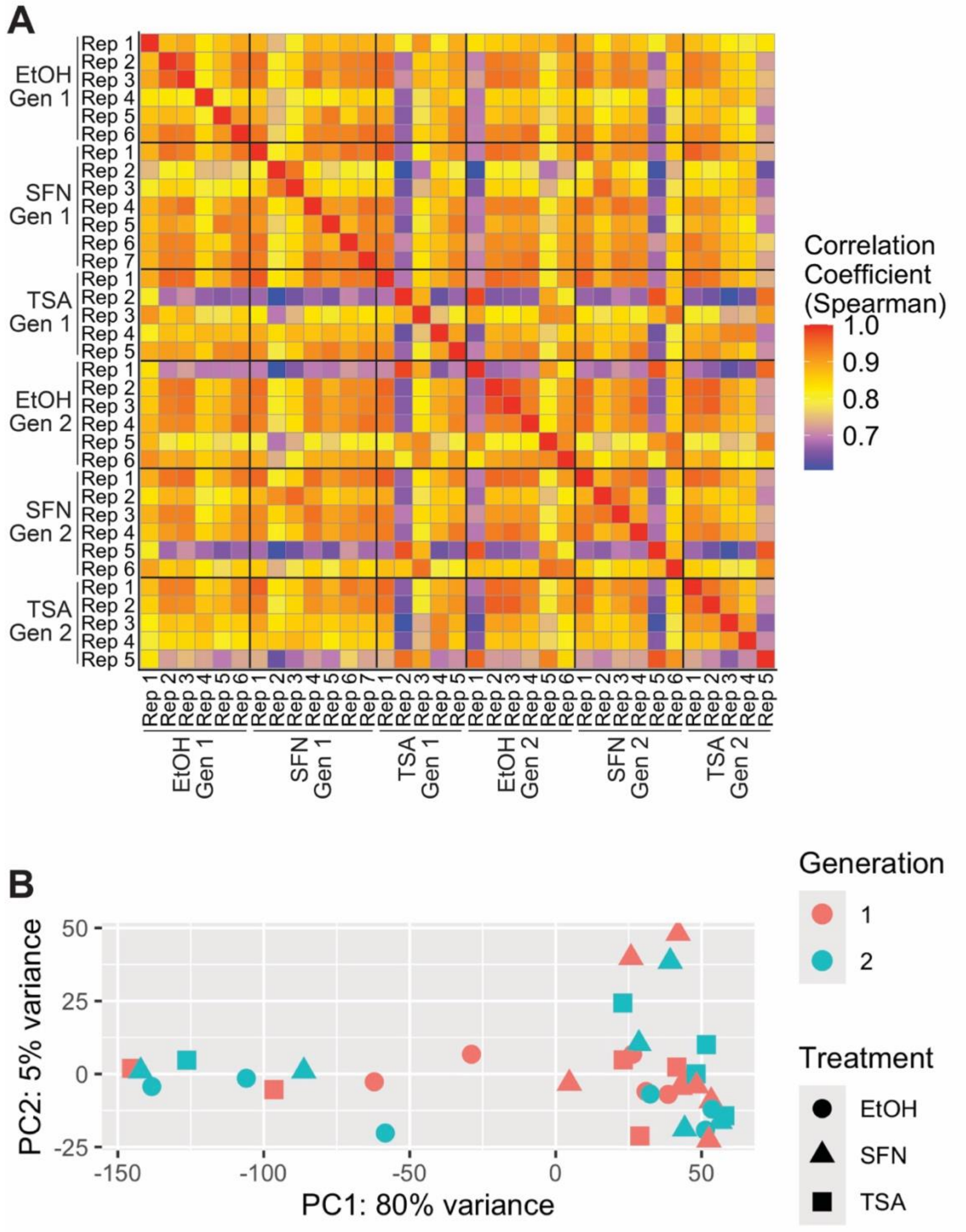

Figure S5, related to Figure 8. Sample-to-sample relationships among RNA-seq libraries for *S. exigua* two-generation experiment. **A.** Heatmap showing the pairwise Spearman correlation coefficients calculated from rlog-transformed gene expression values for all RNA-seq samples in *S. exigua* generation 1 and generation 2 samples. Warmer colors indicate higher correlation between samples. **B.** Principal component analysis (PCA) of rlog-transformed gene expression values shows the proportion of total variance explained by each principal component for *S. exigua* generation 1 and generation 2 samples. Each point represents an individual biological replicate, with colors indicating generation 1 (orange) or generation 2 (turquoise) and shape indicating treatment.

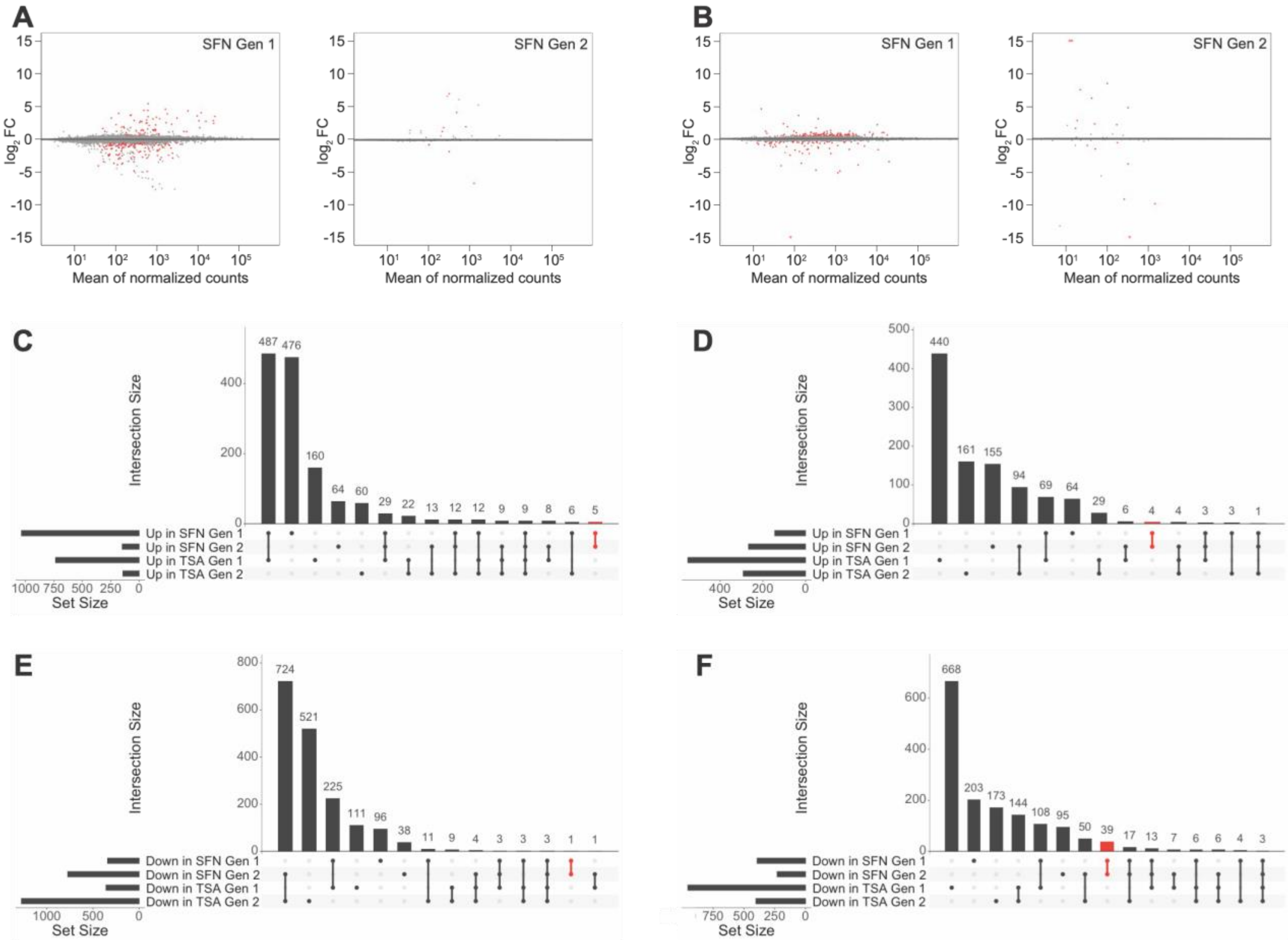

Supplemental Figure S6, related to Figure 8. SFN has no epigenetic effect on *T. ni* over two generations as demonstrated by two independent experiments. **A & B.** MA plots showing differential expression following SFN treatment over two generations in two independent experiments. Significantly differentially expressed genes are indicated as red dots ($p < 0.05$), while non-significant genes are shown in gray. In both experiments, very few genes are significantly differentially expressed in second-generation larvae whose parents were fed a diet of SFN. **C – F.** Intersection plots of the overlap between genes significantly differentially upregulated **(C & D)** or downregulated (**E & F)** in response to SFN or TSA treatment across two generations. Horizontal bars indicate the total number of differentially expressed genes within each set ($p < 0.1$), while vertical bars represent the number of genes shared among each intersection. The sets of genes responding similarly to SFN across generation 1 and generation 2 are highlighted in red. Across both independent experiments, the overlap of genes responding similarly to SFN across generations is minimal.

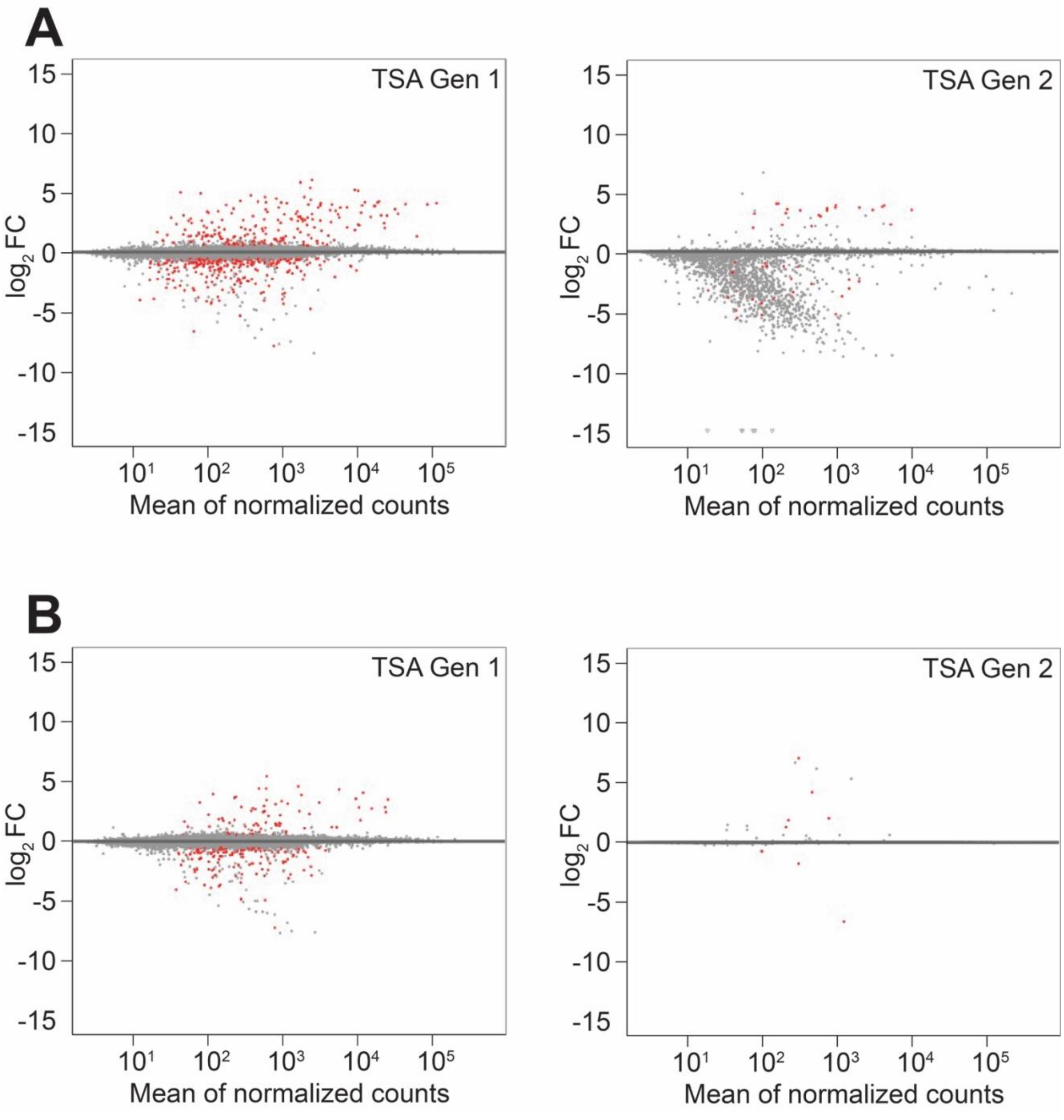

Supplemental Figure S7, related to Figure 8. The strong pharmaceutical HDAC inhibitor TSA has no epigenetic effect on *T. ni* over two generations as demonstrated by two independent experiments. **A & B.** MA plots showing differential expression following TSA treatment over two generations in two independent experiments. Significantly differentially expressed genes are indicated as red dots ($p < 0.05$), while non-significant genes are shown in gray. In both experiments, very few genes are significantly differentially expressed in second-generation larvae whose parents were fed a diet of TSA.

Table S1, related to Figure 2b. Top 50 GO Molecular Function Enrichment for *S. exigua* Cluster SE1

GO.ID	Term	Num in Genome	Num in Cluster	Expected	p-val
GO:0008137	NADH dehydrogenase (ubiquinone) activity	26	21	2.19	8.00E-19
GO:0050136	NADH:ubiquinone reductase (non-electroge...	26	21	2.19	8.00E-19
GO:0046961	proton-transporting ATPase activity, rot...	12	11	1.01	1.50E-11
GO:0004129	cytochrome-c oxidase activity	13	9	1.09	1.00E-07
GO:0003743	translation initiation factor activity	38	14	3.19	1.10E-06
GO:0046933	proton-transporting ATP synthase activit...	7	6	0.59	2.20E-06
GO:0004812	aminoacyl-tRNA ligase activity	35	13	2.94	2.40E-06
GO:0009055	electron transfer activity	81	44	6.81	2.40E-06
GO:0008553	P-type proton-exporting transporter acti...	5	5	0.42	4.10E-06
GO:0016624	oxidoreductase activity, acting on the a...	9	6	0.76	2.30E-05
GO:0022857	transmembrane transporter activity	583	108	49	2.40E-05
GO:0016887	ATP hydrolysis activity	329	48	27.65	9.00E-05
GO:0036185	13-lipoxin reductase activity	9	5	0.76	0.00039
GO:0097257	leukotriene B4 12-hydroxy dehydrogenase ...	9	5	0.76	0.00039
GO:0015453	oxidoreduction-driven active transmembra...	45	34	3.78	0.00053
GO:0044769	ATPase activity, coupled to transmembran...	15	14	1.26	0.00056
GO:0016679	oxidoreductase activity, acting on diphe...	7	4	0.59	0.00141
GO:0051082	unfolded protein binding	77	15	6.47	0.0016
GO:0022853	active monoatomic ion transmembrane tran...	92	53	7.73	0.00178
GO:0015078	proton transmembrane transporter activit...	110	58	9.24	0.00234
GO:0060589	nucleoside-triphosphatase regulator acti...	290	25	24.37	0.00375
GO:0016878	acid-thiol ligase activity	36	8	3.03	0.00513
GO:0016671	oxidoreductase activity, acting on a sul...	5	3	0.42	0.00519
GO:0016635	oxidoreductase activity, acting on the C...	5	3	0.42	0.00519
GO:0015106	bicarbonate transmembrane transporter ac...	15	5	1.26	0.00607
GO:0016491	oxidoreductase activity	447	103	37.57	0.00684
GO:0016289	acyl-CoA hydrolase activity	31	8	2.61	0.00696
GO:0050660	flavin adenine dinucleotide binding	51	11	4.29	0.0085
GO:0005326	neurotransmitter transmembrane transport...	36	8	3.03	0.00864
GO:0008509	monoatomic anion transmembrane transport...	207	29	17.4	0.00932
GO:0005452	solute:inorganic anion antiporter activi...	6	3	0.5	0.00974
GO:0008670	2,4-dienoyl-CoA reductase (NADPH) activi...	6	3	0.5	0.00974
GO:0004448	isocitrate dehydrogenase [NAD(P)+] activ...	6	3	0.5	0.00974
GO:0044183	protein folding chaperone	24	6	2.02	0.01244
GO:0005310	dicarboxylic acid transmembrane transpor...	28	7	2.35	0.014
GO:0071837	HMG box domain binding	12	4	1.01	0.01415
GO:0003954	NADH dehydrogenase activity	33	24	2.77	0.01453
GO:0051087	protein-folding chaperone binding	84	15	7.06	0.0164
GO:0005507	copper ion binding	19	5	1.6	0.01776
GO:0016769	transferase activity, transferring nitro...	21	3	1.76	0.02001
GO:0008135	translation factor activity, RNA binding	66	20	5.55	0.02329
GO:0016209	antioxidant activity	44	7	3.7	0.02388
GO:0015295	solute:proton symporter activity	33	6	2.77	0.02389
GO:0031957	very long-chain fatty acid-CoA ligase ac...	8	3	0.67	0.024
GO:0019166	trans-2-enoyl-CoA reductase (NADPH) acti...	8	3	0.67	0.024
GO:0043295	glutathione binding	8	3	0.67	0.024
GO:0047617	fatty acyl-CoA hydrolase activity	29	6	2.44	0.03074
GO:0000062	fatty-acyl-CoA binding	15	4	1.26	0.03189
GO:0001671	ATPase activator activity	22	5	1.85	0.03265
GO:0009881	photoreceptor activity	9	3	0.76	0.03379

Table S2, related to Figure 2b. Top 50 GO Biological Process Enrichment for *S. exigua* Cluster SE1

GO.ID	Term	Num in Genome	Num in Cluster	Expected	p-val
GO:0046034	ATP metabolic process	187	77	15.05	1.80E-27
GO:0016310	phosphorylation	1094	127	88.02	1.50E-26
GO:0009167	purine ribonucleoside monophosphate meta...	177	74	14.24	7.00E-25
GO:0006120	mitochondrial electron transport, NADH t...	36	26	2.9	2.50E-21
GO:0032981	mitochondrial respiratory chain complex ...	41	25	3.3	8.10E-18
GO:0015988	energy coupled proton transmembrane tran...	16	15	1.29	4.80E-16
GO:0015985	energy coupled proton transport, down el...	21	17	1.69	8.80E-16
GO:0016999	antibiotic metabolic process	83	28	6.68	8.00E-14
GO:0009168	purine ribonucleoside monophosphate bios...	66	27	5.31	2.00E-13
GO:0006099	tricarboxylic acid cycle	40	21	3.22	2.30E-13
GO:0006101	citrate metabolic process	43	21	3.46	1.40E-12
GO:0042407	cristae formation	21	14	1.69	2.80E-11
GO:0042776	proton motive force-driven mitochondrial...	14	11	1.13	2.40E-10
GO:0007035	vacuolar acidification	14	10	1.13	7.90E-09
GO:1990542	mitochondrial transmembrane transport	66	20	5.31	1.70E-08
GO:0006104	succinyl-CoA metabolic process	7	7	0.56	2.10E-08
GO:0006122	mitochondrial electron transport, ubiqui...	8	7	0.64	1.60E-07
GO:0033572	transferrin transport	22	11	1.77	2.60E-07
GO:0006103	2-oxoglutarate metabolic process	23	11	1.85	4.60E-07
GO:0045851	pH reduction	23	11	1.85	4.60E-07
GO:0015986	proton motive force-driven ATP synthesis	21	17	1.69	1.60E-06
GO:0006123	mitochondrial electron transport, cytoch...	10	7	0.8	2.00E-06
GO:0046496	nicotinamide nucleotide metabolic proces...	99	30	7.97	5.40E-06
GO:0019674	NAD metabolic process	45	16	3.62	6.00E-06
GO:0006086	pyruvate decarboxylation to acetyl-CoA	13	7	1.05	6.40E-06
GO:0006418	tRNA aminoacylation for protein translat...	39	13	3.14	6.90E-06
GO:0019752	carboxylic acid metabolic process	644	108	51.81	1.00E-05
GO:0006734	NADH metabolic process	34	14	2.74	1.10E-05
GO:0043043	peptide biosynthetic process	343	49	27.6	1.60E-05
GO:0018335	protein succinylation	6	5	0.48	1.90E-05
GO:0043068	positive regulation of programmed cell d...	375	39	30.17	2.10E-05
GO:1904950	negative regulation of establishment of ...	107	8	8.61	4.30E-05
GO:0098771	inorganic ion homeostasis	328	46	26.39	7.80E-05
GO:0001731	formation of translation preinitiation c...	11	6	0.89	8.60E-05
GO:0043604	amide biosynthetic process	413	62	33.23	0.00011
GO:1902600	proton transmembrane transport	104	44	8.37	0.00013
GO:0006749	glutathione metabolic process	49	13	3.94	0.00023
GO:0034032	purine nucleoside bisphosphate metabolic...	120	26	9.65	0.00023
GO:0033875	ribonucleoside bisphosphate metabolic pr...	120	26	9.65	0.00023
GO:0046907	intracellular transport	1269	114	102.1	0.00027
GO:0035149	lumen formation, open tracheal system	23	8	1.85	0.00028
GO:1903427	negative regulation of reactive oxygen s...	13	6	1.05	0.00028
GO:0022900	electron transport chain	115	55	9.25	0.00029
GO:0036295	cellular response to increased oxygen le...	9	5	0.72	0.00032
GO:0046689	response to mercury ion	14	6	1.13	0.00045
GO:0009914	hormone transport	193	13	15.53	0.00049
GO:0000041	transition metal ion transport	84	19	6.76	0.00049
GO:0015732	prostaglandin transport	21	6	1.69	0.00051
GO:0019748	secondary metabolic process	134	15	10.78	0.00051
GO:0006458	'de novo' protein folding	26	11	2.09	0.00057

Table S3, related to Figure 2c. Top 50 GO Molecular Function Enrichment for *S. exigua* Cluster SE2

GO.ID	Term	Num in Genome	Num in Cluster	Expected	p-val
GO:0008061	chitin binding	26	15	1.31	1.10E-13
GO:0004568	chitinase activity	19	11	0.95	2.30E-10
GO:0030165	PDZ domain binding	65	13	3.26	1.60E-05
GO:0004601	peroxidase activity	31	8	1.56	0.00011
GO:0034041	ABC-type sterol transporter activity	12	5	0.6	0.00018
GO:0008514	organic anion transmembrane transporter ...	174	16	8.74	0.00166
GO:0005121	Toll binding	6	3	0.3	0.00224
GO:0008236	serine-type peptidase activity	148	18	7.43	0.00238
GO:0008083	growth factor activity	14	4	0.7	0.00418
GO:0004222	metalloendopeptidase activity	43	7	2.16	0.0051
GO:0019213	deacetylase activity	38	4	1.91	0.00584
GO:0004252	serine-type endopeptidase activity	132	14	6.63	0.00621
GO:0080019	alcohol-forming very long-chain fatty ac...	9	3	0.45	0.0084
GO:0004312	fatty acid synthase activity	19	4	0.95	0.01328
GO:0005214	structural constituent of chitin-based c...	23	3	1.15	0.0141
GO:0015318	inorganic molecular entity transmembrane...	451	23	22.65	0.01696
GO:0004181	metallocarboxypeptidase activity	31	5	1.56	0.01792
GO:0010181	FMN binding	12	3	0.6	0.01967
GO:0004715	non-membrane spanning protein tyrosine k...	22	4	1.1	0.02228
GO:0035175	histone H3S10 kinase activity	5	2	0.25	0.02272
GO:0004169	dolichyl-phosphate-mannose-protein manno...	5	2	0.25	0.02272
GO:0005119	smoothened binding	5	2	0.25	0.02272
GO:0030297	transmembrane receptor protein tyrosine ...	5	2	0.25	0.02272
GO:0098599	palmitoyl hydrolase activity	5	2	0.25	0.02272
GO:0009922	fatty acid elongase activity	13	3	0.65	0.02464
GO:0015020	glucuronosyltransferase activity	36	5	1.81	0.03246
GO:0015291	secondary active transmembrane transport...	101	9	5.07	0.03254
GO:0047144	2-acylglycerol-3-phosphate O-acyltransfe...	6	2	0.3	0.03296
GO:0008509	monoatomic anion transmembrane transport...	207	17	10.39	0.03496
GO:0016174	NAD(P)H oxidase H2O2-forming activity	7	2	0.35	0.04464
GO:0008046	axon guidance receptor activity	7	2	0.35	0.04464
GO:0001161	intronic transcription regulatory region...	7	2	0.35	0.04464
GO:0004930	G protein-coupled receptor activity	92	8	4.62	0.05723
GO:0000987	cis-regulatory region sequence-specific ...	214	13	10.75	0.05737
GO:0033293	monocarboxylic acid binding	38	4	1.91	0.05743
GO:0050431	transforming growth factor beta binding	8	2	0.4	0.05758
GO:0043035	chromatin insulator sequence binding	8	2	0.4	0.05758
GO:0004190	aspartic-type endopeptidase activity	8	2	0.4	0.05758
GO:0047372	monoacylglycerol lipase activity	8	2	0.4	0.05758
GO:0031957	very long-chain fatty acid-CoA ligase ac...	8	2	0.4	0.05758
GO:0015245	fatty acid transmembrane transporter act...	8	2	0.4	0.05758
GO:0016705	oxidoreductase activity, acting on paire...	63	7	3.16	0.05992
GO:0004565	beta-galactosidase activity	19	3	0.95	0.06696
GO:0004467	long-chain fatty acid-CoA ligase activit...	19	3	0.95	0.06696
GO:0038187	pattern recognition receptor activity	9	2	0.45	0.07163
GO:0008201	heparin binding	32	4	1.61	0.07424
GO:0005201	extracellular matrix structural constitu...	20	3	1	0.07596
GO:0030246	carbohydrate binding	67	7	3.36	0.07847
GO:0008017	microtubule binding	137	11	6.88	0.08167
GO:0070402	NADPH binding	10	2	0.5	0.08665

Table S4, related to Figure 2c. Top 50 GO Biological Process Enrichment for *S. exigua* Cluster SE2

GO.ID	Term	Num in Genome	Num in Cluster	Expected	p-val
GO:0006032	chitin catabolic process	20	11	1.02	6.10E-10
GO:0018990	ecdysis, chitin-based cuticle	14	7	0.72	2.20E-06
GO:0042730	fibrinolysis	5	4	0.26	3.20E-05
GO:0030574	collagen catabolic process	16	6	0.82	8.90E-05
GO:0035159	regulation of tube length, open tracheal...	38	9	1.95	9.50E-05
GO:0006584	catecholamine metabolic process	31	5	1.59	0.00013
GO:0040003	chitin-based cuticle development	89	16	4.56	0.00016
GO:0030299	intestinal cholesterol absorption	7	4	0.36	0.00021
GO:0018958	phenol-containing compound metabolic pro...	98	14	5.02	0.00049
GO:0048085	adult chitin-containing cuticle pigmenta...	32	9	1.64	0.00062
GO:0006570	tyrosine metabolic process	21	7	1.08	0.00068
GO:0006968	cellular defense response	9	4	0.46	0.00069
GO:0008362	chitin-based embryonic cuticle biosynthe...	23	6	1.18	0.00083
GO:0098869	cellular oxidant detoxification	59	9	3.02	0.00094
GO:0035025	positive regulation of Rho protein signa...	24	6	1.23	0.00106
GO:0071679	commissural neuron axon guidance	17	5	0.87	0.00127
GO:0035269	protein O-linked mannosylation	11	4	0.56	0.00167
GO:0061365	positive regulation of triglyceride lipa...	6	3	0.31	0.00237
GO:0030514	negative regulation of BMP signaling pat...	40	7	2.05	0.00375
GO:0097264	self proteolysis	7	3	0.36	0.00399
GO:2000096	positive regulation of Wnt signaling pat...	7	3	0.36	0.00399
GO:0098773	skin epidermis development	51	4	2.61	0.00403
GO:0001656	metanephros development	42	8	2.15	0.00524
GO:0060026	convergent extension	23	4	1.18	0.00614
GO:0048665	neuron fate specification	24	5	1.23	0.00649
GO:0048731	system development	2922	172	149.6	0.00674
GO:0019377	glycolipid catabolic process	28	5	1.43	0.00751
GO:0090132	epithelium migration	301	15	15.41	0.00767
GO:0008049	male courtship behavior	43	6	2.2	0.00778
GO:0050830	defense response to Gram-positive bacter...	69	9	3.53	0.00817
GO:0018149	peptide cross-linking	9	3	0.46	0.00888
GO:1904778	positive regulation of protein localizat...	9	3	0.46	0.00888
GO:0010025	wax biosynthetic process	9	3	0.46	0.00888
GO:0045752	positive regulation of Toll signaling pa...	9	3	0.46	0.00888
GO:0070098	chemokine-mediated signaling pathway	9	3	0.46	0.00888
GO:0007402	ganglion mother cell fate determination	9	3	0.46	0.00888
GO:0046579	positive regulation of Ras protein signa...	47	7	2.41	0.00932
GO:0007156	homophilic cell adhesion via plasma memb...	47	7	2.41	0.00932
GO:0048800	antennal morphogenesis	17	4	0.87	0.00946
GO:0042335	cuticle development	174	33	8.91	0.01034
GO:0032094	response to food	28	6	1.43	0.01152
GO:0021522	spinal cord motor neuron differentiation	18	4	0.92	0.01168
GO:0048082	regulation of adult chitin-containing cu...	10	3	0.51	0.0122
GO:0044539	long-chain fatty acid import into cell	10	3	0.51	0.0122
GO:0045669	positive regulation of osteoblast differ...	28	5	1.43	0.0127
GO:0048867	stem cell fate determination	28	5	1.43	0.0127
GO:0014019	neuroblast development	19	4	0.97	0.01421
GO:0008654	phospholipid biosynthetic process	167	7	8.55	0.01481
GO:0051603	proteolysis involved in protein cataboli...	445	19	22.78	0.01514
GO:0036011	imaginal disc-derived leg segmentation	26	5	1.33	0.01601

Table S5, related to Figure 2d. Top 50 GO Molecular Function Enrichment for *S. exigua* Cluster SE3

GO.ID	Term	Num in Genome	Num in Cluster	Expected	p-val
GO:0003796	lysozyme activity	5	4	0.25	3.10E-05
GO:0080025	phosphatidylinositol-3,5-bisphosphate bi...	18	5	0.91	0.0016
GO:0047499	calcium-independent phospholipase A2 act...	6	3	0.3	0.0023
GO:0017171	serine hydrolase activity	154	7	7.8	0.0024
GO:0000978	RNA polymerase II cis-regulatory region ...	206	21	10.44	0.0024
GO:0015925	galactosidase activity	21	3	1.06	0.0026
GO:0016597	amino acid binding	39	7	1.98	0.003
GO:0016884	carbon-nitrogen ligase activity, with gl...	12	4	0.61	0.0038
GO:0016679	oxidoreductase activity, acting on diphe...	7	3	0.35	0.0039
GO:0016812	hydrolase activity, acting on carbon-nit...	7	3	0.35	0.0039
GO:0050699	WW domain binding	14	4	0.71	0.0043
GO:0032549	ribonucleoside binding	109	6	5.52	0.0075
GO:0008010	structural constituent of chitin-based l...	19	4	0.96	0.0137
GO:0005214	structural constituent of chitin-based c...	23	6	1.17	0.0141
GO:0004385	guanylate kinase activity	12	3	0.61	0.0202
GO:0060089	molecular transducer activity	252	19	12.77	0.0225
GO:0004096	catalase activity	5	2	0.25	0.0231
GO:0008526	phosphatidylinositol transfer activity	5	2	0.25	0.0231
GO:0052659	inositol-1,3,4,5-tetrakisphosphate 5-pho...	5	2	0.25	0.0231
GO:1904288	BAT3 complex binding	5	2	0.25	0.0231
GO:0051864	histone H3K36 demethylase activity	5	2	0.25	0.0231
GO:0032557	pyrimidine ribonucleotide binding	5	2	0.25	0.0231
GO:0016303	1-phosphatidylinositol-3-kinase activity	13	3	0.66	0.0252
GO:0034450	ubiquitin-ubiquitin ligase activity	13	3	0.66	0.0252
GO:0031406	carboxylic acid binding	97	8	4.91	0.0289
GO:0004879	nuclear receptor activity	25	5	1.27	0.0306
GO:0016705	oxidoreductase activity, acting on paire...	63	12	3.19	0.0332
GO:0004435	phosphatidylinositol phospholipase C act...	6	2	0.3	0.0335
GO:0016500	protein-hormone receptor activity	6	2	0.3	0.0335
GO:0004040	amidase activity	6	2	0.3	0.0335
GO:0035650	AP-1 adaptor complex binding	6	2	0.3	0.0335
GO:0016741	transferase activity, transferring one-c...	121	6	6.13	0.0339
GO:0042803	protein homodimerization activity	557	38	28.22	0.0344
GO:0000149	SNARE binding	81	8	4.1	0.0349
GO:0051427	hormone receptor binding	121	11	6.13	0.0423
GO:0005096	GTPase activator activity	136	12	6.89	0.0424
GO:0051020	GTPase binding	374	25	18.95	0.0447
GO:0005164	tumor necrosis factor receptor binding	7	2	0.35	0.0454
GO:0005159	insulin-like growth factor receptor bind...	7	2	0.35	0.0454
GO:0016231	beta-N-acetylglucosaminidase activity	7	2	0.35	0.0454
GO:0043560	insulin receptor substrate binding	7	2	0.35	0.0454
GO:0019207	kinase regulator activity	125	6	6.33	0.0459
GO:0052827	inositol pentakisphosphate phosphatase a...	6	2	0.3	0.0506
GO:0106019	phosphatidylinositol-4,5-bisphosphate ph...	7	2	0.35	0.0506
GO:0004497	monooxygenase activity	36	5	1.82	0.0513
GO:0005109	frizzled binding	17	3	0.86	0.0517
GO:0046982	protein heterodimerization activity	269	20	13.63	0.0536
GO:0045545	syndecan binding	8	2	0.41	0.0585
GO:0016709	oxidoreductase activity, acting on paire...	8	2	0.41	0.0585
GO:0032453	histone H3K4 demethylase activity	8	2	0.41	0.0585

Table S6, related to Figure 2d. Top 50 GO Biological Process Enrichment for *S. exigua* Cluster SE3

GO.ID	Term	Num in Genome	Num in Cluster	Expected	p-val
GO:0007480	imaginal disc-derived leg morphogenesis	77	13	4	4.70E-05
GO:0097164	ammonium ion metabolic process	90	16	4.68	0.00036
GO:0036151	phosphatidylcholine acyl-chain remodelin...	9	4	0.47	0.00074
GO:0009208	pyrimidine ribonucleoside triphosphate m...	5	3	0.26	0.00129
GO:1904637	cellular response to ionomycin	5	3	0.26	0.00129
GO:0006583	melanin biosynthetic process from tyrosi...	5	3	0.26	0.00129
GO:0042417	dopamine metabolic process	24	6	1.25	0.00175
GO:0042381	hemolymph coagulation	11	4	0.57	0.00178
GO:0006541	glutamine metabolic process	19	5	0.99	0.00235
GO:0035493	SNARE complex assembly	19	5	0.99	0.00246
GO:0071985	multivesicular body sorting pathway	38	5	1.98	0.00248
GO:0009148	pyrimidine nucleoside triphosphate biosy...	6	3	0.31	0.00248
GO:0006041	glucosamine metabolic process	6	3	0.31	0.00248
GO:0097210	response to gonadotropin-releasing hormo...	6	3	0.31	0.00248
GO:0072567	chemokine (C-X-C motif) ligand 2 product...	6	3	0.31	0.00248
GO:0031638	zymogen activation	17	5	0.88	0.00254
GO:0170043	non-proteinogenic amino acid biosyntheti...	12	4	0.62	0.00255
GO:0035721	intraciliary retrograde transport	12	4	0.62	0.00255
GO:0008219	cell death	1094	51	56.9	0.00267
GO:0035210	prepupal development	13	4	0.68	0.00268
GO:1901072	glucosamine-containing compound cataboli...	22	4	1.14	0.00268
GO:0016447	somatic recombination of immunoglobulin ...	25	4	1.3	0.00269
GO:0002495	antigen processing and presentation of p...	56	5	2.91	0.0027
GO:0010595	positive regulation of endothelial cell ...	68	8	3.54	0.00326
GO:0032655	regulation of interleukin-12 production	15	4	0.78	0.00416
GO:2000353	positive regulation of endothelial cell ...	7	3	0.36	0.00418
GO:0061113	pancreas morphogenesis	7	3	0.36	0.00418
GO:0061635	regulation of protein complex stability	7	3	0.36	0.00418
GO:0007365	periodic partitioning	42	4	2.18	0.0042
GO:0000045	autophagosome assembly	75	10	3.9	0.00457
GO:0048745	smooth muscle tissue development	14	4	0.73	0.00475
GO:0016322	neuron remodeling	70	9	3.64	0.005
GO:0007165	signal transduction	2464	134	128.16	0.00593
GO:0007436	larval salivary gland morphogenesis	15	4	0.78	0.00622
GO:0030213	hyaluronan biosynthetic process	8	3	0.42	0.00643
GO:0006048	UDP-N-acetylglucosamine biosynthetic pro...	8	3	0.42	0.00643
GO:0036152	phosphatidylethanolamine acyl-chain remo...	8	3	0.42	0.00643
GO:0032885	regulation of polysaccharide biosyntheti...	17	4	0.88	0.00777
GO:0097720	calcineurin-mediated signaling	29	4	1.51	0.00779
GO:0009057	macromolecule catabolic process	929	49	48.32	0.00787
GO:0071900	regulation of protein serine/threonine k...	257	13	13.37	0.00791
GO:0010614	negative regulation of cardiac muscle hy...	16	5	0.83	0.00916
GO:0042093	T-helper cell differentiation	15	4	0.78	0.00923
GO:2001135	regulation of endocytic recycling	20	4	1.04	0.00924
GO:0050482	arachidonate secretion	9	3	0.47	0.00927
GO:0042428	serotonin metabolic process	9	3	0.47	0.00927
GO:0016114	terpenoid biosynthetic process	9	3	0.47	0.00927
GO:0001553	luteinization	9	3	0.47	0.00927
GO:0006716	juvenile hormone metabolic process	9	3	0.47	0.00927
GO:0002218	activation of innate immune response	126	8	6.55	0.00934

Table S7, related to Figure 3b. Top 50 GO Molecular Function Enrichment for *T. ni* Cluster TN1

GO.ID	Term	Num in Genome	Num in Cluster	Expected	p-val
GO:0005214	structural constituent of chitin-based c...	37	13	1.85	1.10E-08
GO:0042302	structural constituent of cuticle	37	13	1.85	1.10E-08
GO:0008010	structural constituent of chitin-based l...	34	12	1.7	3.90E-08
GO:0030246	carbohydrate binding	72	12	3.59	0.0002
GO:0015271	outward rectifier potassium channel acti...	4	3	0.2	0.00047
GO:0099604	ligand-gated calcium channel activity	15	5	0.75	0.00059
GO:0099094	ligand-gated monoatomic cation channel a...	42	8	2.1	0.00093
GO:0009922	fatty acid elongase activity	10	4	0.5	0.001
GO:0004553	hydrolase activity, hydrolyzing O-glycos...	79	11	3.94	0.00171
GO:0004308	exo-alpha-sialidase activity	2	2	0.1	0.00248
GO:0016997	alpha-sialidase activity	2	2	0.1	0.00248
GO:0030882	lipid antigen binding	2	2	0.1	0.00248
GO:0070891	lipoteichoic acid binding	2	2	0.1	0.00248
GO:0080019	alcohol-forming very long-chain fatty ac...	7	3	0.35	0.0037
GO:0016798	hydrolase activity, acting on glycosyl b...	87	11	4.34	0.00373
GO:0004312	fatty acid synthase activity	14	4	0.7	0.00408
GO:0043177	organic acid binding	102	12	5.09	0.00455
GO:0015276	ligand-gated monoatomic ion channel acti...	54	8	2.69	0.00489
GO:0008194	UDP-glycosyltransferase activity	78	10	3.89	0.00506
GO:0005518	collagen binding	24	5	1.2	0.0058
GO:0008061	chitin binding	24	5	1.2	0.0058
GO:0005267	potassium channel activity	34	6	1.7	0.00603
GO:0015020	glucuronosyltransferase activity	35	6	1.75	0.00699
GO:0005262	calcium channel activity	46	7	2.29	0.00717
GO:0070569	uridylyltransferase activity	3	2	0.15	0.0072
GO:0015278	intracellularly gated calcium channel ac...	9	3	0.45	0.00825
GO:0016702	oxidoreductase activity, acting on singl...	9	3	0.45	0.00825
GO:0052743	inositol tetrakisphosphate phosphatase a...	9	3	0.45	0.00825
GO:0031406	carboxylic acid binding	99	11	4.94	0.00989
GO:0005201	extracellular matrix structural constitu...	18	4	0.9	0.01066
GO:0005217	intracellularly ligand-gated monoatomic ...	19	4	0.95	0.01298
GO:0005261	monoatomic cation channel activity	103	11	5.14	0.01313
GO:0004446	inositol-hexakisphosphate phosphatase ac...	4	2	0.2	0.01392
GO:0005251	delayed rectifier potassium channel acti...	4	2	0.2	0.01392
GO:0015277	kainate selective glutamate receptor act...	4	2	0.2	0.01392
GO:0015280	ligand-gated sodium channel activity	4	2	0.2	0.01392
GO:0030351	inositol-1,3,4,5,6-pentakisphosphate 3-p...	4	2	0.2	0.01392
GO:0030352	inositol-1,4,5,6-tetrakisphosphate 6-pho...	4	2	0.2	0.01392
GO:0034417	bisphosphoglycerate 3-phosphatase activi...	4	2	0.2	0.01392
GO:0043522	leucine zipper domain binding	4	2	0.2	0.01392
GO:0052826	inositol hexakisphosphate 2-phosphatase ...	4	2	0.2	0.01392
GO:0016701	oxidoreductase activity, acting on singl...	11	3	0.55	0.01504
GO:0051861	glycolipid binding	11	3	0.55	0.01504
GO:0015085	calcium ion transmembrane transporter ac...	54	7	2.69	0.01692
GO:0015171	amino acid transmembrane transporter act...	54	7	2.69	0.01692
GO:0022834	ligand-gated channel activity	80	9	3.99	0.01744
GO:0022839	monoatomic ion-gated channel activity	80	9	3.99	0.01744
GO:0008238	exopeptidase activity	67	8	3.34	0.01755
GO:0046873	metal ion transmembrane transporter acti...	151	14	7.53	0.01798
GO:0035035	histone acetyltransferase binding	21	4	1.05	0.01853

Table S8, related to Figure 3b. Top 50 GO Biological Process Enrichment for *T. ni* Cluster TN1

GO.ID	Term	Num in Genome	Num in Cluster	Expected	p-val
GO:0040003	chitin-based cuticle development	93	17	4.48	6.60E-08
GO:0014019	neuroblast development	15	5	0.72	0.0005
GO:0001655	urogenital system development	211	21	10.15	0.00081
GO:0034625	fatty acid elongation, monounsaturated f...	10	4	0.48	0.00088
GO:0034626	fatty acid elongation, polyunsaturated f...	10	4	0.48	0.00088
GO:0019367	fatty acid elongation, saturated fatty a...	10	4	0.48	0.00088
GO:0003384	apical constriction involved in gastrula...	17	5	0.82	0.00096
GO:1904714	regulation of chaperone-mediated autopha...	5	3	0.24	0.00103
GO:0001822	kidney development	154	17	7.41	0.00103
GO:0006953	acute-phase response	27	6	1.3	0.00149
GO:0048864	stem cell development	47	7	2.26	0.00165
GO:0060025	regulation of synaptic activity	12	4	0.58	0.00192
GO:0021554	optic nerve development	6	3	0.29	0.00198
GO:1903051	negative regulation of proteolysis invol...	49	3	2.36	0.00233
GO:0042761	very long-chain fatty acid biosynthetic ...	13	4	0.63	0.00266
GO:0010025	wax biosynthetic process	7	3	0.34	0.00334
GO:0048570	notochord morphogenesis	7	3	0.34	0.00334
GO:0007492	endoderm development	56	8	2.7	0.00403
GO:0030324	lung development	94	10	4.52	0.00456
GO:0046513	ceramide biosynthetic process	20	5	0.96	0.00467
GO:0070831	basement membrane assembly	8	3	0.39	0.00516
GO:0006212	uracil catabolic process	8	3	0.39	0.00516
GO:0031100	animal organ regeneration	78	10	3.75	0.00579
GO:0006534	cysteine metabolic process	14	4	0.67	0.00665
GO:0045912	negative regulation of carbohydrate meta...	41	7	1.97	0.00672
GO:0008358	maternal determination of anterior/poste...	83	3	3.99	0.00683
GO:0008293	torso signaling pathway	26	5	1.25	0.00714
GO:0014009	glial cell proliferation	42	7	2.02	0.00731
GO:2000027	regulation of animal organ morphogenesis	165	13	7.94	0.00732
GO:0035336	long-chain fatty-acyl-CoA metabolic proc...	17	4	0.82	0.00743
GO:0002823	negative regulation of adaptive immune r...	9	3	0.43	0.00747
GO:0044344	cellular response to fibroblast growth f...	75	6	3.61	0.00748
GO:0035214	eye-antennal disc development	43	4	2.07	0.00751
GO:0032501	multicellular organismal process	3983	227	191.69	0.00761
GO:0034032	purine nucleoside bisphosphate metabolic...	107	10	5.15	0.00761
GO:0033875	ribonucleoside bisphosphate metabolic pr...	107	10	5.15	0.00761
GO:0071385	cellular response to glucocorticoid stim...	59	8	2.84	0.00875
GO:0051289	protein homotetramerization	50	7	2.41	0.0094
GO:0006687	glycosphingolipid metabolic process	44	8	2.12	0.01
GO:0008299	isoprenoid biosynthetic process	18	4	0.87	0.01024
GO:0032331	negative regulation of chondrocyte diffe...	10	3	0.48	0.01029
GO:0007488	histoblast morphogenesis	10	3	0.48	0.01029
GO:0001889	liver development	148	15	7.12	0.01056
GO:0050878	regulation of body fluid levels	159	13	7.65	0.0127
GO:0042364	water-soluble vitamin biosynthetic proce...	22	4	1.06	0.0129
GO:0048704	embryonic skeletal system morphogenesis	54	7	2.6	0.01343
GO:0021846	cell proliferation in forebrain	11	3	0.53	0.01366
GO:0021602	cranial nerve morphogenesis	11	3	0.53	0.01366
GO:0007613	memory	166	14	7.99	0.01379
GO:0007413	axonal fasciculation	42	6	2.02	0.01438

Table S9, related to Figure 3c. Top 50 GO Molecular Function Enrichment for *T. ni* Cluster TN2

GO.ID	Term	Num in Genome	Num in Cluster	Expected	p-val
GO:0004252	serine-type endopeptidase activity	106	19	2.84	2.80E-11
GO:0015036	disulfide oxidoreductase activity	29	6	0.78	5.40E-05
GO:0003756	protein disulfide isomerase activity	14	4	0.38	0.0004
GO:0016209	antioxidant activity	47	5	1.26	0.0014
GO:0090599	alpha-glucosidase activity	9	3	0.24	0.0014
GO:0004957	prostaglandin E receptor activity	10	3	0.27	0.002
GO:0052689	carboxylic ester hydrolase activity	108	11	2.9	0.0023
GO:0008146	sulfotransferase activity	14	3	0.38	0.0055
GO:0004467	long-chain fatty acid-CoA ligase activit...	16	3	0.43	0.0082
GO:0070403	NAD+ binding	16	3	0.43	0.0082
GO:0016616	oxidoreductase activity, acting on the C...	83	10	2.23	0.01
GO:0016404	15-hydroxyprostaglandin dehydrogenase (N...	18	3	0.48	0.0115
GO:0016857	racemase and epimerase activity, acting ...	8	2	0.21	0.018
GO:0016298	lipase activity	72	6	1.93	0.021
GO:0019825	oxygen binding	9	2	0.24	0.0227
GO:0008106	alcohol dehydrogenase (NADP+) activity	10	2	0.27	0.0279
GO:0030145	manganese ion binding	26	3	0.7	0.0313
GO:0004341	gluconolactonase activity	11	2	0.29	0.0335
GO:0019239	deaminase activity	11	2	0.29	0.0335
GO:0008096	juvenile hormone epoxide hydrolase activ...	11	2	0.29	0.0335
GO:0033961	cis-stilbene-oxide hydrolase activity	11	2	0.29	0.0335
GO:0016814	hydrolase activity, acting on carbon-nit...	14	2	0.38	0.0526
GO:0001882	nucleoside binding	106	3	2.84	0.0532
GO:0008670	2,4-dienoyl-CoA reductase (NADPH) activi...	15	2	0.4	0.0597
GO:0019166	trans-2-enoyl-CoA reductase (NADPH) acti...	16	2	0.43	0.067
GO:0030170	pyridoxal phosphate binding	18	2	0.48	0.0826
GO:0016651	oxidoreductase activity, acting on NAD(P...	67	4	1.8	0.0906
GO:0017171	serine hydrolase activity	127	20	3.41	0.093
GO:0016620	oxidoreductase activity, acting on the a...	31	3	0.83	0.0983
GO:0016878	acid-thiol ligase activity	34	5	0.91	0.101
GO:0030246	carbohydrate binding	72	4	1.93	0.1015
GO:0016854	racemase and epimerase activity	12	3	0.32	0.1019
GO:0061659	ubiquitin-like protein ligase activity	129	2	3.46	0.1045
GO:0005507	copper ion binding	21	2	0.56	0.1077
GO:0002162	dystroglycan binding	5	1	0.13	0.1271
GO:0016672	oxidoreductase activity, acting on a sul...	5	1	0.13	0.1271
GO:0034235	GPI anchor binding	5	1	0.13	0.1271
GO:0016641	oxidoreductase activity, acting on the C...	5	1	0.13	0.1271
GO:0033764	steroid dehydrogenase activity, acting o...	5	1	0.13	0.1271
GO:0051920	peroxiredoxin activity	5	1	0.13	0.1271
GO:0005537	D-mannose binding	5	1	0.13	0.1271
GO:0004791	thioredoxin-disulfide reductase (NADPH) ...	5	1	0.13	0.1271
GO:0043047	single-stranded telomeric DNA binding	5	1	0.13	0.1271
GO:0004046	aminoacylase activity	5	1	0.13	0.1271
GO:0004448	isocitrate dehydrogenase [NAD(P)+] activ...	5	1	0.13	0.1271
GO:0098809	nitrite reductase activity	5	1	0.13	0.1271
GO:1990247	N6-methyladenosine-containing RNA reader...	5	1	0.13	0.1271
GO:0016746	acyltransferase activity	329	6	8.82	0.1301
GO:0016836	hydro-lyase activity	25	2	0.67	0.1437
GO:0047134	protein-disulfide reductase [NAD(P)H] ac...	11	2	0.29	0.1498

Table S10, related to Figure 3c. Top 50 GO Biological Process Enrichment for *T. ni* Cluster TN2

GO.ID	Term	Num in Genome	Num in Cluster	Expected	p-val
GO:0018279	protein N-linked glycosylation via aspar...	36	9	0.98	5.30E-07
GO:0006508	proteolysis	862	31	23.47	6.50E-07
GO:0006805	xenobiotic metabolic process	104	11	2.83	0.00015
GO:0019853	L-ascorbic acid biosynthetic process	18	4	0.49	0.00121
GO:0006636	unsaturated fatty acid biosynthetic proc...	50	6	1.36	0.00134
GO:2001301	lipoxin biosynthetic process	9	3	0.24	0.00148
GO:0046456	icosanoid biosynthetic process	29	4	0.79	0.00205
GO:0019372	lipoxygenase pathway	10	3	0.27	0.00207
GO:0006506	GPI anchor biosynthetic process	21	4	0.57	0.00221
GO:0051923	sulfation	11	3	0.3	0.00279
GO:0097070	ductus arteriosus closure	11	3	0.3	0.00279
GO:0070493	thrombin-activated receptor signaling pa...	13	3	0.35	0.00464
GO:0035722	interleukin-12-mediated signaling pathwa...	26	4	0.71	0.00497
GO:0006457	protein folding	140	9	3.81	0.00506
GO:0006693	prostaglandin metabolic process	62	6	1.69	0.00582
GO:0019673	GDP-mannose metabolic process	5	2	0.14	0.00698
GO:0000023	maltose metabolic process	5	2	0.14	0.00698
GO:0034356	NAD biosynthesis via nicotinamide ribosi...	5	2	0.14	0.00698
GO:0016093	polyprenol metabolic process	5	2	0.14	0.00698
GO:0016320	endoplasmic reticulum membrane fusion	5	2	0.14	0.00698
GO:0015677	copper ion import	5	2	0.14	0.00698
GO:0006979	response to oxidative stress	332	15	9.04	0.00739
GO:0007586	digestion	104	6	2.83	0.00847
GO:0007567	parturition	16	3	0.44	0.00856
GO:0019430	removal of superoxide radicals	23	4	0.63	0.01012
GO:0008202	steroid metabolic process	193	8	5.25	0.01018
GO:0045454	cell redox homeostasis	17	3	0.46	0.01018
GO:0006691	leukotriene metabolic process	6	2	0.16	0.01029
GO:0046487	glyoxylate metabolic process	6	2	0.16	0.01029
GO:0070813	hydrogen sulfide metabolic process	6	2	0.16	0.01029
GO:0008291	acetylcholine metabolic process	6	2	0.16	0.01029
GO:0006006	glucose metabolic process	119	7	3.24	0.0104
GO:0001822	kidney development	154	7	4.19	0.01049
GO:1901605	alpha-amino acid metabolic process	144	6	3.92	0.0141
GO:0010269	response to selenium ion	7	2	0.19	0.01414
GO:1904707	positive regulation of vascular associat...	20	3	0.54	0.01608
GO:0034976	response to endoplasmic reticulum stress	190	8	5.17	0.01663
GO:0042759	long-chain fatty acid biosynthetic proce...	22	3	0.6	0.02088
GO:0009404	toxin metabolic process	19	4	0.52	0.02298
GO:0046680	response to DDT	9	2	0.24	0.02339
GO:0035269	protein O-linked mannosylation	9	2	0.24	0.02339
GO:0002790	peptide secretion	267	10	7.27	0.02493
GO:0034767	positive regulation of monoatomic ion tr...	73	5	1.99	0.02691
GO:0005976	polysaccharide metabolic process	70	4	1.91	0.02705
GO:0042278	purine nucleoside metabolic process	43	2	1.17	0.02724
GO:0009205	purine ribonucleoside triphosphate metab...	184	4	5.01	0.02753
GO:0018212	peptidyl-tyrosine modification	122	2	3.32	0.02757
GO:1901568	fatty acid derivative metabolic process	117	12	3.18	0.02773
GO:0044720	negative regulation of imaginal disc-der...	10	2	0.27	0.02873
GO:0046949	fatty-acyl-CoA biosynthetic process	33	4	0.9	0.02907

Table S11, related to Figure 3d. Top 50 GO Molecular Function Enrichment for *T. ni* Cluster TN3

GO.ID	Term	Num in Genome	Num in Cluster	Expected	p-val
GO:0001872	(1->3)-beta-D-glucan binding	7	5	0.23	6.60E-07
GO:0016742	hydroxymethyl-, formyl- and related tran...	7	5	0.23	6.60E-07
GO:0004252	serine-type endopeptidase activity	106	15	3.42	1.20E-06
GO:0038187	pattern recognition receptor activity	12	5	0.39	2.20E-05
GO:0001530	lipopolysaccharide binding	13	5	0.42	3.40E-05
GO:0030170	pyridoxal phosphate binding	18	5	0.58	0.0002
GO:0005504	fatty acid binding	41	7	1.32	0.00054
GO:0016701	oxidoreductase activity, acting on singl...	11	3	0.35	0.00103
GO:0042803	protein homodimerization activity	521	29	16.8	0.00233
GO:0070402	NADPH binding	9	3	0.29	0.0024
GO:0016646	oxidoreductase activity, acting on the C...	11	3	0.35	0.0045
GO:0019842	vitamin binding	35	9	1.13	0.00541
GO:0016638	oxidoreductase activity, acting on the C...	9	4	0.29	0.00583
GO:0008483	transaminase activity	12	3	0.39	0.00585
GO:0033791	3alpha,7alpha,12alpha-trihydroxy-5beta-c...	5	2	0.16	0.0097
GO:0005041	low-density lipoprotein particle recepto...	5	2	0.16	0.0097
GO:0016500	protein-hormone receptor activity	5	2	0.16	0.0097
GO:0043237	laminin-1 binding	5	2	0.16	0.0097
GO:0016401	palmitoyl-CoA oxidase activity	5	2	0.16	0.0097
GO:0016641	oxidoreductase activity, acting on the C...	5	2	0.16	0.0097
GO:0003995	acyl-CoA dehydrogenase activity	15	3	0.48	0.01127
GO:0008017	microtubule binding	132	10	4.26	0.01398
GO:0003988	acetyl-CoA C-acyltransferase activity	6	2	0.19	0.01424
GO:0016885	ligase activity, forming carbon-carbon b...	6	2	0.19	0.01424
GO:0016402	pristanoyl-CoA oxidase activity	6	2	0.19	0.01424
GO:0016832	aldehyde-lyase activity	6	2	0.19	0.01424
GO:0043395	heparan sulfate proteoglycan binding	17	3	0.55	0.01607
GO:0016879	ligase activity, forming carbon-nitrogen...	32	4	1.03	0.01859
GO:0034185	apolipoprotein binding	7	2	0.23	0.01952
GO:0042169	SH2 domain binding	19	3	0.61	0.02185
GO:0004033	aldo-keto reductase (NADPH) activity	18	3	0.58	0.0253
GO:0030371	translation repressor activity	24	3	0.77	0.02535
GO:0070325	lipoprotein particle receptor binding	19	3	0.61	0.032
GO:0004667	prostaglandin-D synthase activity	9	2	0.29	0.03207
GO:1901681	sulfur compound binding	89	8	2.87	0.03903
GO:0042834	peptidoglycan binding	10	2	0.32	0.03925
GO:0005212	structural constituent of eye lens	10	2	0.32	0.03925
GO:0005518	collagen binding	24	3	0.77	0.04059
GO:0032050	clathrin heavy chain binding	11	2	0.35	0.04697
GO:0004714	transmembrane receptor protein tyrosine ...	11	2	0.35	0.04697
GO:0044389	ubiquitin-like protein ligase binding	185	5	5.96	0.04818
GO:0016830	carbon-carbon lyase activity	32	5	1.03	0.04861
GO:0005496	steroid binding	53	4	1.71	0.04969
GO:0008374	O-acyltransferase activity	25	3	0.81	0.05489
GO:0004888	transmembrane signaling receptor activit...	145	12	4.68	0.06275
GO:0033612	receptor serine/threonine kinase binding	9	2	0.29	0.06319
GO:0001882	nucleoside binding	106	4	3.42	0.06357
GO:0016597	amino acid binding	36	4	1.16	0.06462
GO:0000287	magnesium ion binding	89	6	2.87	0.06664
GO:0004497	monooxygenase activity	30	3	0.97	0.07085

Table S12, related to Figure 3d. Top 50 GO Biological Process Enrichment for *T. ni* Cluster TN3

GO.ID	Term	Num in Genome	Num in Cluster	Expected	p-val
GO:0042866	pyruvate biosynthetic process	27	7	0.85	1.40E-05
GO:0006508	proteolysis	862	42	27.1	3.40E-05
GO:0006723	cuticle hydrocarbon biosynthetic process	9	4	0.28	0.00011
GO:0019359	nicotinamide nucleotide biosynthetic pro...	38	7	1.19	0.00017
GO:0008652	amino acid biosynthetic process	46	10	1.45	0.00024
GO:0170044	non-proteinogenic amino acid catabolic p...	11	4	0.35	0.00026
GO:0045138	nematode male tail tip morphogenesis	42	7	1.32	0.00029
GO:0046654	tetrahydrofolate biosynthetic process	5	3	0.16	0.00029
GO:0006546	glycine catabolic process	5	3	0.16	0.00029
GO:0032491	detection of molecule of fungal origin	5	3	0.16	0.00029
GO:0060976	coronary vasculature development	35	6	1.1	0.0003
GO:0019432	triglyceride biosynthetic process	42	6	1.32	0.00038
GO:0009113	purine nucleobase biosynthetic process	11	4	0.35	0.00056
GO:0006545	glycine biosynthetic process	6	3	0.19	0.00057
GO:0019388	galactose catabolic process	6	3	0.19	0.00057
GO:0061620	glycolytic process through glucose-6-pho...	15	3	0.47	0.00098
GO:0009068	aspartate family amino acid catabolic pr...	16	4	0.5	0.00128
GO:0006096	glycolytic process	44	7	1.38	0.00147
GO:0071329	cellular response to sucrose stimulus	8	3	0.25	0.00153
GO:0051607	defense response to virus	91	8	2.86	0.00205
GO:0006754	ATP biosynthetic process	74	7	2.33	0.00207
GO:0070233	negative regulation of T cell apoptotic ...	9	3	0.28	0.00224
GO:0051256	mitotic spindle midzone assembly	9	3	0.28	0.00224
GO:0045752	positive regulation of Toll signaling pa...	10	3	0.31	0.00312
GO:0008356	asymmetric cell division	127	11	3.99	0.00317
GO:0006084	acetyl-CoA metabolic process	21	4	0.66	0.00373
GO:0006082	organic acid metabolic process	663	54	20.84	0.00396
GO:0007419	ventral cord development	35	5	1.1	0.0044
GO:0055082	intracellular chemical homeostasis	345	5	10.84	0.00471
GO:0034404	nucleobase-containing small molecule bio...	82	8	2.58	0.00485
GO:0007446	imaginal disc growth	39	4	1.23	0.00545
GO:0009168	purine ribonucleoside monophosphate bios...	65	9	2.04	0.00566
GO:1905902	regulation of mesoderm formation	13	3	0.41	0.00693
GO:0030953	astral microtubule organization	13	3	0.41	0.00693
GO:0042661	regulation of mesodermal cell fate speci...	13	3	0.41	0.00693
GO:0003281	ventricular septum development	39	5	1.23	0.00849
GO:0019682	glyceraldehyde-3-phosphate metabolic pro...	14	3	0.44	0.00862
GO:0006544	glycine metabolic process	16	8	0.5	0.00876
GO:0046653	tetrahydrofolate metabolic process	10	5	0.31	0.00899
GO:0046439	L-cysteine metabolic process	11	3	0.35	0.00917
GO:0030706	germarium-derived oocyte differentiation	32	3	1.01	0.00922
GO:0033353	S-adenosylmethionine cycle	5	2	0.16	0.00924
GO:0010642	negative regulation of platelet-derived ...	5	2	0.16	0.00924
GO:0034196	acylglycerol transport	5	2	0.16	0.00924
GO:0006000	fructose metabolic process	5	2	0.16	0.00924
GO:0046415	urate metabolic process	5	2	0.16	0.00924
GO:0035791	platelet-derived growth factor receptor-...	5	2	0.16	0.00924
GO:0072350	tricarboxylic acid metabolic process	41	3	1.29	0.00925
GO:0042632	cholesterol homeostasis	42	5	1.32	0.00966
GO:0065008	regulation of biological quality	2074	73	65.19	0.01025

Table S13, related to Figure 4b. Top 50 GO Molecular Function Enrichment for *S. exigua* Ovary Repressed Cluster

GO.ID	Term	Num in Genome	Num in Cluster	Expected	p-val
GO:0043395	heparan sulfate proteoglycan binding	19	7	0.58	8.50E-07
GO:0016638	oxidoreductase activity, acting on the C...	10	4	0.31	0.00027
GO:0004341	gluconolactonase activity	5	3	0.15	0.00027
GO:0005041	low-density lipoprotein particle recepto...	6	3	0.18	0.00053
GO:0043168	anion binding	754	35	23.17	0.00058
GO:0005319	lipid transporter activity	57	9	1.75	0.0014
GO:0015651	quaternary ammonium group transmembrane ...	8	3	0.25	0.00143
GO:0005201	extracellular matrix structural constitu...	19	4	0.58	0.00233
GO:0001530	lipopolysaccharide binding	11	3	0.34	0.00393
GO:0019842	vitamin binding	47	5	1.44	0.00642
GO:0043548	phosphatidylinositol 3-kinase binding	21	4	0.65	0.00643
GO:0016303	1-phosphatidylinositol-3-kinase activity	13	3	0.4	0.00651
GO:0008519	ammonium channel activity	25	4	0.77	0.00659
GO:0004430	1-phosphatidylinositol 4-kinase activity	5	2	0.15	0.00883
GO:0005044	scavenger receptor activity	5	2	0.15	0.00883
GO:0022883	zinc efflux transmembrane transporter ac...	5	2	0.15	0.00883
GO:0070700	BMP receptor binding	5	2	0.15	0.00883
GO:0015234	thiamine transmembrane transporter activ...	5	2	0.15	0.00883
GO:0008201	heparin binding	29	4	0.89	0.01124
GO:0034185	apolipoprotein binding	6	2	0.18	0.01299
GO:0005518	collagen binding	32	4	0.98	0.01585
GO:0008198	ferrous iron binding	18	3	0.55	0.01658
GO:0008138	protein tyrosine/serine/threonine phosph...	18	3	0.55	0.01658
GO:0005509	calcium ion binding	151	10	4.64	0.01749
GO:0017153	sodium:dicarboxylate symporter activity	7	2	0.22	0.01781
GO:0008195	phosphatidate phosphatase activity	7	2	0.22	0.01781
GO:0038187	pattern recognition receptor activity	7	2	0.22	0.01781
GO:0004035	alkaline phosphatase activity	7	2	0.22	0.01781
GO:0005275	amine transmembrane transporter activity	7	2	0.22	0.01781
GO:0043237	laminin-1 binding	7	2	0.22	0.01781
GO:0008514	organic anion transmembrane transporter ...	154	9	4.73	0.02063
GO:0002020	protease binding	71	6	2.18	0.02133
GO:0043394	proteoglycan binding	32	9	0.98	0.02188
GO:0042834	peptidoglycan binding	8	2	0.25	0.02328
GO:0001968	fibronectin binding	8	2	0.25	0.02328
GO:0008061	chitin binding	22	3	0.68	0.02861
GO:0008324	monoatomic cation transmembrane transpor...	321	11	9.86	0.02905
GO:0034041	ABC-type sterol transporter activity	9	2	0.28	0.02933
GO:0005160	transforming growth factor beta receptor...	9	2	0.28	0.02933
GO:0120014	phospholipid transfer activity	9	2	0.28	0.02933
GO:0016623	oxidoreductase activity, acting on the a...	9	2	0.28	0.03061
GO:0001882	nucleoside binding	107	3	3.29	0.03093
GO:0019902	phosphatase binding	119	6	3.66	0.03215
GO:0016491	oxidoreductase activity	444	27	13.64	0.03513
GO:0004029	aldehyde dehydrogenase (NAD+) activity	10	2	0.31	0.03593
GO:0005125	cytokine activity	10	2	0.31	0.03593
GO:0004252	serine-type endopeptidase activity	60	5	1.84	0.03632
GO:0008270	zinc ion binding	271	14	8.33	0.03875
GO:0015220	choline transmembrane transporter activi...	12	2	0.37	0.05063
GO:0005506	iron ion binding	46	6	1.41	0.05164

Table S14, related to Figure 4b. Top 50 GO Biological Process Enrichment for *S. exigua* Ovary Repressed Cluster

GO.ID	Term	Num in Genome	Num in Cluster	Expected	p-val
GO:0016201	synaptic target inhibition	14	5	0.42	3.70E-05
GO:0071711	basement membrane organization	35	7	1.05	0.00012
GO:0090256	regulation of cell proliferation involve...	5	3	0.15	0.00025
GO:1901671	positive regulation of superoxide dismut...	5	3	0.15	0.00025
GO:1902548	negative regulation of cellular response...	5	3	0.15	0.00025
GO:1904833	positive regulation of removal of supero...	5	3	0.15	0.00025
GO:1903625	negative regulation of DNA catabolic pro...	5	3	0.15	0.00025
GO:1903611	negative regulation of calcium-dependent...	5	3	0.15	0.00025
GO:1901318	negative regulation of flagellated sperm...	5	3	0.15	0.00025
GO:1903052	positive regulation of proteolysis invol...	107	5	3.21	0.00026
GO:0019216	regulation of lipid metabolic process	255	20	7.65	0.00028
GO:0060100	positive regulation of phagocytosis, eng...	23	4	0.69	0.00049
GO:0042754	negative regulation of circadian rhythm	6	3	0.18	0.0005
GO:0097156	fasciculation of motor neuron axon	6	3	0.18	0.0005
GO:0035295	tube development	1025	50	30.74	0.00058
GO:0048738	cardiac muscle tissue development	123	8	3.69	0.00061
GO:0045723	positive regulation of fatty acid biosyn...	14	4	0.42	0.00062
GO:0044719	regulation of imaginal disc-derived wing...	25	5	0.75	0.00075
GO:0051344	negative regulation of cyclic-nucleotide...	7	3	0.21	0.00085
GO:1905165	regulation of lysosomal protein cataboli...	7	3	0.21	0.00085
GO:0060586	multicellular organismal-level iron ion ...	7	3	0.21	0.00085
GO:0061526	acetylcholine secretion	11	3	0.33	0.00089
GO:1903427	negative regulation of reactive oxygen s...	16	4	0.48	0.00108
GO:1900409	positive regulation of cellular response...	16	4	0.48	0.00108
GO:0003143	embryonic heart tube morphogenesis	58	5	1.74	0.00108
GO:1901896	positive regulation of ATPase-coupled ca...	8	3	0.24	0.00133
GO:0010875	positive regulation of cholesterol efflu...	8	3	0.24	0.00133
GO:0035001	dorsal trunk growth, open tracheal syste...	8	3	0.24	0.00133
GO:0015697	quaternary ammonium group transport	8	3	0.24	0.00133
GO:0009631	cold acclimation	17	4	0.51	0.00138
GO:0060429	epithelium development	1259	58	37.76	0.0014
GO:0010867	positive regulation of triglyceride bios...	18	4	0.54	0.00173
GO:0048266	behavioral response to pain	9	3	0.27	0.00195
GO:0045019	negative regulation of nitric oxide bios...	9	3	0.27	0.00195
GO:1901033	positive regulation of response to react...	9	3	0.27	0.00195
GO:0097164	ammonium ion metabolic process	85	10	2.55	0.0021
GO:0002752	cell surface pattern recognition recepto...	15	4	0.45	0.00259
GO:1900006	positive regulation of dendrite developm...	80	8	2.4	0.0026
GO:0034341	response to type II interferon	46	4	1.38	0.00262
GO:1905037	autophagosome organization	86	6	2.58	0.0027
GO:0019853	L-ascorbic acid biosynthetic process	10	3	0.3	0.00273
GO:0015711	organic anion transport	265	14	7.95	0.00306
GO:0034260	negative regulation of GTPase activity	35	5	1.05	0.00361
GO:0048015	phosphatidylinositol-mediated signaling	35	5	1.05	0.00361
GO:0090201	negative regulation of release of cytoch...	11	3	0.33	0.00367
GO:1903011	negative regulation of bone development	11	3	0.33	0.00367
GO:0006541	glutamine metabolic process	22	4	0.66	0.00376
GO:0009642	response to light intensity	12	3	0.36	0.00479
GO:0046879	hormone secretion	166	11	4.98	0.00496
GO:0015837	amine transport	59	5	1.77	0.0051

Table S15, related to Figure 4c. Top 50 GO Molecular Function Enrichment for *S. exigua* Ovary Induced Cluster

GO.ID	Term	Num in Genome	Num in Cluster	Expected	p-val
GO:0043015	gamma-tubulin binding	15	3	0.28	0.0025
GO:0003690	double-stranded DNA binding	373	10	7.03	0.0027
GO:0034979	NAD-dependent protein lysine deacetylase...	6	2	0.11	0.005
GO:0032407	MutSalpha complex binding	6	2	0.11	0.005
GO:0070181	small ribosomal subunit rRNA binding	7	2	0.13	0.007
GO:0032405	MutLalpha complex binding	7	2	0.13	0.007
GO:0032558	adenyl deoxyribonucleotide binding	7	2	0.13	0.007
GO:0003899	DNA-directed 5'-3' RNA polymerase activi...	23	3	0.43	0.0088
GO:0030515	snoRNA binding	23	3	0.43	0.0088
GO:0008296	3'-5'-DNA exonuclease activity	8	2	0.15	0.0092
GO:0009922	fatty acid elongase activity	8	2	0.15	0.0092
GO:0003735	structural constituent of ribosome	137	7	2.58	0.0145
GO:0003950	NAD+ poly-ADP-ribosyltransferase activit...	11	2	0.21	0.0173
GO:0004312	fatty acid synthase activity	13	2	0.25	0.024
GO:0008649	rRNA methyltransferase activity	13	2	0.25	0.024
GO:0008097	5S rRNA binding	14	2	0.26	0.0276
GO:0016811	hydrolase activity, acting on carbon-nit...	63	4	1.19	0.0305
GO:0008171	O-methyltransferase activity	15	2	0.28	0.0315
GO:0160215	deacylase activity	62	5	1.17	0.0364
GO:0004693	cyclin-dependent protein serine/threonin...	19	2	0.36	0.0489
GO:0033558	protein lysine deacetylase activity	30	2	0.57	0.0553
GO:0000175	3'-5'-RNA exonuclease activity	23	2	0.43	0.0689
GO:0140994	RNA polymerase II CTD heptapeptide repea...	11	2	0.21	0.0728
GO:0060089	molecular transducer activity	228	3	4.3	0.0747
GO:0008408	3'-5' exonuclease activity	36	5	0.68	0.0883
GO:0004540	RNA nuclease activity	81	4	1.53	0.0896
GO:0005289	high-affinity L-arginine transmembrane t...	5	1	0.09	0.0908
GO:0000064	L-ornithine transmembrane transporter ac...	5	1	0.09	0.0908
GO:0005229	intracellularly calcium-gated chloride c...	5	1	0.09	0.0908
GO:0016635	oxidoreductase activity, acting on the C...	5	1	0.09	0.0908
GO:0008430	selenium binding	5	1	0.09	0.0908
GO:0008379	thioredoxin peroxidase activity	5	1	0.09	0.0908
GO:0048039	ubiquinone binding	5	1	0.09	0.0908
GO:0005176	ErbB-2 class receptor binding	5	1	0.09	0.0908
GO:0016428	tRNA (cytidine-5-)-methyltransferase act...	5	1	0.09	0.0908
GO:0004526	ribonuclease P activity	5	1	0.09	0.0908
GO:0005292	high-affinity lysine transmembrane trans...	5	1	0.09	0.0908
GO:0002161	aminoacyl-tRNA editing activity	5	1	0.09	0.0908
GO:0005085	guanyl-nucleotide exchange factor activi...	129	5	2.43	0.0958
GO:0055106	ubiquitin-protein transferase regulator ...	6	1	0.11	0.1079
GO:0043023	ribosomal large subunit binding	6	1	0.11	0.1079
GO:1990226	histone methyltransferase binding	6	1	0.11	0.1079
GO:0047144	2-acylglycerol-3-phosphate O-acyltransfe...	6	1	0.11	0.1079
GO:0003747	translation release factor activity	6	1	0.11	0.1079
GO:0005096	GTPase activator activity	137	5	2.58	0.1159
GO:0016229	steroid dehydrogenase activity	7	1	0.13	0.1248
GO:0080019	alcohol-forming very long-chain fatty ac...	7	1	0.13	0.1248
GO:0008353	RNA polymerase II CTD heptapeptide repea...	7	1	0.13	0.1248
GO:0030620	U2 snRNA binding	7	1	0.13	0.1248
GO:0008559	ABC-type xenobiotic transporter activity	7	1	0.13	0.1248

Table S16, related to Figure 4c. Top 50 GO Biological Process Enrichment for *S. exigua* Ovary Induced Cluster

GO.ID	Term	Num in Genome	Num in Cluster	Expected	p-val
GO:0070126	mitochondrial translational termination	61	7	1.2	0.00018
GO:0007095	mitotic G2 DNA damage checkpoint signali...	22	4	0.43	0.0008
GO:0006488	dolichol-linked oligosaccharide biosynth...	13	3	0.26	0.00186
GO:0016093	polyprenol metabolic process	5	2	0.1	0.00371
GO:1902463	protein localization to cell leading edg...	5	2	0.1	0.00371
GO:0070125	mitochondrial translational elongation	58	5	1.14	0.00549
GO:0034625	fatty acid elongation, monounsaturated f...	8	2	0.16	0.00999
GO:0034626	fatty acid elongation, polyunsaturated f...	8	2	0.16	0.00999
GO:0019367	fatty acid elongation, saturated fatty a...	8	2	0.16	0.00999
GO:0031990	mRNA export from nucleus in response to ...	9	2	0.18	0.01268
GO:0045003	double-strand break repair via synthesis...	9	2	0.18	0.01268
GO:0035458	cellular response to interferon-beta	10	2	0.2	0.01565
GO:0045498	sex comb development	11	2	0.22	0.01889
GO:0042761	very long-chain fatty acid biosynthetic ...	11	2	0.22	0.01889
GO:1904811	positive regulation of dense core granul...	11	2	0.22	0.01889
GO:0061615	glycolytic process through fructose-6-ph...	20	2	0.39	0.01962
GO:0007113	endomitotic cell cycle	12	2	0.24	0.02237
GO:0090326	positive regulation of locomotion involv...	12	2	0.24	0.02237
GO:0006370	7-methylguanosine mRNA capping	32	3	0.63	0.0245
GO:0030866	cortical actin cytoskeleton organization	97	5	1.91	0.0254
GO:0032790	ribosome disassembly	13	2	0.26	0.0261
GO:0016973	poly(A)+ mRNA export from nucleus	13	2	0.26	0.0261
GO:0000288	nuclear-transcribed mRNA catabolic proce...	61	4	1.2	0.03177
GO:0006283	transcription-coupled nucleotide-excisio...	62	4	1.22	0.03346
GO:0002230	positive regulation of defense response ...	15	2	0.3	0.03425
GO:0010667	negative regulation of cardiac muscle ce...	15	2	0.3	0.03425
GO:0031167	rRNA methylation	15	2	0.3	0.03425
GO:0006310	DNA recombination	167	6	3.29	0.03812
GO:0071028	nuclear mRNA surveillance	16	2	0.32	0.03865
GO:0097345	mitochondrial outer membrane permeabiliz...	15	2	0.3	0.03883
GO:0034389	lipid droplet organization	17	2	0.34	0.04325
GO:0051403	stress-activated MAPK cascade	181	4	3.57	0.045
GO:0009065	glutamine family amino acid catabolic pr...	19	2	0.37	0.05301
GO:0045786	negative regulation of cell cycle	350	14	6.9	0.05636
GO:0008535	respiratory chain complex IV assembly	20	2	0.39	0.05772
GO:0006363	termination of RNA polymerase I transcri...	20	2	0.39	0.05816
GO:0032479	regulation of type I interferon producti...	49	3	0.97	0.06311
GO:0031000	response to caffeine	21	2	0.41	0.06347
GO:0036270	response to diuretic	21	2	0.41	0.06347
GO:0034587	piRNA processing	21	2	0.41	0.06347
GO:0006695	cholesterol biosynthetic process	56	3	1.1	0.06865
GO:0006541	glutamine metabolic process	22	2	0.43	0.06894
GO:0006011	UDP-alpha-D-glucose metabolic process	22	2	0.43	0.06894
GO:0007254	JNK cascade	142	3	2.8	0.07013
GO:0045892	negative regulation of DNA-templated tra...	594	12	11.71	0.07131
GO:0070317	negative regulation of G0 to G1 transiti...	23	2	0.45	0.07456
GO:0006367	transcription initiation at RNA polymera...	164	6	3.23	0.07627
GO:0031345	negative regulation of cell projection o...	130	3	2.56	0.07683
GO:0030838	positive regulation of actin filament po...	51	3	1.01	0.07877
GO:0007167	enzyme-linked receptor protein signaling...	606	8	11.95	0.07939

Table S17, related to Figure 5b. Top 50 GO Molecular Function Enrichment for *T.ni* Ovary Repressed Cluster

GO.ID	Term	Num in Genome	Num in Cluster	Expected	p-val
GO:0000978	RNA polymerase II cis-regulatory region ...	170	63	33.5	2.60E-07
GO:0005178	integrin binding	44	24	8.67	2.80E-07
GO:0003779	actin binding	172	64	33.89	4.30E-06
GO:0042803	protein homodimerization activity	511	140	100.7	6.50E-06
GO:0043395	heparan sulfate proteoglycan binding	15	11	2.96	1.00E-05
GO:0008061	chitin binding	21	13	4.14	2.60E-05
GO:0019901	protein kinase binding	393	121	77.45	3.10E-05
GO:0004568	chitinase activity	12	9	2.36	5.40E-05
GO:0050839	cell adhesion molecule binding	108	51	21.28	9.10E-05
GO:0046982	protein heterodimerization activity	241	70	47.49	0.00024
GO:0015318	inorganic molecular entity transmembrane...	395	83	77.84	0.00025
GO:0008134	transcription factor binding	398	107	78.43	0.00032
GO:0031748	D1 dopamine receptor binding	7	6	1.38	0.00034
GO:0031267	small GTPase binding	269	76	53.01	0.00034
GO:0004888	transmembrane signaling receptor activit...	127	44	25.03	0.00044
GO:0003714	transcription corepressor activity	92	32	18.13	0.00046
GO:0001227	DNA-binding transcription repressor acti...	74	27	14.58	0.00053
GO:0001228	DNA-binding transcription activator acti...	125	40	24.63	0.00068
GO:0001046	core promoter sequence-specific DNA bind...	28	11	5.52	0.00069
GO:0034041	ABC-type sterol transporter activity	10	7	1.97	0.00078
GO:0043168	anion binding	699	176	137.75	0.00087
GO:0008201	heparin binding	28	13	5.52	0.00122
GO:0003988	acetyl-CoA C-acyltransferase activity	6	5	1.18	0.00148
GO:0051015	actin filament binding	75	26	14.78	0.00161
GO:0008509	monoatomic anion transmembrane transport...	187	53	36.85	0.00167
GO:0030215	semaphorin receptor binding	11	7	2.17	0.00177
GO:0050750	low-density lipoprotein particle recepto...	17	9	3.35	0.00228
GO:0044877	protein-containing complex binding	596	165	117.45	0.00231
GO:0045296	cadherin binding	30	13	5.91	0.00264
GO:0004867	serine-type endopeptidase inhibitor acti...	9	6	1.77	0.00281
GO:0005102	signaling receptor binding	508	160	100.11	0.00289
GO:0001540	amyloid-beta binding	27	12	5.32	0.00297
GO:0005518	collagen binding	24	11	4.73	0.00329
GO:0004683	calcium/calmodulin-dependent protein kin...	12	7	2.36	0.00354
GO:0004714	transmembrane receptor protein tyrosine ...	12	7	2.36	0.00354
GO:0015485	cholesterol binding	21	10	4.14	0.00358
GO:0004467	long-chain fatty acid-CoA ligase activit...	15	8	2.96	0.0038
GO:0003682	chromatin binding	314	85	61.88	0.00446
GO:0008013	beta-catenin binding	50	18	9.85	0.00515
GO:0050681	nuclear androgen receptor binding	32	13	6.31	0.00519
GO:0043565	sequence-specific DNA binding	347	111	68.38	0.00581
GO:0004869	cysteine-type endopeptidase inhibitor ac...	19	9	3.74	0.00593
GO:0048306	calcium-dependent protein binding	29	12	5.71	0.00601
GO:0046332	SMAD binding	41	15	8.08	0.00623
GO:0019955	cytokine binding	20	11	3.94	0.00629
GO:0042974	nuclear retinoic acid receptor binding	11	6	2.17	0.00631
GO:0005041	low-density lipoprotein particle recepto...	5	4	0.99	0.00633
GO:0005548	phospholipid transporter activity	20	9	3.94	0.00635
GO:0043027	cysteine-type endopeptidase inhibitor ac...	13	7	2.56	0.00637
GO:0045545	syndecan binding	8	5	1.58	0.0097

Table S18, related to Figure 5b. Top 50 GO Biological Process Enrichment for *T.ni* Ovary Repressed Cluster

GO.ID	Term	Num in Genome	Num in Cluster	Expected	p-val
GO:0032989	cellular anatomical entity morphogenesis	771	256	147	8.30E-24
GO:0060562	epithelial tube morphogenesis	501	189	95.52	1.20E-15
GO:0008045	motor neuron axon guidance	86	45	16.4	3.60E-12
GO:0051674	localization of cell	573	176	109.25	3.90E-12
GO:0002066	columnar/cuboidal epithelial cell develo...	267	92	50.91	2.80E-10
GO:0006935	chemotaxis	414	149	78.93	9.80E-10
GO:0045666	positive regulation of neuron differenti...	274	94	52.24	1.60E-09
GO:0007474	imaginal disc-derived wing vein specific...	43	26	8.2	2.20E-09
GO:0007298	border follicle cell migration	107	44	20.4	9.10E-08
GO:0050768	negative regulation of neurogenesis	197	72	37.56	1.20E-07
GO:0045944	positive regulation of transcription by ...	510	147	97.24	1.60E-07
GO:0046330	positive regulation of JNK cascade	66	31	12.58	2.20E-07
GO:0010628	positive regulation of gene expression	981	257	187.03	4.40E-07
GO:0001709	cell fate determination	130	51	24.79	6.90E-07
GO:0008586	imaginal disc-derived wing vein morphoge...	44	23	8.39	7.50E-07
GO:0001655	urogenital system development	198	74	37.75	8.00E-07
GO:0000122	negative regulation of transcription by ...	384	111	73.21	9.80E-07
GO:0010770	positive regulation of cell morphogenesi...	126	47	24.02	1.20E-06
GO:0010976	positive regulation of neuron projection...	228	81	43.47	1.20E-06
GO:0022617	extracellular matrix disassembly	26	19	4.96	2.30E-06
GO:0032874	positive regulation of stress-activated ...	76	32	14.49	2.80E-06
GO:0048813	dendrite morphogenesis	219	74	41.75	4.60E-06
GO:0032879	regulation of localization	1208	342	230.31	4.80E-06
GO:0007411	axon guidance	317	127	60.44	6.00E-06
GO:0007185	cell surface receptor protein tyrosine p...	7	7	1.33	9.00E-06
GO:0007413	axonal fasciculation	42	22	8.01	9.50E-06
GO:0001934	positive regulation of protein phosphory...	355	110	67.68	9.90E-06
GO:0051491	positive regulation of filopodium assemb...	44	21	8.39	1.50E-05
GO:1901653	cellular response to peptide	209	65	39.85	1.60E-05
GO:0001702	gastrulation with mouth forming second	26	16	4.96	1.80E-05
GO:0010888	negative regulation of lipid storage	21	12	4	2.00E-05
GO:0022409	positive regulation of cell-cell adhesio...	74	28	14.11	2.20E-05
GO:0007420	brain development	436	137	83.13	2.20E-05
GO:0043507	positive regulation of JUN kinase activi...	42	20	8.01	2.40E-05
GO:0007435	salivary gland morphogenesis	84	32	16.02	2.40E-05
GO:0010629	negative regulation of gene expression	1037	239	197.71	2.50E-05
GO:0044703	multi-organism reproductive process	1158	286	220.78	2.60E-05
GO:0060359	response to ammonium ion	80	31	15.25	2.60E-05
GO:0031290	retinal ganglion cell axon guidance	30	16	5.72	2.70E-05
GO:0007426	tracheal outgrowth, open tracheal system	19	12	3.62	2.90E-05
GO:0008284	positive regulation of cell population p...	342	110	65.2	3.00E-05
GO:0042220	response to cocaine	42	18	8.01	3.60E-05
GO:0050769	positive regulation of neurogenesis	311	109	59.29	3.90E-05
GO:0007265	Ras protein signal transduction	236	72	45	4.10E-05
GO:0060071	Wnt signaling pathway, planar cell polar...	34	18	6.48	4.10E-05
GO:0048052	R1/R6 cell differentiation	6	6	1.14	4.80E-05
GO:0010745	negative regulation of macrophage derive...	6	6	1.14	4.80E-05
GO:0060742	epithelial cell differentiation involved...	6	6	1.14	4.80E-05
GO:0046533	negative regulation of photoreceptor cel...	10	8	1.91	5.30E-05
GO:0030902	hindbrain development	83	27	15.82	5.80E-05

Table S19, related to Figure 5c. Top 50 GO Molecular Function Enrichment for *T.ni* Ovary Induced Cluster

GO.ID	Term	Num in Genome	Num in Cluster	Expected	p-val
GO:0003735	structural constituent of ribosome	132	46	18.32	6.30E-10
GO:0008137	NADH dehydrogenase (ubiquinone) activity	30	15	4.16	2.40E-06
GO:0050136	NADH:ubiquinone reductase (non-electroge...	30	15	4.16	2.40E-06
GO:0015450	protein-transporting ATPase activity	11	8	1.53	1.50E-05
GO:0003954	NADH dehydrogenase activity	38	21	5.27	0.00014
GO:1904680	peptide transmembrane transporter activi...	15	8	2.08	0.00035
GO:0000175	3'-5'-RNA exonuclease activity	25	11	3.47	0.0006
GO:0042887	amide transmembrane transporter activity	20	9	2.78	0.00073
GO:0030378	serine racemase activity	5	4	0.69	0.00164
GO:0008455	alpha-1,6-mannosylglycoprotein 2-beta-N-...	5	4	0.69	0.00164
GO:0003743	translation initiation factor activity	37	12	5.14	0.00312
GO:0003723	RNA binding	594	96	82.45	0.00427
GO:0008757	S-adenosylmethionine-dependent methyltra...	94	26	13.05	0.01004
GO:0016462	pyrophosphatase activity	473	51	65.66	0.01296
GO:0016018	cyclosporin A binding	8	4	1.11	0.01622
GO:0004550	nucleoside diphosphate kinase activity	8	4	1.11	0.01622
GO:0043024	ribosomal small subunit binding	12	5	1.67	0.01734
GO:0019843	rRNA binding	38	9	5.27	0.02084
GO:0031386	protein tag activity	5	3	0.69	0.02143
GO:0016433	rRNA (adenine) methyltransferase activit...	5	3	0.69	0.02143
GO:0034979	NAD-dependent protein lysine deacetylase...	5	3	0.69	0.02143
GO:0016417	S-acyltransferase activity	17	4	2.36	0.02148
GO:0043130	ubiquitin binding	52	13	7.22	0.02249
GO:0003899	DNA-directed 5'-3' RNA polymerase activi...	22	7	3.05	0.0249
GO:0008121	ubiquinol-cytochrome-c reductase activit...	9	4	1.25	0.02604
GO:0016842	amidine-lyase activity	6	3	0.83	0.03848
GO:0005347	ATP transmembrane transporter activity	6	3	0.83	0.03848
GO:0015217	ADP transmembrane transporter activity	6	3	0.83	0.03848
GO:0004526	ribonuclease P activity	6	3	0.83	0.03848
GO:0061631	ubiquitin conjugating enzyme activity	24	7	3.33	0.0395
GO:0003690	double-stranded DNA binding	337	27	46.78	0.04016
GO:0005507	copper ion binding	20	6	2.78	0.04859
GO:0000993	RNA polymerase II complex binding	20	6	2.78	0.04859
GO:0051087	protein-folding chaperone binding	78	14	10.83	0.04965
GO:0019209	kinase activator activity	42	7	5.83	0.05246
GO:0033558	protein lysine deacetylase activity	25	4	3.47	0.05254
GO:0046920	alpha-(1->3)-fucosyltransferase activity	11	4	1.53	0.05434
GO:0097602	cullin family protein binding	11	4	1.53	0.05434
GO:0008135	translation factor activity, RNA binding	60	18	8.33	0.05905
GO:0043022	ribosome binding	56	16	7.77	0.05994
GO:0003755	peptidyl-prolyl cis-trans isomerase acti...	21	6	2.91	0.06033
GO:0016846	carbon-sulfur lyase activity	7	3	0.97	0.06052
GO:0003950	NAD+ poly-ADP-ribosyltransferase activit...	7	3	0.97	0.06052
GO:0033842	N-acetyl-beta-glucosaminyl-derivative 4-...	7	3	0.97	0.06052
GO:0008170	N-methyltransferase activity	51	12	7.08	0.07212
GO:0022857	transmembrane transporter activity	523	83	72.6	0.07364
GO:0008175	tRNA methyltransferase activity	15	5	2.08	0.08683
GO:0004341	gluconolactonase activity	8	3	1.11	0.0871
GO:0008174	mRNA methyltransferase activity	8	3	1.11	0.0871
GO:0019788	NEDD8 transferase activity	8	3	1.11	0.0871

Table S20, related to Figure 5c. Top 50 GO Biological Process Enrichment for *T.ni* Ovary Induced Cluster

GO.ID	Term	Num in Genome	Num in Cluster	Expected	p-val
GO:0070126	mitochondrial translational termination	60	40	8.7	3.10E-20
GO:0070125	mitochondrial translational elongation	56	38	8.12	1.10E-19
GO:0032981	mitochondrial respiratory chain complex ...	45	25	6.53	1.40E-10
GO:0043043	peptide biosynthetic process	316	86	45.82	3.40E-10
GO:0043604	amide biosynthetic process	377	95	54.67	3.40E-09
GO:0006120	mitochondrial electron transport, NADH t...	41	22	5.95	4.40E-09
GO:0046034	ATP metabolic process	197	57	28.57	7.60E-09
GO:0009167	purine ribonucleoside monophosphate meta...	186	51	26.97	7.00E-08
GO:0065002	intracellular protein transmembrane tran...	39	17	5.66	8.60E-08
GO:0016310	phosphorylation	1002	136	145.3	9.20E-08
GO:0030150	protein import into mitochondrial matrix	14	11	2.03	1.40E-07
GO:0033108	mitochondrial respiratory chain complex ...	72	41	10.44	9.10E-06
GO:0000288	nuclear-transcribed mRNA catabolic proce...	59	19	8.56	3.20E-05
GO:0034475	U4 snRNA 3'-end processing	7	6	1.02	5.60E-05
GO:0007005	mitochondrion organization	352	111	51.04	6.10E-05
GO:0045039	protein insertion into mitochondrial inn...	5	5	0.73	6.40E-05
GO:0032543	mitochondrial translation	105	58	15.23	7.50E-05
GO:1990542	mitochondrial transmembrane transport	68	30	9.86	8.00E-05
GO:0044743	protein transmembrane import into intrac...	28	16	4.06	0.00042
GO:0001944	vasculature development	272	28	39.44	0.00125
GO:0033617	mitochondrial cytochrome c oxidase assem...	17	8	2.47	0.00136
GO:0006744	ubiquinone biosynthetic process	17	8	2.47	0.00136
GO:0042407	cristae formation	25	10	3.63	0.00161
GO:0000467	exonucleolytic trimming to generate matu...	5	4	0.73	0.00194
GO:0071035	nuclear polyadenylation-dependent rRNA c...	5	4	0.73	0.00194
GO:1905065	positive regulation of vascular associat...	5	4	0.73	0.00194
GO:0042776	proton motive force-driven mitochondrial...	18	8	2.61	0.00215
GO:0006783	heme biosynthetic process	15	7	2.18	0.00292
GO:0031365	N-terminal protein amino acid modificati...	21	7	3.05	0.0048
GO:0009249	protein lipoylation	6	4	0.87	0.00516
GO:0071051	poly(A)-dependent snoRNA 3'-end processi...	6	4	0.87	0.00516
GO:0071046	nuclear polyadenylation-dependent ncRNA ...	6	4	0.87	0.00516
GO:0043634	polyadenylation-dependent ncRNA cataboli...	6	4	0.87	0.00516
GO:0015985	energy coupled proton transport, down el...	25	9	3.63	0.00627
GO:0071028	nuclear mRNA surveillance	13	6	1.89	0.0063
GO:0016075	rRNA catabolic process	13	6	1.89	0.0063
GO:0006122	mitochondrial electron transport, ubiqui...	13	6	1.89	0.0063
GO:0017038	protein import	160	25	23.2	0.00755
GO:0006750	glutathione biosynthetic process	14	6	2.03	0.0097
GO:0050848	regulation of calcium-mediated signaling	53	11	7.69	0.0098
GO:0009117	nucleotide metabolic process	438	83	63.51	0.00997
GO:0009142	nucleoside triphosphate biosynthetic pro...	97	18	14.07	0.01063
GO:0008535	respiratory chain complex IV assembly	21	11	3.05	0.01066
GO:0070475	rRNA base methylation	7	4	1.02	0.01067
GO:0016078	tRNA decay	7	4	1.02	0.01067
GO:0071265	L-methionine biosynthetic process	9	5	1.31	0.0108
GO:0055070	copper ion homeostasis	13	5	1.89	0.01082
GO:0070071	proton-transporting two-sector ATPase co...	9	4	1.31	0.01084
GO:0006839	mitochondrial transport	147	52	21.32	0.0133
GO:0003351	epithelial cilium movement involved in e...	26	9	3.77	0.01345

Table S21, related to Figure 7. ATAC-seq Sequencing Library Information and Quality Scores

	Replicate	# Uniquely Mapped Reads	% Uniquely Mapped Reads	PBC1[1]	PBC2[2]	FRiP[3]
**EtOH Gen 1**	1	68,349,945	74.9	0.909	10.894	0.4349
	2	68,334,577	75.4	0.877	8.461	0.4795
	3	54,042,639	75.7	0.909	11.016	0.3802
**SFN Gen 1**	1	53,993,644	73.6	0.896	9.778	0.3868
	2	61,250,525	74.2	0.912	11.295	0.3901
	3	70,777,581	73.6	0.907	10.604	0.3979
**EtOH Gen 2**	1	57,505,325	74.3	0.903	10.195	0.3746
	2	59,920,257	76.2	0.901	9.973	0.3838
	3	50,328,755	78.2	0.887	8.998	0.4682
**SFN Gen 2**	1	99,791,759	77.3	0.882	8.539	0.4630
	2	77,275,583	75.1	0.909	10.809	0.4021
	3	74,517,967	74.7	0.897	9.690	0.4134
**Average**		**66,340,713**	**75**	**0.899**	**10.021**	**0.415**

[1] PBC1, PCR Bottleneck Coefficient 1 = genomic positions with one read / genomic positions with at least one read pair

[2] PBC2, PCR Bottleneck Coefficient 2 = positions with one read / positions with two reads

[3] FRiP, Fraction of Reads in Peaks = reads that overlap called peaks / total usable aligned reads

Table S22, related to Figure 7. Top 50 GO Molecular Function Enrichment for *S. exigua* Generation 2 Significantly Induced Peaks

GO.ID	Term	Num in Genome	Num in Cluster	Expected	p-val
GO:0008440	inositol-1,4,5-trisphosphate 3-kinase ac...	5	4	0.11	1.00E-06
GO:0004683	calcium/calmodulin-dependent protein kin...	18	4	0.39	0.00049
GO:0050840	extracellular matrix binding	22	4	0.47	0.00111
GO:0048306	calcium-dependent protein binding	45	5	0.97	0.00261
GO:0004467	long-chain fatty acid-CoA ligase activit...	15	3	0.32	0.00365
GO:0030145	manganese ion binding	34	4	0.73	0.00574
GO:0046975	histone H3K36 methyltransferase activity	6	2	0.13	0.0065
GO:0043014	alpha-tubulin binding	20	3	0.43	0.00847
GO:0005388	P-type calcium transporter activity	7	2	0.15	0.00897
GO:0031957	very long-chain fatty acid-CoA ligase ac...	7	2	0.15	0.00897
GO:0043395	heparan sulfate proteoglycan binding	22	3	0.47	0.01109
GO:0005516	calmodulin binding	92	6	1.98	0.0139
GO:0003755	peptidyl-prolyl cis-trans isomerase acti...	25	3	0.54	0.0158
GO:0035255	ionotropic glutamate receptor binding	26	3	0.56	0.01758
GO:0015245	fatty acid transmembrane transporter act...	10	2	0.21	0.01842
GO:0046570	methylthioribulose 1-phosphate dehydrata...	1	1	0.02	0.02149
GO:0004692	cGMP-dependent protein kinase activity	1	1	0.02	0.02149
GO:0047800	cysteamine dioxygenase activity	1	1	0.02	0.02149
GO:0004642	phosphoribosylformylglycinamidine syntha...	1	1	0.02	0.02149
GO:0050501	hyaluronan synthase activity	1	1	0.02	0.02149
GO:0004363	glutathione synthase activity	1	1	0.02	0.02149
GO:0008031	eclosion hormone activity	1	1	0.02	0.02149
GO:0004994	somatostatin receptor activity	1	1	0.02	0.02149
GO:0004133	glycogen debranching enzyme activity	1	1	0.02	0.02149
GO:0004134	4-alpha-glucanotransferase activity	1	1	0.02	0.02149
GO:0004135	amylo-alpha-1,6-glucosidase activity	1	1	0.02	0.02149
GO:0004512	inositol-3-phosphate synthase activity	1	1	0.02	0.02149
GO:0035375	zymogen binding	1	1	0.02	0.02149
GO:0004102	choline O-acetyltransferase activity	1	1	0.02	0.02149
GO:0030215	semaphorin receptor binding	11	2	0.24	0.0222
GO:0008083	growth factor activity	12	2	0.26	0.02627
GO:0004714	transmembrane receptor protein tyrosine ...	13	2	0.28	0.03062
GO:0000900	mRNA regulatory element binding translat...	14	2	0.3	0.03523
GO:0005328	neurotransmitter:sodium symporter activi...	14	2	0.3	0.03523
GO:0051392	tRNA N4-acetyltransferase activity	2	1	0.04	0.04251
GO:0032558	adenyl deoxyribonucleotide binding	2	1	0.04	0.04251
GO:0004415	hyalurononglucosaminidase activity	2	1	0.04	0.04251
GO:0042835	BRE binding	2	1	0.04	0.04251
GO:0019534	toxin transmembrane transporter activity	2	1	0.04	0.04251
GO:0019912	cyclin-dependent protein kinase activati...	2	1	0.04	0.04251
GO:0008261	allatostatin receptor activity	2	1	0.04	0.04251
GO:0051011	microtubule minus-end binding	2	1	0.04	0.04251
GO:1990883	18S rRNA cytidine N-acetyltransferase ac...	2	1	0.04	0.04251
GO:0072345	NAADP-sensitive calcium-release channel ...	2	1	0.04	0.04251
GO:0070182	DNA polymerase binding	17	2	0.37	0.05049
GO:0005509	calcium ion binding	157	7	3.37	0.05175
GO:0008143	poly(A) binding	18	2	0.39	0.05602
GO:0000016	lactase activity	18	2	0.39	0.05602
GO:0030515	snoRNA binding	24	3	0.52	0.06106
GO:0008422	beta-glucosidase activity	19	2	0.41	0.06175

Table S23, related to Figure 7. Top 50 GO Biological Process Enrichment for *S. exigua* Generation 2 Significantly Induced Peaks

GO.ID	Term	Num in Genome	Num in Cluster	Expected	p-val
GO:0006020	inositol metabolic process	7	5	0.15	2.70E-06
GO:0032958	inositol phosphate biosynthetic process	11	5	0.23	2.70E-06
GO:0032957	inositol trisphosphate metabolic process	11	4	0.23	5.50E-05
GO:0061003	positive regulation of dendritic spine m...	14	4	0.29	0.00016
GO:0097062	dendritic spine maintenance	15	4	0.32	0.00021
GO:0035441	cell migration involved in vasculogenesi...	2	2	0.04	0.00044
GO:0030036	actin cytoskeleton organization	454	15	9.54	0.00062
GO:1902850	microtubule cytoskeleton organization in...	184	7	3.87	0.00128
GO:0006298	mismatch repair	27	4	0.57	0.00226
GO:0045653	negative regulation of megakaryocyte dif...	4	2	0.08	0.00256
GO:0006468	protein phosphorylation	830	21	17.44	0.00347
GO:0018026	peptidyl-lysine monomethylation	5	2	0.11	0.00421
GO:0010793	regulation of mRNA export from nucleus	5	2	0.11	0.00421
GO:0001579	medium-chain fatty acid transport	5	2	0.11	0.00421
GO:1905634	regulation of protein localization to ch...	5	2	0.11	0.00421
GO:0048332	mesoderm morphogenesis	65	3	1.37	0.00422
GO:0007475	apposition of dorsal and ventral imagina...	17	3	0.36	0.00498
GO:0071493	cellular response to UV-B	6	2	0.13	0.00622
GO:0031652	positive regulation of heat generation	6	2	0.13	0.00622
GO:0006659	phosphatidylserine biosynthetic process	6	2	0.13	0.00622
GO:0032727	positive regulation of interferon-alpha ...	6	2	0.13	0.00622
GO:0035070	salivary gland histolysis	36	4	0.76	0.00654
GO:0048102	autophagic cell death	36	4	0.76	0.00654
GO:0040027	negative regulation of vulval developmen...	19	3	0.4	0.00688
GO:0071277	cellular response to calcium ion	37	4	0.78	0.00722
GO:0032049	cardiolipin biosynthetic process	7	2	0.15	0.00859
GO:0071072	negative regulation of phospholipid bios...	7	2	0.15	0.00859
GO:0051443	positive regulation of ubiquitin-protein...	22	3	0.46	0.0112
GO:1904706	negative regulation of vascular associat...	8	2	0.17	0.0113
GO:0045752	positive regulation of Toll signaling pa...	8	2	0.17	0.0113
GO:0033211	adiponectin-activated signaling pathway	8	2	0.17	0.0113
GO:0040010	positive regulation of growth rate	23	3	0.48	0.01183
GO:0060173	limb development	96	4	2.02	0.01344
GO:0018027	peptidyl-lysine dimethylation	9	2	0.19	0.01433
GO:0042760	very long-chain fatty acid catabolic pro...	9	2	0.19	0.01433
GO:1903862	positive regulation of oxidative phospho...	9	2	0.19	0.01433
GO:0006541	glutamine metabolic process	25	3	0.53	0.0149
GO:0000413	protein peptidyl-prolyl isomerization	25	3	0.53	0.0149
GO:0001654	eye development	549	13	11.53	0.01517
GO:0007416	synapse assembly	264	9	5.55	0.01642
GO:0060669	embryonic placenta morphogenesis	10	2	0.21	0.01767
GO:0002804	positive regulation of antifungal peptid...	10	2	0.21	0.01767
GO:0045451	pole plasm oskar mRNA localization	57	4	1.2	0.01827
GO:0006656	phosphatidylcholine biosynthetic process	27	3	0.57	0.01839
GO:1905269	positive regulation of chromatin organiz...	101	6	2.12	0.01926
GO:1900006	positive regulation of dendrite developm...	74	5	1.55	0.01934
GO:0006520	amino acid metabolic process	225	8	4.73	0.0209
GO:2000224	regulation of testosterone biosynthetic ...	1	1	0.02	0.02101
GO:0008628	hormone-mediated apoptotic signaling pat...	1	1	0.02	0.02101
GO:0072675	osteoclast fusion	1	1	0.02	0.02101

Table S24, related to Figure 7. Top 50 GO Molecular Function Enrichment for *S. exigua* Generation 2 Significantly Repressed Peaks

GO.ID	Term	Num in Genome	Num in Cluster	Expected	p-val
GO:0004370	glycerol kinase activity	6	3	0.06	2.20E-05
GO:0036461	BLOC-2 complex binding	2	2	0.02	0.00011
GO:0036408	histone H3K14 acetyltransferase activity	2	2	0.02	0.00011
GO:0035651	AP-3 adaptor complex binding	3	2	0.03	0.00033
GO:0000979	RNA polymerase II core promoter sequence...	17	3	0.18	0.00069
GO:0035650	AP-1 adaptor complex binding	6	2	0.06	0.00161
GO:0061631	ubiquitin conjugating enzyme activity	24	3	0.25	0.00196
GO:0016174	NAD(P)H oxidase H2O2-forming activity	7	2	0.07	0.00224
GO:0008559	ABC-type xenobiotic transporter activity	8	2	0.08	0.00297
GO:0004185	serine-type carboxypeptidase activity	12	2	0.13	0.00681
GO:0010485	histone H4 acetyltransferase activity	12	2	0.13	0.00681
GO:0030742	GTP-dependent protein binding	14	2	0.15	0.00926
GO:0070577	lysine-acetylated histone binding	14	2	0.15	0.00926
GO:0004801	transaldolase activity	1	1	0.01	0.01059
GO:0070733	AMPylase activity	1	1	0.01	0.01059
GO:0005150	interleukin-1, type I receptor binding	1	1	0.01	0.01059
GO:0106026	Gly-tRNA(Ala) hydrolase activity	1	1	0.01	0.01059
GO:0050561	glutamate-tRNA(Gln) ligase activity	1	1	0.01	0.01059
GO:0048030	disaccharide binding	1	1	0.01	0.01059
GO:0051500	D-tyrosyl-tRNA(Tyr) deacylase activity	1	1	0.01	0.01059
GO:0044603	protein adenylylhydrolase activity	1	1	0.01	0.01059
GO:0004502	kynurenine 3-monooxygenase activity	1	1	0.01	0.01059
GO:0004692	cGMP-dependent protein kinase activity	1	1	0.01	0.01059
GO:0043539	protein serine/threonine kinase activato...	16	2	0.17	0.01205
GO:0015562	efflux transmembrane transporter activit...	18	2	0.19	0.01516
GO:0017025	TBP-class protein binding	19	2	0.2	0.01683
GO:0071949	FAD binding	20	2	0.21	0.01857
GO:0032558	adenyl deoxyribonucleotide binding	2	1	0.02	0.02107
GO:0035035	histone acetyltransferase binding	26	2	0.28	0.0305
GO:0018833	DDT-dehydrochlorinase activity	3	1	0.03	0.03144
GO:0004308	exo-alpha-sialidase activity	3	1	0.03	0.03144
GO:0032407	MutSalpha complex binding	3	1	0.03	0.03144
GO:0004557	alpha-galactosidase activity	3	1	0.03	0.03144
GO:0003863	3-methyl-2-oxobutanoate dehydrogenase (2...	3	1	0.03	0.03144
GO:0005534	galactose binding	3	1	0.03	0.03144
GO:0015250	water channel activity	3	1	0.03	0.03144
GO:0004818	glutamate-tRNA ligase activity	3	1	0.03	0.03144
GO:0001096	TFIIF-class transcription factor complex...	3	1	0.03	0.03144
GO:0032405	MutLalpha complex binding	4	1	0.04	0.0417
GO:0004174	electron-transferring-flavoprotein dehyd...	4	1	0.04	0.0417
GO:0005519	cytoskeletal regulatory protein binding	4	1	0.04	0.0417
GO:0035252	UDP-xylosyltransferase activity	4	1	0.04	0.0417
GO:0035091	phosphatidylinositol binding	131	3	1.39	0.04225
GO:0030145	manganese ion binding	34	2	0.36	0.04988
GO:0008670	2,4-dienoyl-CoA reductase (NADPH) activi...	5	1	0.05	0.05185
GO:0070016	armadillo repeat domain binding	5	1	0.05	0.05185
GO:0048273	mitogen-activated protein kinase p38 bin...	5	1	0.05	0.05185
GO:0045295	gamma-catenin binding	5	1	0.05	0.05185
GO:0008296	3'-5'-DNA exonuclease activity	6	1	0.06	0.0619
GO:0035325	Toll-like receptor binding	6	1	0.06	0.0619

Table S25, related to Figure 7. Top 50 GO Biological Process Enrichment for *S. exigua* Generation 2 Significantly Repressed Peaks

GO.ID	Term	Num in Genome	Num in Cluster	Expected	p-val
GO:0046167	glycerol-3-phosphate biosynthetic proces...	6	3	0.07	2.60E-05
GO:1905524	negative regulation of protein autoubiqu...	2	2	0.02	0.00012
GO:0060155	platelet dense granule organization	3	2	0.03	0.00036
GO:0090383	phagosome acidification	3	2	0.03	0.00036
GO:1903232	melanosome assembly	3	2	0.03	0.00036
GO:0006071	glycerol metabolic process	14	3	0.16	0.00044
GO:0009409	response to cold	52	5	0.58	0.00055
GO:2000825	positive regulation of androgen receptor...	4	2	0.04	0.00072
GO:1903026	negative regulation of RNA polymerase II...	4	2	0.04	0.00072
GO:0019432	triglyceride biosynthetic process	36	4	0.4	0.00078
GO:0006727	ommochrome biosynthetic process	19	3	0.21	0.00113
GO:0060261	positive regulation of transcription ini...	17	3	0.19	0.00117
GO:0035646	endosome to melanosome transport	5	2	0.06	0.00119
GO:0048072	compound eye pigmentation	20	3	0.22	0.00132
GO:0006856	eye pigment precursor transport	6	2	0.07	0.00178
GO:0048613	embryonic ectodermal digestive tract mor...	7	2	0.08	0.00247
GO:0071318	cellular response to ATP	7	2	0.08	0.00247
GO:0051123	RNA polymerase II preinitiation complex ...	22	3	0.24	0.00321
GO:0008057	eye pigment granule organization	9	2	0.1	0.00418
GO:1901142	insulin metabolic process	9	2	0.1	0.00418
GO:0048036	central complex development	9	2	0.1	0.00418
GO:0019217	regulation of fatty acid metabolic proce...	64	4	0.71	0.00465
GO:0031643	positive regulation of myelination	10	2	0.11	0.00518
GO:0044344	cellular response to fibroblast growth f...	73	4	0.81	0.00609
GO:0009786	regulation of asymmetric cell division	11	2	0.12	0.00629
GO:0030901	midbrain development	36	3	0.4	0.00726
GO:0045471	response to ethanol	212	7	2.36	0.00899
GO:0046677	response to antibiotic	333	8	3.7	0.00963
GO:0009636	response to toxic substance	484	12	5.38	0.01009
GO:0007040	lysosome organization	53	4	0.59	0.01011
GO:0051497	negative regulation of stress fiber asse...	14	2	0.16	0.01018
GO:0003309	type B pancreatic cell differentiation	23	2	0.26	0.01101
GO:0044602	protein deadenylylation	1	1	0.01	0.01111
GO:2000563	positive regulation of CD4-positive, alp...	1	1	0.01	0.01111
GO:1904937	sensory neuron migration	1	1	0.01	0.01111
GO:0014050	negative regulation of glutamate secreti...	1	1	0.01	0.01111
GO:0018117	protein adenylylation	1	1	0.01	0.01111
GO:0021693	cerebellar Purkinje cell layer structura...	1	1	0.01	0.01111
GO:0097102	endothelial tip cell fate specification	1	1	0.01	0.01111
GO:0021688	cerebellar molecular layer formation	1	1	0.01	0.01111
GO:2001190	positive regulation of T cell activation...	1	1	0.01	0.01111
GO:0008056	ocellus development	1	1	0.01	0.01111
GO:0150020	basal dendrite arborization	1	1	0.01	0.01111
GO:0061853	regulation of neuroblast migration	1	1	0.01	0.01111
GO:2001200	positive regulation of dendritic cell di...	1	1	0.01	0.01111
GO:0002519	natural killer cell tolerance induction	1	1	0.01	0.01111
GO:0032733	positive regulation of interleukin-10 pr...	1	1	0.01	0.01111
GO:0032736	positive regulation of interleukin-13 pr...	1	1	0.01	0.01111
GO:0021917	somatic motor neuron fate commitment	1	1	0.01	0.01111
GO:1902608	positive regulation of large conductance...	1	1	0.01	0.01111

Table S26, related to Figures 8e and 8f. Top 50 GO Molecular Function Enrichment for Putative *S. exigua* Induced Epialleles

GO.ID	Term	Num in Genome	Num in Cluster	Expected	p-val
GO:0005214	structural constituent of chitin-based c...	66	6	0.93	5.00E-05
GO:0005121	Toll binding	7	3	0.1	9.20E-05
GO:0008502	melatonin receptor activity	2	2	0.03	0.0002
GO:0004222	metalloendopeptidase activity	43	5	0.61	0.00032
GO:0080019	alcohol-forming very long-chain fatty ac...	11	3	0.16	0.00042
GO:0008142	oxysterol binding	3	2	0.04	0.00059
GO:0004181	metallocarboxypeptidase activity	33	4	0.47	0.00112
GO:0050660	flavin adenine dinucleotide binding	54	5	0.76	0.00122
GO:0047875	ecdysone oxidase activity	6	2	0.08	0.00286
GO:0004879	nuclear receptor activity	26	3	0.37	0.00563
GO:0004252	serine-type endopeptidase activity	118	6	1.67	0.00631
GO:0004745	all-trans-retinol dehydrogenase (NAD+) a...	9	2	0.13	0.00668
GO:0004732	pyridoxal oxidase activity	11	2	0.16	0.01001
GO:0017080	sodium channel regulator activity	11	2	0.16	0.01001
GO:0004854	xanthine dehydrogenase activity	13	2	0.18	0.01394
GO:0008083	growth factor activity	13	2	0.18	0.01394
GO:0004342	glucosamine-6-phosphate deaminase activi...	1	1	0.01	0.01414
GO:0010436	carotenoid dioxygenase activity	1	1	0.01	0.01414
GO:0072582	17-beta-hydroxysteroid dehydrogenase (NA...	1	1	0.01	0.01414
GO:0019966	interleukin-1 binding	1	1	0.01	0.01414
GO:0043120	tumor necrosis factor binding	1	1	0.01	0.01414
GO:0019959	interleukin-8 binding	1	1	0.01	0.01414
GO:0048403	brain-derived neurotrophic factor bindin...	1	1	0.01	0.01414
GO:0003834	beta-carotene 15,15'-dioxygenase activit...	1	1	0.01	0.01414
GO:0047498	calcium-dependent phospholipase A2 activ...	1	1	0.01	0.01414
GO:0005316	high-affinity phosphate:sodium symporter...	1	1	0.01	0.01414
GO:0020037	heme binding	39	3	0.55	0.01733
GO:0001222	transcription corepressor binding	15	2	0.21	0.01843
GO:0001223	transcription coactivator binding	17	2	0.24	0.02344
GO:0042392	sphingosine-1-phosphate phosphatase acti...	2	1	0.03	0.02808
GO:0048406	nerve growth factor binding	2	1	0.03	0.02808
GO:0004517	nitric-oxide synthase activity	2	1	0.03	0.02808
GO:0000253	3-beta-hydroxysteroid 3-dehydrogenase (N...	2	1	0.03	0.02808
GO:0019826	oxygen sensor activity	2	1	0.03	0.02808
GO:0046870	cadmium ion binding	2	1	0.03	0.02808
GO:0004838	L-tyrosine-2-oxoglutarate transaminase a...	2	1	0.03	0.02808
GO:0001588	dopamine neurotransmitter receptor activ...	2	1	0.03	0.02808
GO:0016298	lipase activity	92	5	1.3	0.03659
GO:0000978	RNA polymerase II cis-regulatory region ...	226	7	3.2	0.04033
GO:0034617	tetrahydrobiopterin binding	3	1	0.04	0.04182
GO:0034618	arginine binding	3	1	0.04	0.04182
GO:0016175	superoxide-generating NAD(P)H oxidase ac...	3	1	0.04	0.04182
GO:0016805	dipeptidase activity	3	1	0.04	0.04182
GO:0000016	lactase activity	24	2	0.34	0.04463
GO:0008422	beta-glucosidase activity	25	2	0.35	0.04807
GO:0008013	beta-catenin binding	59	3	0.83	0.05044
GO:0008010	structural constituent of chitin-based l...	60	3	0.85	0.05256
GO:0004565	beta-galactosidase activity	27	2	0.38	0.05524
GO:0003958	NADPH-hemoprotein reductase activity	4	1	0.06	0.05538
GO:0004557	alpha-galactosidase activity	4	1	0.06	0.05538

Table S27, related to Figures 8e and 8f. Top 50 GO Biological Process Enrichment for Putative *S. exigua* Induced Epialleles

GO.ID	Term	Num in Genome	Num in Cluster	Expected	p-val
GO:0035017	cuticle pattern formation	38	6	0.6	1.50E-06
GO:0048800	antennal morphogenesis	15	4	0.24	6.90E-05
GO:0031637	regulation of neuronal synaptic plastici...	2	2	0.03	0.00025
GO:0021556	central nervous system formation	2	2	0.03	0.00025
GO:0035881	amacrine cell differentiation	5	2	0.08	0.00238
GO:0046548	retinal rod cell development	6	2	0.09	0.00353
GO:0072539	T-helper 17 cell differentiation	6	2	0.09	0.00353
GO:0035336	long-chain fatty-acyl-CoA metabolic proc...	21	3	0.33	0.0041
GO:0046549	retinal cone cell development	7	2	0.11	0.00489
GO:0021930	cerebellar granule cell precursor prolif...	7	2	0.11	0.00489
GO:0040003	chitin-based cuticle development	132	7	2.08	0.00516
GO:0042632	cholesterol homeostasis	46	4	0.72	0.00571
GO:0036315	cellular response to sterol	29	3	0.46	0.00639
GO:0021702	cerebellar Purkinje cell differentiation	8	2	0.13	0.00646
GO:0032501	multicellular organismal process	4754	83	74.76	0.00784
GO:0042574	retinal metabolic process	9	2	0.14	0.00821
GO:0019218	regulation of steroid metabolic process	78	4	1.23	0.01001
GO:0010025	wax biosynthetic process	10	2	0.16	0.01016
GO:0007561	imaginal disc eversion	10	2	0.16	0.01016
GO:0016476	regulation of embryonic cell shape	30	3	0.47	0.0113
GO:0040034	regulation of development, heterochronic	30	3	0.47	0.0113
GO:0042817	pyridoxal metabolic process	11	2	0.17	0.0123
GO:0003383	apical constriction	31	3	0.49	0.01237
GO:0016042	lipid catabolic process	248	10	3.9	0.01242
GO:0045668	negative regulation of osteoblast differ...	32	3	0.5	0.0135
GO:0046477	glycosylceramide catabolic process	32	3	0.5	0.0135
GO:0048731	system development	3032	44	47.68	0.01471
GO:0035316	non-sensory hair organization	64	5	1.01	0.01533
GO:0032023	trypsinogen activation	1	1	0.02	0.01573
GO:0035238	vitamin A biosynthetic process	1	1	0.02	0.01573
GO:2000258	negative regulation of protein activatio...	1	1	0.02	0.01573
GO:1990654	sebum secreting cell proliferation	1	1	0.02	0.01573
GO:1901810	beta-carotene metabolic process	1	1	0.02	0.01573
GO:0048852	diencephalon morphogenesis	1	1	0.02	0.01573
GO:0045796	negative regulation of intestinal choles...	1	1	0.02	0.01573
GO:0007490	tergite morphogenesis	1	1	0.02	0.01573
GO:1903355	negative regulation of distal tip cell m...	1	1	0.02	0.01573
GO:0035695	mitophagy by internal vacuole formation	1	1	0.02	0.01573
GO:0016121	carotene catabolic process	1	1	0.02	0.01573
GO:0016122	xanthophyll metabolic process	1	1	0.02	0.01573
GO:2000635	negative regulation of primary miRNA pro...	1	1	0.02	0.01573
GO:0046370	fructose biosynthetic process	1	1	0.02	0.01573
GO:0050973	detection of mechanical stimulus involve...	1	1	0.02	0.01573
GO:0001869	negative regulation of complement activa...	1	1	0.02	0.01573
GO:0044256	protein digestion	1	1	0.02	0.01573
GO:0010345	suberin biosynthetic process	1	1	0.02	0.01573
GO:0033082	regulation of extrathymic T cell differe...	1	1	0.02	0.01573
GO:0006043	glucosamine catabolic process	1	1	0.02	0.01573
GO:0010949	negative regulation of intestinal phytos...	1	1	0.02	0.01573
GO:0035317	imaginal disc-derived wing hair organiza...	63	4	0.99	0.017

Table S28, related to Figures 8g and 8h. Top 50 GO Molecular Function Enrichment for Putative *S. exigua* Repressed Epialleles

GO.ID	Term	Num in Genome	Num in Cluster	Expected	p-val
GO:0004122	cystathionine beta-synthase activity	4	2	0.01	5.30E-05
GO:0004124	cysteine synthase activity	4	2	0.01	5.30E-05
GO:0098605	selenocystathionine beta-synthase activi...	4	2	0.01	5.30E-05
GO:0030170	pyridoxal phosphate binding	29	3	0.09	8.60E-05
GO:0070025	carbon monoxide binding	5	2	0.02	8.90E-05
GO:0070026	nitric oxide binding	5	2	0.02	8.90E-05
GO:0004181	metallocarboxypeptidase activity	33	3	0.1	0.00013
GO:0050421	nitrite reductase (NO-forming) activity	6	2	0.02	0.00013
GO:1904047	S-adenosyl-L-methionine binding	8	2	0.02	0.00025
GO:0019825	oxygen binding	11	2	0.03	0.00048
GO:0008010	structural constituent of chitin-based l...	60	3	0.18	0.00076
GO:0050459	ethanolamine-phosphate phospho-lyase act...	1	1	0	0.00306
GO:0004252	serine-type endopeptidase activity	118	3	0.36	0.00526
GO:0070224	sulfide:quinone oxidoreductase activity	2	1	0.01	0.00611
GO:0004850	uridine phosphorylase activity	2	1	0.01	0.00611
GO:0009032	thymidine phosphorylase activity	2	1	0.01	0.00611
GO:0020037	heme binding	39	2	0.12	0.00618
GO:0072341	modified amino acid binding	41	2	0.13	0.00681
GO:0015250	water channel activity	3	1	0.01	0.00916
GO:0004356	glutamine synthetase activity	5	1	0.02	0.01521
GO:0008453	alanine-glyoxylate transaminase activity	5	1	0.02	0.01521
GO:0005549	odorant binding	6	1	0.02	0.01823
GO:0005537	D-mannose binding	11	1	0.03	0.03318
GO:0000016	lactase activity	24	1	0.07	0.07105
GO:0008422	beta-glucosidase activity	25	1	0.08	0.0739
GO:0038024	cargo receptor activity	25	1	0.08	0.0739
GO:0004565	beta-galactosidase activity	27	1	0.08	0.07958
GO:0017042	glycosylceramidase activity	28	1	0.09	0.08241
GO:0015645	fatty acid ligase activity	29	1	0.09	0.08523
GO:0008201	heparin binding	34	1	0.1	0.09921
GO:0031625	ubiquitin protein ligase binding	185	2	0.57	0.10874
GO:0004620	phospholipase activity	43	1	0.13	0.12385
GO:0042802	identical protein binding	1049	4	3.21	0.41327
GO:0004888	transmembrane signaling receptor activit...	192	1	0.59	0.4495
GO:0042803	protein homodimerization activity	587	2	1.8	0.54714
GO:0043168	anion binding	797	4	2.44	0.88343
GO:0016740	transferase activity	1227	5	3.76	0.93724
GO:0016154	pyrimidine-nucleoside phosphorylase acti...	2	1	0.01	1
GO:0060089	molecular transducer activity	295	1	0.9	1
GO:0036094	small molecule binding	2483	5	7.6	1
GO:0019899	enzyme binding	1383	2	4.23	1
GO:0042302	structural constituent of cuticle	66	3	0.2	1
GO:0015267	channel activity	230	1	0.7	1
GO:0016298	lipase activity	92	1	0.28	1
GO:0005539	glycosaminoglycan binding	59	1	0.18	1
GO:1901681	sulfur compound binding	110	3	0.34	1
GO:0140096	catalytic activity, acting on a protein	1181	6	3.61	1
GO:0016672	oxidoreductase activity, acting on a sul...	5	1	0.02	1
GO:0005198	structural molecule activity	464	3	1.42	1
GO:0070279	vitamin B6 binding	29	3	0.09	1

Table S29, related to Figures 8g and 8h. Top 50 GO Biological Process Enrichment for Putative *S. exigua* Repressed Epialleles

GO.ID	Term	Num in Genome	Num in Cluster	Expected	p-val
GO:0006535	cysteine biosynthetic process from serin...	4	2	0.01	6.50E-05
GO:0043418	homocysteine catabolic process	5	2	0.02	0.00011
GO:0006565	L-serine catabolic process	5	2	0.02	0.00011
GO:0042262	DNA protection	6	2	0.02	0.00016
GO:0070814	hydrogen sulfide biosynthetic process	7	2	0.02	0.00022
GO:0009403	toxin biosynthetic process	7	2	0.02	0.00022
GO:0019346	transsulfuration	7	2	0.02	0.00022
GO:0019448	L-cysteine catabolic process	8	2	0.03	0.0003
GO:0060351	cartilage development involved in endoch...	13	2	0.04	0.00082
GO:0001958	endochondral ossification	15	2	0.05	0.0011
GO:0051593	response to folic acid	15	2	0.05	0.0011
GO:0001974	blood vessel remodeling	28	2	0.09	0.00387
GO:0006801	superoxide metabolic process	36	2	0.12	0.00634
GO:0060135	maternal process involved in female preg...	36	2	0.12	0.00634
GO:0006218	uridine catabolic process	2	1	0.01	0.00669
GO:0021587	cerebellum morphogenesis	37	2	0.12	0.00669
GO:0070813	hydrogen sulfide metabolic process	10	3	0.03	0.00926
GO:0040003	chitin-based cuticle development	132	3	0.44	0.0094
GO:0046104	thymidine metabolic process	5	1	0.02	0.01665
GO:0070221	sulfide oxidation, using sulfide:quinone...	5	1	0.02	0.01665
GO:0003018	vascular process in circulatory system	61	2	0.2	0.01751
GO:0043506	regulation of JUN kinase activity	62	2	0.21	0.01806
GO:0006833	water transport	6	1	0.02	0.01995
GO:0006554	lysine catabolic process	8	1	0.03	0.02651
GO:0019236	response to pheromone	8	1	0.03	0.02651
GO:0006212	uracil catabolic process	8	1	0.03	0.02651
GO:0043097	pyrimidine nucleoside salvage	10	1	0.03	0.03303
GO:0006646	phosphatidylethanolamine biosynthetic pr...	11	1	0.04	0.03628
GO:0009404	toxin metabolic process	22	3	0.07	0.04549
GO:0030574	collagen catabolic process	16	1	0.05	0.05234
GO:0036158	outer dynein arm assembly	17	1	0.06	0.05553
GO:0006654	phosphatidic acid biosynthetic process	17	1	0.06	0.05553
GO:0016201	synaptic target inhibition	17	1	0.06	0.05553
GO:0071353	cellular response to interleukin-4	17	1	0.06	0.05553
GO:0009068	aspartate family amino acid catabolic pr...	18	1	0.06	0.0587
GO:0071456	cellular response to hypoxia	118	2	0.4	0.05882
GO:0035150	regulation of tube size	118	2	0.4	0.05882
GO:0046328	regulation of JNK cascade	123	2	0.41	0.06328
GO:0010045	response to nickel cation	24	1	0.08	0.07752
GO:0032872	regulation of stress-activated MAPK casc...	144	2	0.48	0.08324
GO:0044245	polysaccharide digestion	26	1	0.09	0.08371
GO:0006541	glutamine metabolic process	26	1	0.09	0.08371
GO:1901657	glycosyl compound metabolic process	127	2	0.43	0.08464
GO:0046477	glycosylceramide catabolic process	32	1	0.11	0.10205
GO:0010040	response to iron(II) ion	32	1	0.11	0.10205
GO:0006220	pyrimidine nucleotide metabolic process	32	1	0.11	0.10205
GO:0006508	proteolysis	987	6	3.31	0.1034
GO:0060042	retina morphogenesis in camera-type eye	44	1	0.15	0.13767
GO:0010288	response to lead ion	44	1	0.15	0.13767
GO:0042073	intraciliary transport	45	1	0.15	0.14058
